# Generalizing cell segmentation and quantification

**DOI:** 10.1186/s12859-017-1604-1

**Published:** 2017-03-23

**Authors:** Zhenzhou Wang, Haixing Li

**Affiliations:** State Key Laboratory for Robotics, Shenyang Institute of Automation, Chinese Academy of Sciences, Shenyang, China

**Keywords:** Boundary filtering, Noise blob filtering, Threshold selection, Calibration, Iterative erosion

## Abstract

**Background:**

In recent years, the microscopy technology for imaging cells has developed greatly and rapidly. The accompanying requirements for automatic segmentation and quantification of the imaged cells are becoming more and more. After studied widely in both scientific research and industrial applications for many decades, cell segmentation has achieved great progress, especially in segmenting some specific types of cells, e.g. muscle cells. However, it lacks a framework to address the cell segmentation problems generally. On the contrary, different segmentation methods were proposed to address the different types of cells, which makes the research work divergent. In addition, most of the popular segmentation and quantification tools usually require a great part of manual work.

**Results:**

To make the cell segmentation work more convergent, we propose a framework that is able to segment different kinds of cells automatically and robustly in this paper. This framework evolves the previously proposed method in segmenting the muscle cells and generalizes it to be suitable for segmenting and quantifying a variety of cell images by adding more union cases. Compared to the previous methods, the segmentation and quantification accuracy of the proposed framework is also improved by three novel procedures: (1) a simplified calibration method is proposed and added for the threshold selection process; (2) a noise blob filter is proposed to get rid of the noise blobs. (3) a boundary smoothing filter is proposed to reduce the false seeds produced by the iterative erosion. As it turned out, the quantification accuracy of the proposed framework increases from 93.4 to 96.8% compared to the previous method. In addition, the accuracy of the proposed framework is also better in quantifying the muscle cells than two available state-of-the-art methods.

**Conclusions:**

The proposed framework is able to automatically segment and quantify more types of cells than state-of-the-art methods.

**Electronic supplementary material:**

The online version of this article (doi:10.1186/s12859-017-1604-1) contains supplementary material, which is available to authorized users.

## Background

Imaging of cells in biology are becoming more and more popular with the fast development of microscopy and nanotechnology [1–7]. In different applications, different ways had been utilized to separate the imaged cells and they usually took the researchers great effort. As a powerful tool, the image processing technology is becoming more and more important for the segmentation, quantification and analysis of microscopy data [8, 9]. In different applications, the forms, the dimensions of the cells and their gray-level distributions vary significantly, which makes the segmentation task challenging. In many applications, the cells are frequently neighboring or overlapping on each other, which makes the quantification difficult. In this paper, we propose a generalized framework for robust segmentation and quantification of different types of cells imaged in different biological applications.

In the past decades, image processing technology has been utilized widely in segmenting and quantifying different types of cells. The absence of a generalized framework for different types of cell images makes the research work application specific instead of convergent to a common solution. Different methods were proposed and claimed to be superior in segmenting a class of cells. These methods include watershed method [10–12], region growing based method [13], morphological method [14, 15], clustering based method [16], contour based method [17], multilayer segmentation based method [18], pattern modeling based method [19], supervised learning method [20], morphological watershed based method [21], inference based method [22] and methods that combine the threshold selection and morphology techniques [23–25]. However, the performance and applicability of most of these methods are very limited because they are diverging rather than convergent to a generalized solution to address so many types of cells. To overcome this drawback, the author has proposed a new approach to segment and quantify different types of cells or nanoparticles based on the general property of the cell images: global intensity distribution and local gradient [24], which is more versatile than the referenced state of the art methods. The approach proposed in [24] evolves the method proposed in [25] and makes it to be able to segment and quantify more types of cells or nanoparticles. One fundamental improvement of [24] compared to [25] is that the threshold selection method used in [25] was improved to be able to segment more types of cells or nanoparticles robustly. However, the details of how to apply the proposed threshold selection method with practical cell images are not addressed adequately in [24]. In this paper, we design the practical algorithm to apply the threshold selection method proposed in [24] to segment the practical cell images. In addition, we calibrate more parameters than [24] to guarantee the robust segmentation.

A more important goal of this paper is to propose a generalized framework to segment and quantify different types of cells imaged in different systems with higher accuracy compared to the past work [10–25]. To this end, we tested more cell images in addition to the muscle cell images in [25]. Some imaged cell images have artifacts or the segmentation results contain too much noise. Consequently, the segmented cells contain shape noises which will increase the number of the eroded seeds by the iterative erosion method proposed in [25], which will affect the final quantification accuracy. To eliminate these shape noises, we propose a Fourier Transformation based shape filter and it could decrease the wrong quantification effectively. In addition to the shape filter, we also propose a blob filter that could remove the line shape noise blobs effectively. For the muscle cell images [25], two cases are defined in the union method based on the image characteristics. For the generalized framework to segment more types of cell images, three cases are defined in the union method in this paper. To verify the advantages of the proposed generalized framework over the past research work [10–25], we give both the qualitative results and the quantitative results.

## Methods

### The generalized framework

**Fig. 1:**
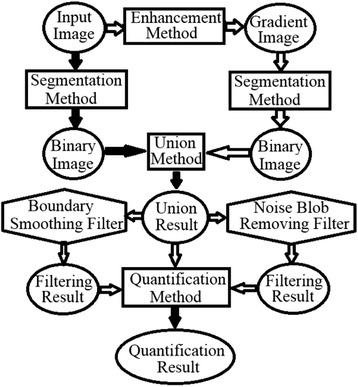
Flowchart of the proposed framework (The *black solid arrow* denotes the obligatory operation while the *white hollow arrow* denotes the optional operation)

The proposed framework for segmentation and quantification of the cells is illustrated in Fig. 1. In the framework, the content in the ellipse vary depending on the input image to be processed while the content in the rectangle are the proposed algorithms and they remain the same for different types of cells. The input image denotes the original cell image. The gradient image is obtained after edge enhancement. Both the input image and the gradient image are segmented by the threshold selection method automatically to get the binarized image and the constraint edge image, respectively. The segmentation result is obtained by unifying the binarized image and the negative constraint edge image. The noise blob removing filter is used to eliminate the thread-like or small noise blobs. The boundary smoothing filter is used to remove the noise contained in the extracted boundary. The quantification method is used to obtain the quantification result after identifying each cell individually. Except the segmentation method, the union method and the quantification method, all the other methods are optional based on the characteristics of the cells. For each type of cell, the methods are selected and then applied one by one in the framework and they need to be prepared and calibrated carefully before the framework could segment and quantify the cells automatically.

### The enhancement method

The gradient image is generated by the enhancement method. In the current framework, we generate the gradient image using the Sobel operators. Firstly, the Sobel operator is applied to the cell image along the row direction to get the horizontal gradient components, *I*_*x*_. Secondly, the Sobel operator is applied to the cell image along the column direction to get the vertical gradient components, *I*_*y*_. Thirdly, the gradient image is formed by the following equation.

1 $$ {I}_g\left( h, j\right)=\sqrt{{I_x}^2\left( h, j\right)+{I_y}^2\left( h, j\right)} $$

where *h* = 1, …, *H*; is the index of the pixel along the column direction and *j* = 1, …, *J*; is the index of the pixel along the row direction. *H* × *J* denotes the dimension of the cell image.

### The segmentation method

The segmentation method should be flexible and robust enough for a vast variety of cell images. We tested all the available state of the art segmentation methods [26–33] to segment the cell images and the generated gradient images. Unfortunately, we did not find any state of the art segmentation method that could yield adequate accuracy consistently for so many types of cell images. A more versatile, flexible and generalized image segmentation method has been proposed in [25] to produce acceptable segmentation results consistently for many types of muscle cell images. However, the histogram modalities of the muscle cell images are similar and so are the modalities of their gradient images, which makes the image segmentation less challenging compared to segmenting more divergent types of cell images. Fortunately, the flexibility of the previously proposed threshold selection method makes it adjustable for different types of images by varying the its parameters. Hence, we introduce the process of calibration in this paper to find the optimal parameters of the threshold selection method for each specific type of cell image. The previously proposed threshold selection method could be summarized as follows.

The threshold is calculated from the slope difference distribution of the normalized histogram. The histogram is assumed as Gaussian-mixture distributions in this research work. We define the slope difference distribution of the image as the variation rate of the normalized histogram and it could be computed by the following steps.

**Step 1**, Assuming the image is non-negative, the cell image is modified by rearranging its gray-scale values in the interval [0, 255] with the following equation.

2 $$ {I}^{\prime}\left( h, j\right)=\frac{255\times I\left( h, j\right)}{ \max (I)};\kern0.75em  h=1,\dots, H; j=1,\dots, J $$

where *H* × *J* is the resolution of the cell image, *I. h* is the index of the pixel along the vertical direction of the cell image and *j* is the index of the pixel along the horizontal direction of the cell image. Here, 255 is used for convenience because most gray images have the maximum value of 255. 255 could be changed to other values based on the application requirements.

**Step 2**, the histogram distribution *P*(*x*) of the modified cell image, *I*′ is normalized by the following equations:

3 $$ P\left( x= m\right) = \frac{N_m}{\ {N}_l}; m=0,\dots,\ 255; $$

4 $$ l=\underset{\tilde{l}\in \left[0,255\right]}{\mathrm{argmax}}\ {N}_{\tilde{l}} $$

where *N*_*l*_ denotes the maximum frequency that occurs at *l* in the interval [0, 255]. *N*_*m*_ denotes the frequency of the pixel value *m*.

**Step 3**, after the histogram distribution is normalized, it is then filtered in the frequency domain. Firstly, the normalized histogram distribution, *P*(*x*) is transformed into the frequency domain with the Discrete Fourier Transformation (DFT):

5 $$ F(k)={\displaystyle \sum_{x=0}^{255}} P(x){e}^{- i\frac{2\pi kx}{255}}; k=0,\dots, 255 $$

Then, we select the low frequency parts from 1 to *L* and eliminate the rest of high frequency parts with the following equation.

6 $$ {F}^{\prime }(k)=\left\{\begin{array}{c}\hfill F(k); k=0,1,\dots, W\hfill \\ {}\hfill F(k); k=255- W,\dots, 254,255\hfill \\ {}\hfill 0; k= W+1,\dots, 255- W-1\hfill \end{array}\right. $$

where *W* the bandwidth of the low pass DFT filter and it is going to be determined by the calibration process.

After the above equation is performed to filter histogram distribution in the frequency domain, we transform the smoothed histogram distribution back into spatial domain by the following equation.

7 $$ {P}^{\prime }(x)=\left|\frac{1}{255}{\displaystyle \sum_{k=0}^{255}}{F}^{\prime }(k){e}^{i\frac{2\pi xk}{255}}\right|; x=0,\dots, 255 $$

where *P*′(*x*) is the filtered and smoothed histogram.

**Step 4**, for each point, *i* on *P*′(*x*), there are two slopes, *a*_1_(*i*) and *a*_2_(*i*). They are on the left side and the right side of the point, *i* respectively. They could be computed by a fitted line model with *N* adjacent points at each side and the parameter *N* will also be determined by the calibration process. The line model is formulated as:

8 $$ {y}_i= a{x}_i+ b $$

9 $$ {\left[ a, b\right]}^T={\left({B}^T B\right)}^{-1}{B}^T Y $$

10 $$ B=\kern1.5em \left[\begin{array}{cc}\hfill {x}_1\hfill & \hfill 1\hfill \\ {}\hfill {x}_2\hfill & \hfill 1\hfill \\ {}\hfill \begin{array}{c}\hfill \vdots \hfill \\ {}\hfill {x}_N\hfill \end{array}\hfill & \hfill \begin{array}{c}\hfill \vdots \hfill \\ {}\hfill 1\hfill \end{array}\hfill \end{array}\right] $$

11 $$ Y=\kern0.75em {\left[{y}_1,{y}_2,\dots,,, {y}_N\right]}^T $$

When the *N* fitting points are on the left side of the point *i*, the slope *a* equals *a*_1_(*i*). When the *N* fitting points are on the right side of the point *i*, the slope *a* equals *a*_2_(*i*). Both slopes are computed by Eq. 9.

Accordingly, the slope difference of the point *i* is computed by the following equation.

12 $$ s(i)={a}_2(i)-{a}_1(i); i= N+1,\dots,\ 255- N $$

**Fig. 2:**
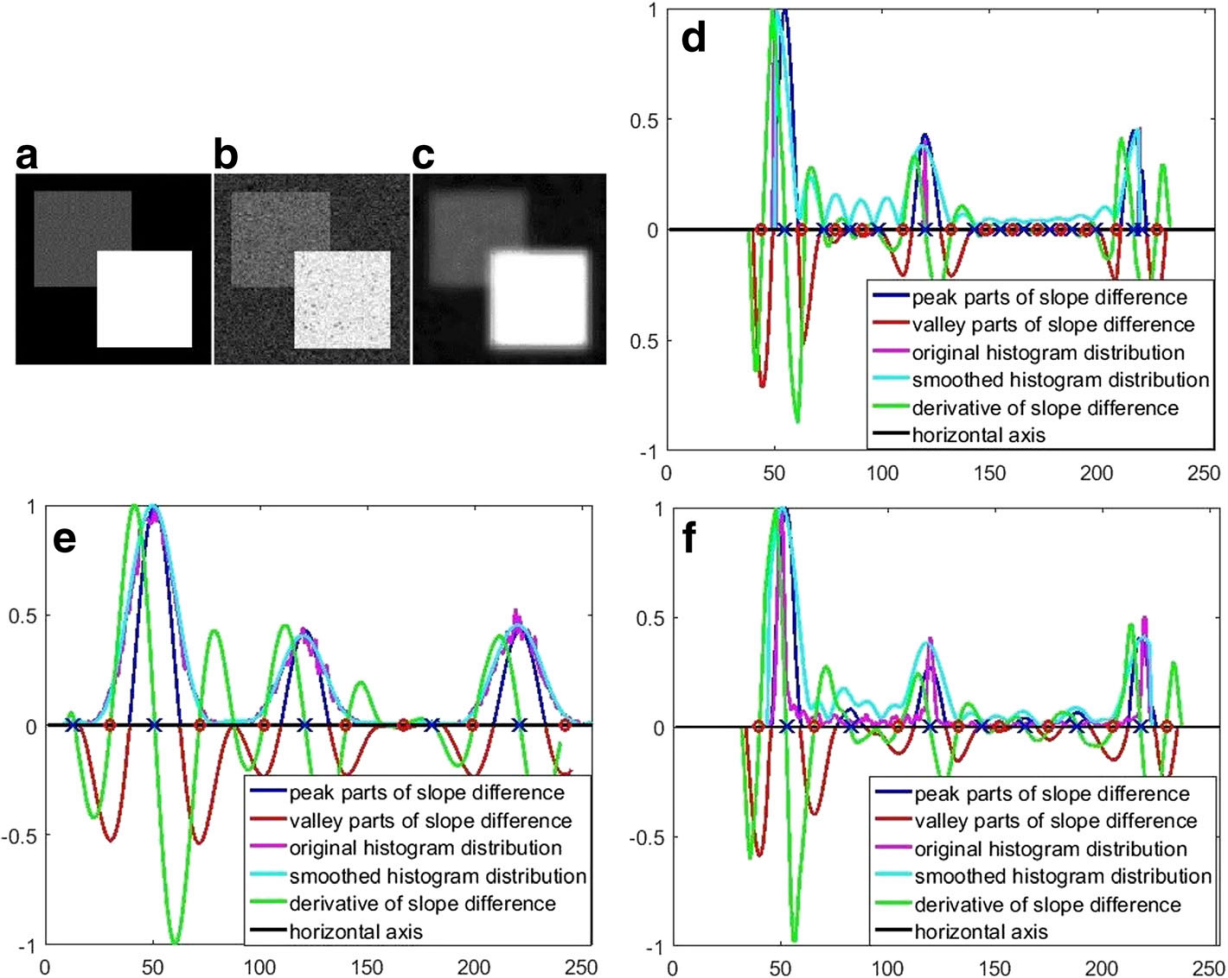
Demonstration of slope difference distribution. **a** The first synthesized image. **b** The second synthesized image. **c** The third synthesized image. **d** Slope difference distribution of first synthesized image. **e** Slope difference distribution of second synthesized image. **f** Slope difference distribution of third synthesized image

The continuous version as *s*(*i*) is defined as the slope difference distribution. Setting its derivative to zero, we could get the *N*_*v*_ valleys *V*_*i*_; *i* = 1, …, *N*_*v*_ with greatest local variations and *N*_*p*_ peaks *P*_*i*_; *i* = 1, 2, …, *N*_*p*_ with greatest local variations of the slope difference distribution. Not all peaks or valleys are caused by the histogram variations because the smoothing process by the low-pass DFT filter might produce small harmonics when significant parts of the original histogram remain the same or close to the horizontal axis. Consequently, these harmonics produce pseudo peaks and valleys. Fortunately, the pseudo peaks or valleys are much smaller compared to the real peaks or valleys. The real peaks or valleys could be distinguished from the pseudo ones easily based on their magnitudes. On the other hand, the produced harmonics avoid the possible ill-conditions of the matrix inverse operation in Eq. 9. The matrix inverse operation will become ill-conditioned when the *N* fitting points are from a horizontal line. The horizontal parts in the histogram are replaced with harmonics after DFT filtering. We demonstrate the slope difference distribution with three synthesized images in Fig. 2. The first synthesized image is an image with two objects as shown in Fig. 2a. The grayscale of the background equals 50, the grayscale of the dark object equals 120 and the grayscale of the bright object equals 220. Its slope distribution is demonstrated in Fig. 2d. The original histogram distribution consists of three isolated peaks. After DFT filtering, the histogram distribution become continuous with small harmonics that produce many small pseudo peaks and valleys. There are three real peaks and six real valleys and their magnitudes are much greater than those of the pseudo ones. The peaks and valleys are denoted by the blue crosses and red circles respectively in Fig. 2d. The second image is synthesized by adding Gaussian noise to the first synthesized image and it is shown in Fig. 2b. Its slope difference distribution is shown in Fig. 2e. As can be seen, its original histogram is continuous with less parts on the horizontal axis. As a result, less harmonics and less pseudo peaks and valleys are generated. The third image is synthesized by blurring the second synthesized image with an iterative moving average filter and it is shown in Fig. 2c. Its slope difference distribution is shown in Fig. 2f. As can be seen, its original histogram is also continuous. However, many parts are close to the horizontal axis. As a result, many pseudo peaks and pseudo valleys occur. From all these results, it is seen that the real peaks or valleys could be easily distinguished from the pseudo peaks or valleys. For most practical images, their histograms are usually continuous without significant parts close to the horizontal axis or remain the same, thus no pseudo peaks or valleys will occur. For the image with known number of pixel classes *K*_*c*_, the rule to select the peaks is as follows. Firstly, all the peaks are sorted in the magnitude descending order. Secondly, the first *K*_*c*_ peaks are then selected as the real peaks.

The slope difference distribution has three fundamental properties that help to design the threshold selection process.

***Property 1***: in situations where the histogram distribution of background and the histogram distribution of the cells are both Gaussian distributed, the valley positions between the background and the object on the slope difference distribution change monotonically with the number of the fitted points *N* in the line model while the peak positions are almost the same when the parameter, *N* is changed gradually. In the experiments, we found that this property holds only when the histogram is filtered by the designed filter with the bandwidth parameter *W* calibrated and chosen properly for each specific type of image. When we used other filters, for instance, the finite impulse response (FIR) filter and the infinite impulse response (IIR) filter, both the peaks and the valleys of the slope difference distribution change irregularly. Hence, we conclude that the Fourier Transformation based filtering is capable of removing the high frequency noises effectively while maintaining the shape of the histogram well. However, the FIR filter and IIR filter lack this capability and will change the shape of smoothed histogram undesirably. Consequently, they cause the peaks of the slope difference distribution to change randomly.

***Property 2***: the peaks of the slope difference distribution correspond to the cluster centers of the objects or the background while the valleys correspond to the thresholds that could separate the objects and the background.

***Property 3***: the fitting number *N* of line model determines the number of the peaks of the slope difference distribution. A large *N* value could suppress small peaks and unify adjacent peaks into one peak.

The proposed threshold selection method is flexible and has some changeable manual inputs that could be adjusted to meet different segmentation requirements. The first manual input defines how many pixel classes the image contains. The default value of it is 2, which indicates that there are one object class and one background class. The second manual input defines what classes to segment. When the user wants to separate the background class and all the objects classes, it is defined as ***Case 1***. When the user wants to separate the first object class and the second object class along the pixel increase direction, it is defined as ***Case 2. Case 3*** is defined as the separation between the second object class and the third object class. In the same way, other cases are defined. ***Case 1*** is default case. The third input is how many points the line model uses to fit the line and the fourth input is the bandwidth of the low pass filter. To determine the third and fourth inputs for each type of cell images before segmentation, we calibrate the threshold selection method based on the popular F-measure. For a specific type of cell images, the calibration process is summarized as follows.

For a specific type of cell images, we select several typical images and obtain the ground truth manual segmentation results for these images.

Then, we vary the value of the third input, the parameter *N* in Eq. (11) from 3 to 60 and the fourth input, the parameter *W* in Eq. (6) from 2 to 50. We compute the F-measure, *F*_*m*_ of the automatic segmentation result by the threshold selection method and the manual segmentation result for each pair parameters (*N*, *W*) by the following equations.

13 $$ {F}_m=\frac{2\times P\times R}{P+ R} $$

14 $$ P=\frac{S_{S D}{\displaystyle \cap }{S}_m}{S_m} $$

15 $$ R=\frac{S_{S D}{\displaystyle \cap }{S}_m}{S_{S D}} $$

where *S*_*m*_ is the ground truth manual segmentation and *S*_*SD*_ is the automatic segmentation result by the threshold selection method. We choose the pair of parameters (*N*, *W*) that yields the largest *F*_*m*_.

During segmentation of a great of variety of cell images, it might be inconvenient to obtain the benchmark manual segmentation from the cytologist for each type of cell image. Here, we propose a rational calibration method in the absence of benchmark manual segmentation result based on ***Property 3*** of the slope difference distribution.

**Fig. 3:**
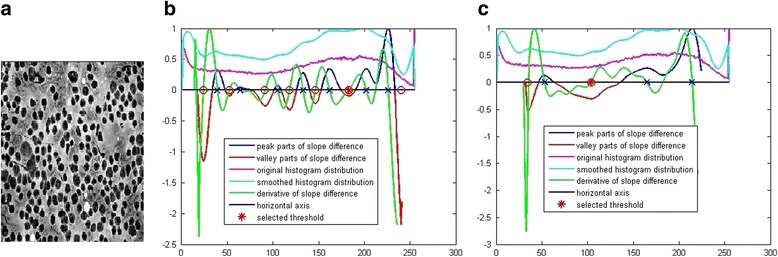
Demonstration of threshold selection by the proposed efficient calibration method. **a** The gray image. **b** Threshold selection process with *N* = 15. **c** Threshold selection process with *N* = 31

**Step 1:** we determine how many pixel classes the image contains rationally. Here, we give an example of cell image with three pixel classes: the black cell, the gray clutters and the brighter background as shown in Fig. 3a. There are small abrupt parts with pixel values close to 255 in the original histogram distribution, which affects the normalization of the histogram and makes most parts of the histogram below 0.5. After DFT filtering, this bad effect is removed and the normalized histogram becomes much better.

**Step 2:** we use the default value, *N* = 15 and *W* = 10 to calculate the thresholds visually as shown in Fig. 3b. It is seen that there are 7 peaks instead of 3 peaks existing in the calculated slope difference distribution.

**Step 3:** we increase the value of *N* until there are only 3 peaks in the calculated slope difference distribution as shown in Fig. 3c.

**Step 4:** we select the threshold according to rules described above from the calculated slope difference distribution with three peaks.

Please note that the proposed rational calibration method is used only when the benchmark manual segmentation is not available. When the benchmark data is available, the calibration method based on the F-measure is used because it is more robust than the proposed rational method.

### The union method

We calculate the threshold, *T*_0_ for the modified input cell image, *I*′ with the efficiently calibrated threshold selection method. Then, the modified cell image is binarized by the following equation.

16 $$ {S}_I=\left\{\begin{array}{c}\hfill 1;\kern0.5em {I}^{\prime}\ge {T}_0\hfill \\ {}\hfill 0;\kern0.5em {I}^{\prime }<{T}_0\hfill \end{array}\right. $$

We calculate the threshold, *T*_1_ for the gradient image, *I*_*g*_ with the efficiently calibrated threshold selection method. Then, the gradient image is binarized as follows.

17 $$ {S}_g=\left\{\begin{array}{c}\hfill 1;\kern0.5em {I}_g\ge {T}_1\hfill \\ {}\hfill 0;\kern0.5em {I}_g<{T}_1\hfill \end{array}\right. $$

After calculating the two segmentations, *S*_*I*_ and *S*_*g*_, we compute their union segmentation *S*_*u*_ in three cases. For one specific type of cell image, the user need to decide which case it belongs to. For the cell images with a lot of overlapping/neighboring boundaries and their segmented boundaries are not closed for each cell, we define that they belong to *Case 1* and most cell images belong to this case. For this case, the segmentation method is formulated as follows to utilize the segmentations of the gradient image and original image, *S*_*g*_ and *S*_*I*_.

18 $$ {S}_u=\left\{\begin{array}{c}\hfill 1;\kern0.5em  if\ \left({S}_I=1\right)\  and\ \left({S}_g=0\right)\hfill \\ {}\hfill 0;\kern5.75em  else\hfill \end{array}\right. $$

For the cell images with many overlapping or neighboring boundary and the segmented boundary for each cell is closed, we define that they belong to *Case 2*. For instance, many muscle cell images belong to this case. For this case, the segmentation method is formulated as follows to utilize the segmentation of the gradient image, *S*_*g*_ alone.

19 $$ {S}_u=\left\{\begin{array}{c}\hfill 1;\kern0.5em  if\ {S}_g=0\hfill \\ {}\hfill 0;\kern3em  else\hfill \end{array}\right. $$

For the cell images with little overlapping or neighboring boundary, we define that they belong to *Case 3*. In this case, we formulate the segmentation method as follows to make use of the segmentation of the original input image, *S*_*I*_ only.

20 $$ {S}_u=\left\{\begin{array}{c}\hfill 1;\kern0.5em  if\ {S}_I=1\hfill \\ {}\hfill 0;\kern2.75em  else\hfill \end{array}\right. $$

### The noise blob removing filter

**Fig. 4:**
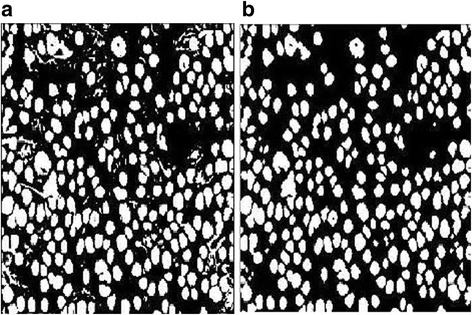
Demonstration of removing the noise blobs by the proposed filter. **a** The result of union segmentation. **b** The filtering result by the proposed noise blob removing filter

In many situations, there are a lot of noise blobs in the union segmentation *S*_*u*_, which might affect the accuracy of the automatic quantification process. One big difference between the noise blob and the cell blob is that the noise blob is usually more tenuous than the cell blob as shown in Fig. 4a, where the noise blobs are threadlike while the cell blobs are relatively massive. Hence, we propose the following filter to remove this kind of noise blobs.

**Step 1:** Erode the union segmentation, *S*_*u*_ morphologically by the following equations.

21 $$ {S_u}^{\prime }={S}_u\ominus B=\left\{ z\Big|{(B)}_z\subseteq {S}_u\right\} $$

22 $$ {(B)}_z=\left\{ c\Big| c= p+ z,\kern0.5em  p\in B\right\} $$

23 $$ {S}_u={S_u}^{\prime } $$

where *B* is the 4-connected structure element with the disk shape and its radius is 1. *p* is the point in the structuring element *B* and *z* is the translation vector.

**Step 2:** Repeat **Step 1** *N*_*l*_ times. The value of *N*_*l*_ is determined by the user and its default value is 3.

**Step 3:** Dilate the union segmentation, *S*_*u*_ morphologically by the following equations.

24 $$ {S_u}^{\prime }={S}_u\oplus B=\left\{ z\Big|{\left({B}^s\right)}_z\cap {C_j}^i\ne \varnothing \right\} $$

25 $$ {S}_u={S_u}^{\prime } $$

where *B* ^*s*^ denotes the symmetric or supplement of *B*.

**Step 4:** Repeat **Step 3** *N*_*l*_ times.

The functionality of the above filter is to remove the threadlike or small blobs by a repeating morphological erosion process at first. Then, a morphological dilation process with the same repeating times is used to restore the eroded cell blobs. Figure 4b shows the result of applying the above filter to the union segmentation shown in Fig. 3a. As can be seen, the tenuous noise blobs are removed effectively while the cell blobs are maintained well.

### The boundary smoothing filter

There are some imaged cell images with poor quality or with imaging artifacts. As a result, the segmentation results produce a lot of boundary noise that is defined as the elements e.g. the boundary roughness or holes inside the segmented cells that make the cells irregular. The irregular cells will produce more seeds during the iterative erosion process proposed in [24, 25], which will increase the quantified number. To eliminate the shape noises, we propose a boundary smoothing filter as follows.

**Step 1:** Exact the boundaries {*x*_*i*_^*j*^, *y*_*i*_^*j*^}; *i* = 1, 2, …, *N*_*j*_ of all the binary blobs and the holes inside the blobs in the union segmentation *S*_*u*_. *j* denotes the index of the binary blobs and the holes inside the blobs. *i* denotes the index of the point in the *j* th extracted boundary for the *j* th binary blob or the hole. *N*_*j*_ denotes the total number of the points in the *j* th extracted boundary.

**Step 2:** For the *j* th boundary, if *N*_*j*_ > *T*_*sn*_, the boundary is valid and will be kept. Otherwise, the boundary is invalid and will be removed. *T*_*sn*_ is the shape noise threshold and it could be computed based on the average size of all the segmented blobs in the image. For a specific type of cell images, the sizes of the cells and the sizes of the noise blobs usually change in different ranges. Offline analysis could find a more accurate size threshold, *T*_*sn*_ to separate cells and the noise blobs robustly.

**Step 3:** For all the valid boundaries, filter them by the Fourier filter defined by Eqs. (5-7). The input is changed from the normalized histogram to *x* coordinates and *y* coordinates of the valid boundaries respectively.

**Step 4:** Using the filtered boundaries to compute binary blobs again and form the filtered blob image, *I*_*fb*_.

### The quantification method

In most cases, there are cells separate from others and there are also cells connected with each other in the filtered blob image, *I*_*fb*_. To identify the cell individually, the same iterative morphological erosion method proposed in [24, 25] is used here.

**Step 1:** Initialize the seeds of all the cells to be the filtered blob image, *I*_*fb*_.

26 $$ {I_b}^1={I}_{fb} $$

**Step 2:** Erode the seeds *I*_*b*_^*i*^ morphologically with the structure element *B* = {(0, 0)} as follows.

27 $$ {I_b}^{\prime }={I_b}^i\ominus B=\left\{ z\Big|{(B)}_z\subseteq {I_b}^i\right\} $$

28 $$ {(B)}_z=\left\{ c\Big| c= p+ z,\kern0.5em  p\in B\right\} $$

where *p* is the point in the structuring element *B* and *z* is the translation vector.

**Step 3:** Then calculate the union of the separated cells that are determined according to their areas. Use them as the updated seeds.

29 $$ {I_c}^{i+1}=\cup C\left(\tilde{J}\right);\tilde{J}=\underset{j}{ \arg } area\left( C(j)\right)<{S}_0 $$

30 $$ {I_b}^{i+1}={I_b}^{\prime }-{I_c}^{i+1} $$

*S*_0_ that is defined as the area threshold to distinguish the area of the cell and the area of noise blob, is computed as the mean area of all the cells after a number of erosions on the segmented cells.

**Step 4:** Use the above steps to erode the segmented cells until the area of each cell is smaller than *S*_0_. At last, the seeds are updated as:

31 $$ {I}_s={\displaystyle \underset{i=1}{\overset{L}{\cup }}}{I_c}^i $$

where *L* denotes the total number of the isolated cells. After all the cells are identified, the coordinate (*x*_*c*_^*k*^, *y*_*c*_^*k*^) of the k *th* cell’s center is computed as:

32 $$ {x}_c^k=\frac{1}{M}{\displaystyle \sum_{j=1}^M}{x}_j^k $$

33 $$ {y}_c^k=\frac{1}{M}{\displaystyle \sum_{j=1}^M}{y}_j^k $$

where *M* is the total number of pixels in the segmented cell and *j* is the pixel index of the segmented cell.

### The algorithm of the generalized framework

The generalized framework is summarized in Algorithm 1.
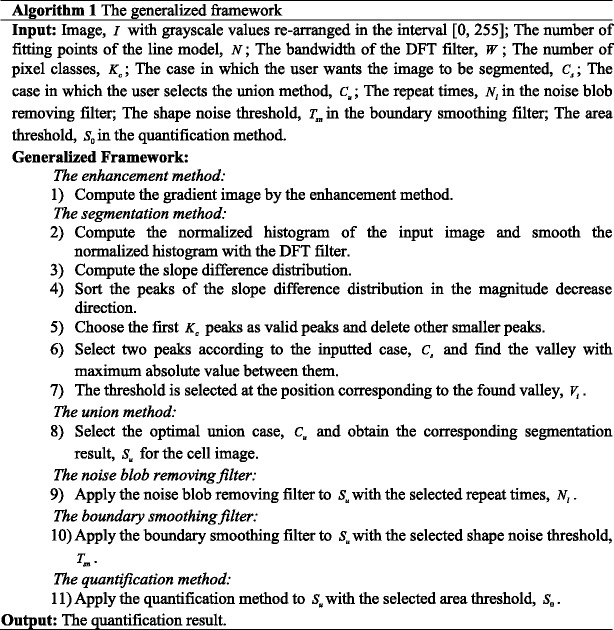


The calibration process based on the F-measure is summarized in Algorithm 2.
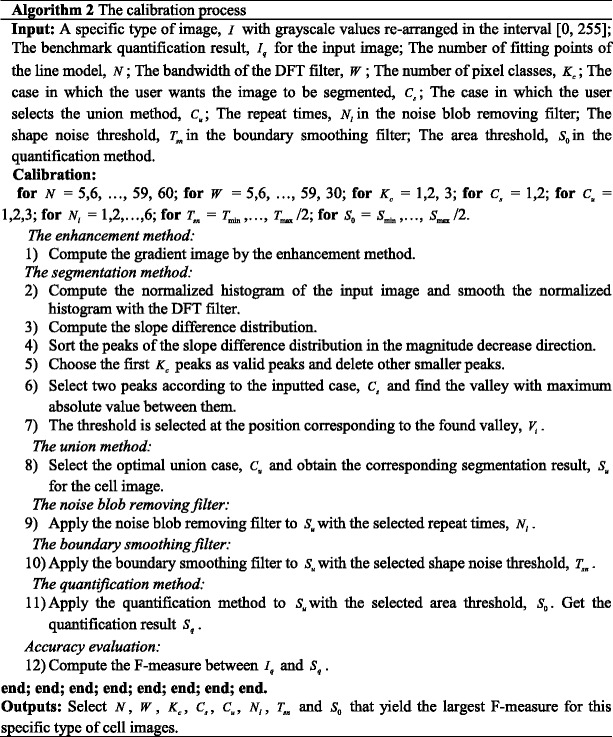


## Results

In this section, we verify the robustness and the generality of the proposed framework with both the muscle cell images used in [24, 25] and other types of cell images.

**Fig. 5:**
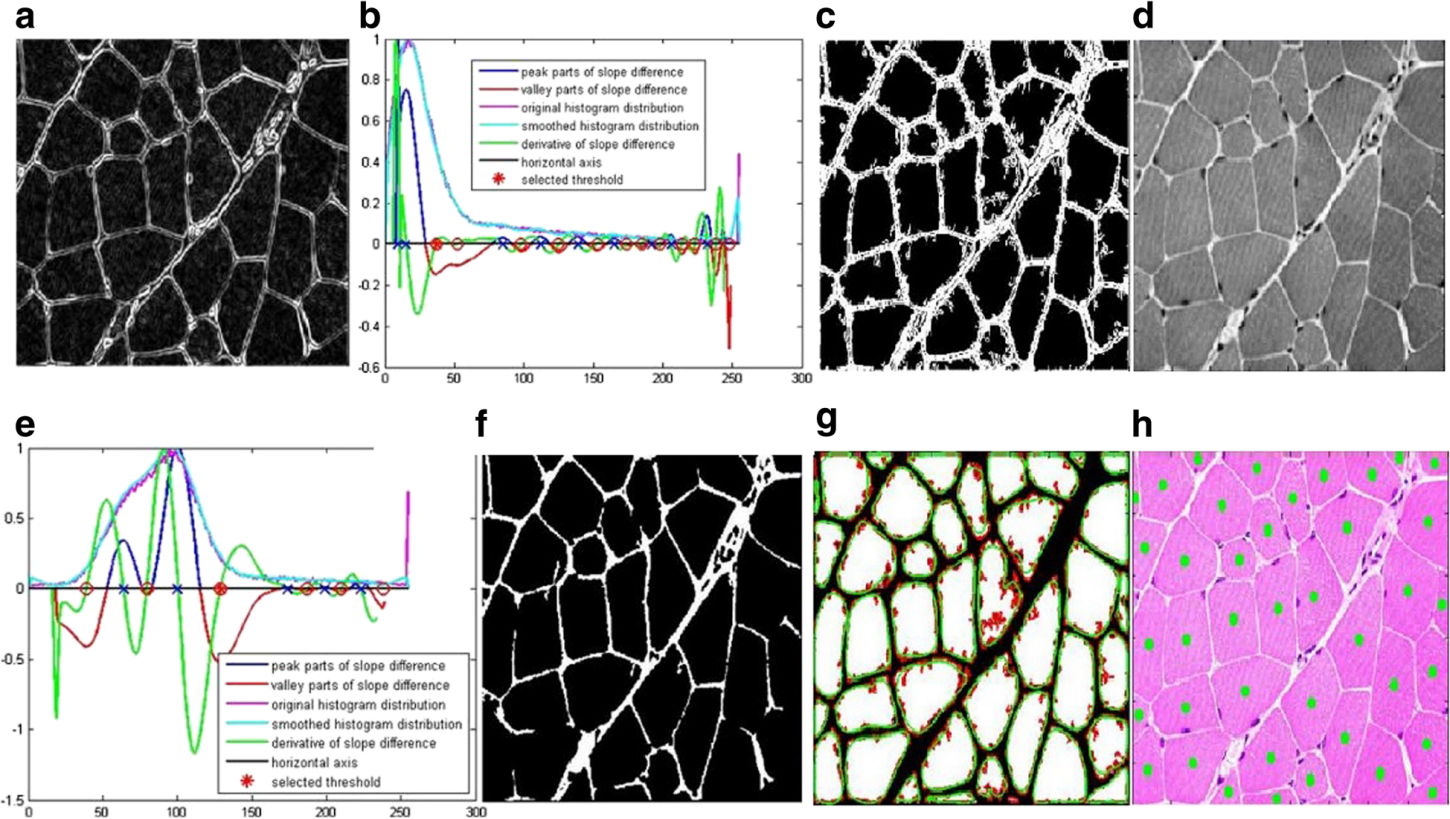
Demonstration of segmentation and quantification by the proposed framework using one tested image from [25]. **a** The gradient image. **b** Threshold selection for the gradient image. **c** Segmentation result of the gradient image. **d** The gray image. **e** Threshold selection for the gray image. **f** Segmentation result of the *gray image*. **g** The *green* filtered shape and the *red* original shape overlaying on the union result of case 1. **h** The quantified cells denoted by the *green dots* overlaying on the original image

**Fig. 6:**
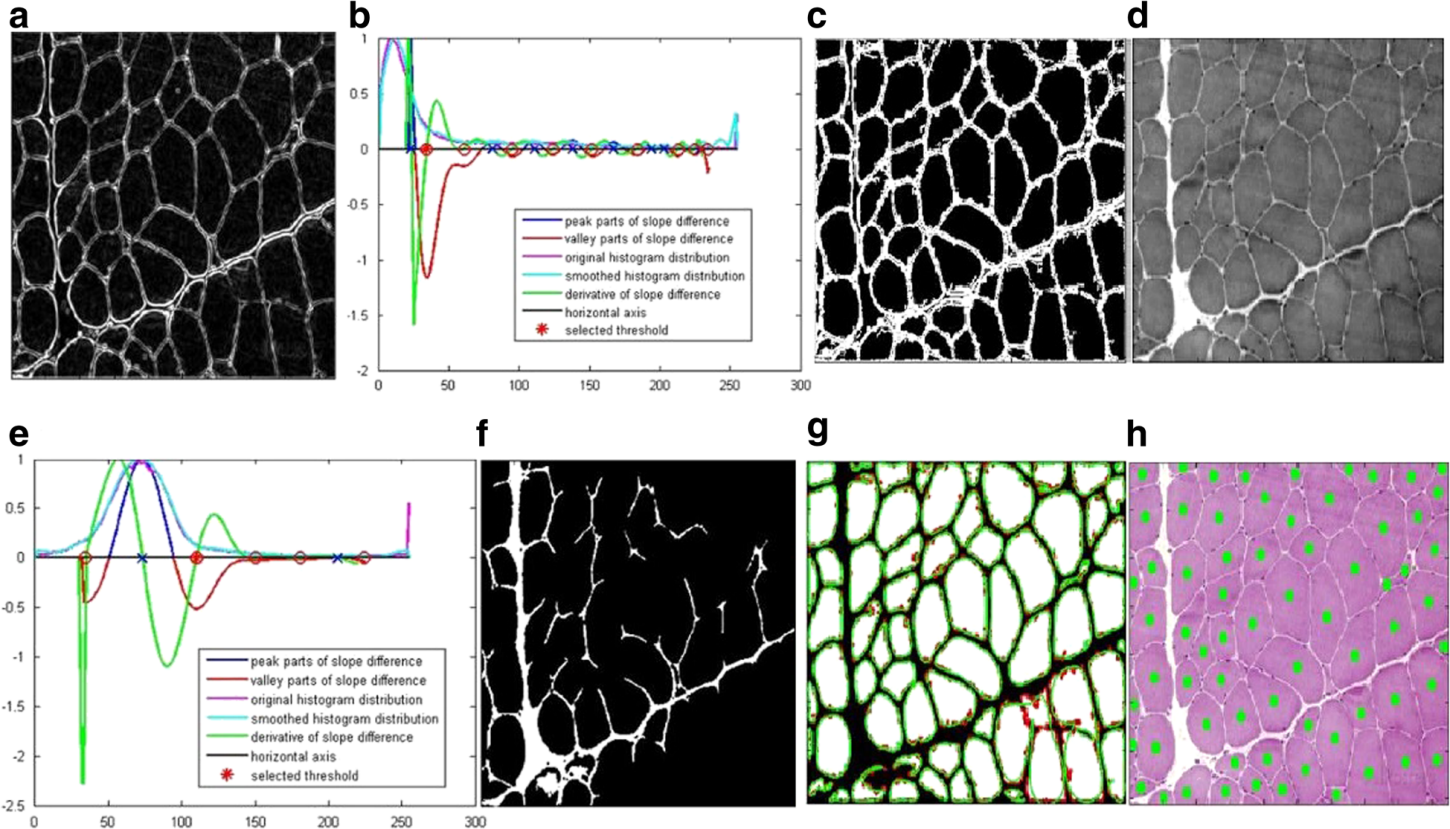
Demonstration of segmentation and quantification by the proposed framework using another tested image from [25]. **a** The gradient image. **b** Threshold selection for the gradient image. **c** Segmentation result of the gradient image. **d** The gray image. **e** Threshold selection for the *gray image*. **f** Segmentation result of the *gray image*. **g** The *green* filtered shape and the *red* original shape overlaying on the union result of case 1. **h** The quantified cells denoted by the *green dots* overlaying on the original image

One big difference between the proposed framework in this paper and the methods proposed in [24, 25] is the inclusion of the boundary smoothing filter. Here, we use two examples of muscle cells to demonstrate the advantages of the proposed framework in this paper over the methods proposed in [24, 25]. Two typical muscle images that have been used to testify the proposed method in [25] are used to show the superiority of the proposed framework in Figs. 5 and 6 respectively. Figure 5a shows the gradient image enhanced from the gray image by Eq. (1). Figure 5b shows the threshold selection process for the gradient image. The smoothed histogram is plotted in cyan. The original histogram is plotted in mauve. The peak part of the slope difference distribution is plotted in blue and the valley part of the slope difference is plotted in red. The derivative of the slope difference is plotted in green and its interception points with the horizontal axis are denoted as blue crosses when they correspond to the peaks of the slope difference. They are denoted as the red circles when they correspond to the valleys of the slope difference. The selected threshold is denoted as the red asterisk. After calibration, the optimal *W* value is chosen as 10 and the optimum *N* value is chosen as 17. Figure 5c shows the segmented edges with the selected threshold. Figure 5d shows the gray image of the muscle cell image and Fig. 4e shows the threshold selection process for it. Figure 5f shows the segmented edges from the gray image. Figure 5g shows the filtered boundary overlaying on the segmentation result by the case 1 union method. Figure 5h shows the cell quantification result overlaying on the original cell image.

**Fig. 7:**
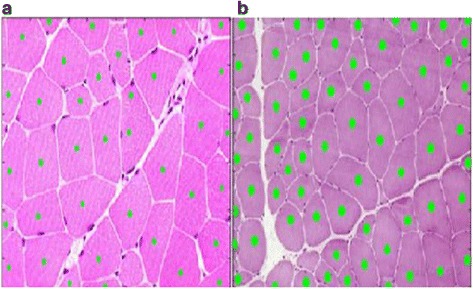
Quantification results of the same two muscle cell images in [25]. **a** The tested muscle cell image in Fig. 5. **b** The tested muscle cell image in Fig. 6

Figure 6a-h show the segmentation and quantification results of another testified muscle cell image in [25]. To compare the quantification accuracy of the generalized framework in this research work and the method previously proposed in [25] more conveniently, we show the quantification results by [25] in Fig. 7. As can be seen, two missing cells in the quantification result of Fig. 7a are quantified correctly in Fig. 5h. In addition, the extra one false quantification in Fig. 7a is avoided in Fig. 5h. Similarly, the quantification result in Fig. 6h are better than that in Fig. 7b.

**Fig. 8:**
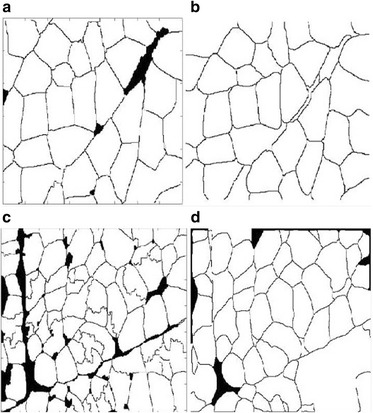
Results of the same two muscle cell images in [25] by state of the art methods. **a** Results of SMASH for the first tested muscle cell image. **b** Results of CELLSEGM for the first tested muscle cell image. **c** Results of SMASH for the second tested muscle cell image. **d** Results of CELLSEGM for the second tested muscle cell image

**Fig. 9:**
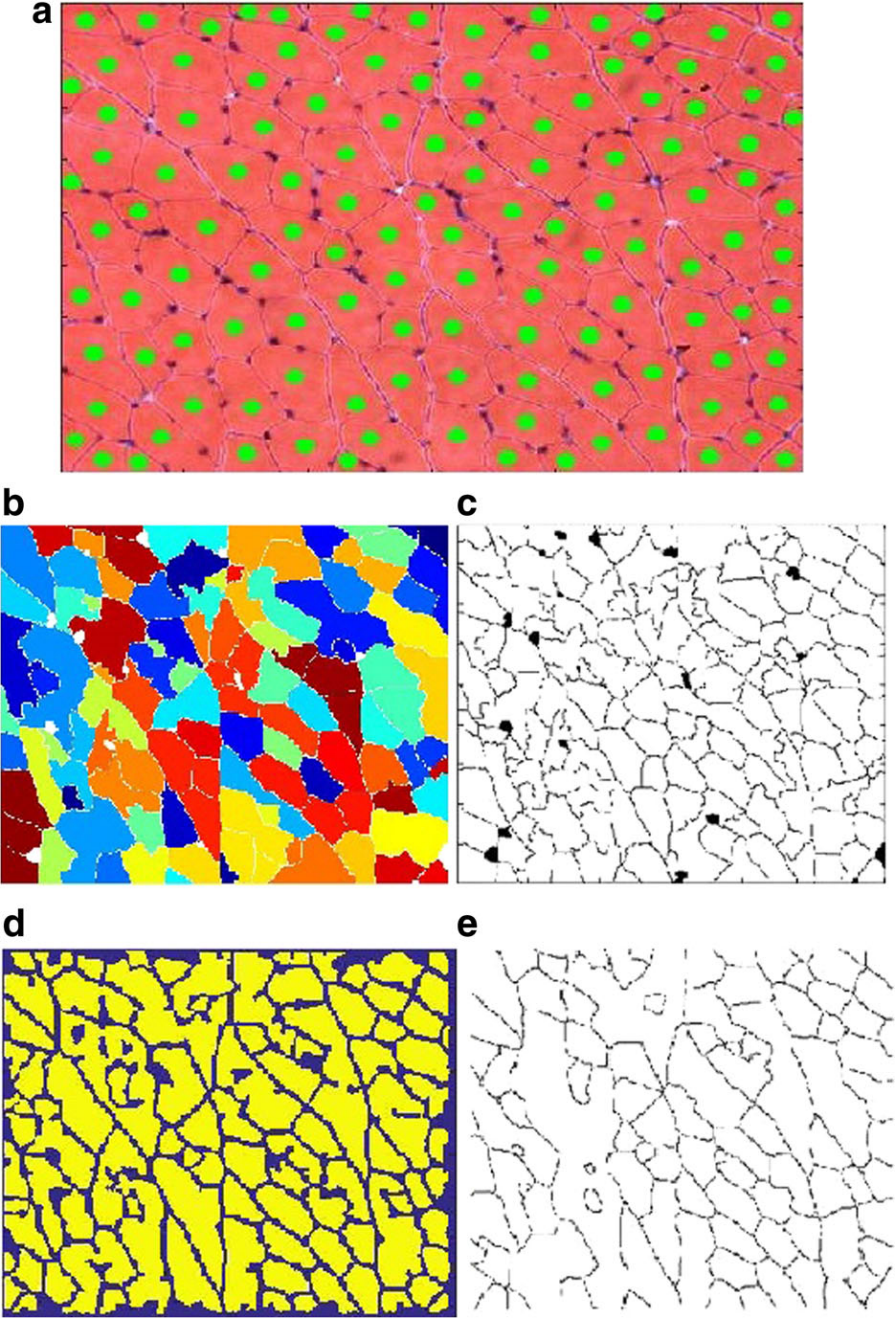
Comparison of the results of the generalized framework with state of the art methods. **a** Result of the generalized framework. **b** Intermediate result of SMASH. **c** Final result of SMASH. **d** Intermediate result of CELLSEGM. **e** Final result of CELLSEGM

**Fig. 10:**
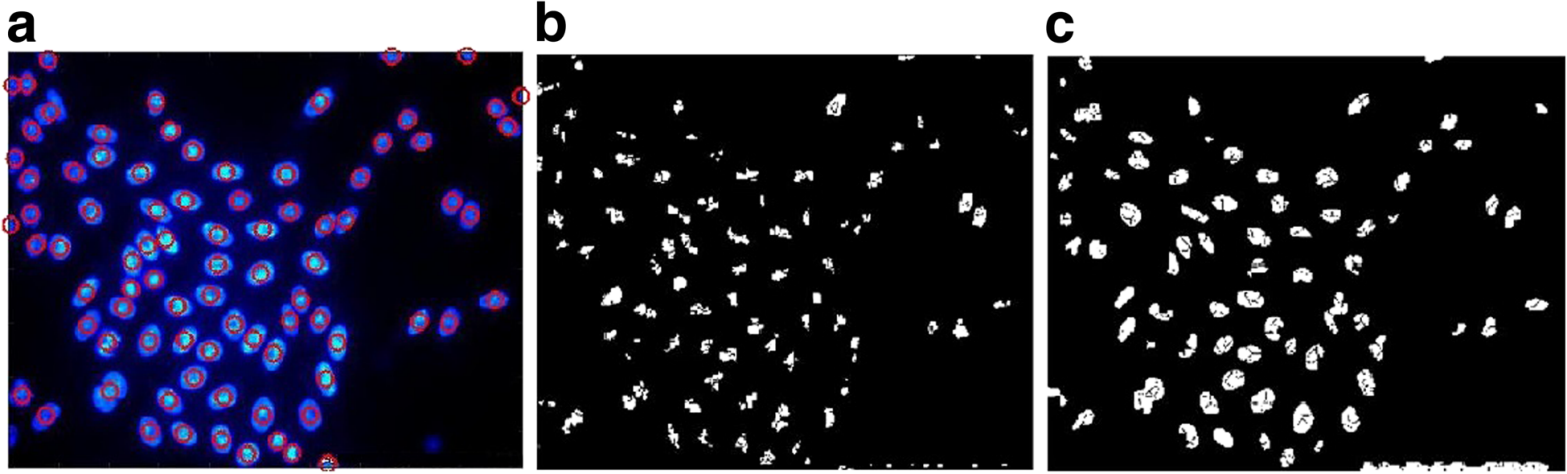
Comparison of the results of the generalized framework with state of the art methods. **a** Result of the generalized framework. **b** Results of SMASH. **c** Result of CELLSEGM

To demonstrate the advantage of the generalized framework over state of the art methods, we show the results of the two muscle cell images by the SMASH method [34] and the CELLSEGM method [35] in Fig. 8. As can be seen, the generalized framework yields significantly better results than state of the art methods [34, 35]. More comparisons are given with different types of cell images in Figs. 9 and 10. In Fig. 9, the muscle cell boundaries are much more unclear than those in Fig. 7. The generalized framework still achieves good result while state of the art methods performed significantly worse. We show the quantitative comparison with ten muscle cell images in Table 1. As can be seen, the proposed generalized framework achieves better accuracy than the two state of the art methods [34, 35] in segmenting muscle cell images. More importantly, the proposed framework is capable of segmenting other different types of cells besides the muscle cell images while the other two state of the art methods [34, 35] might not be capable. In Fig. 10, we show the results of a different type of cell image by these three methods. It is seen that only the generalized framework yielded meaningful result while SMASH and CELLSEGM failed.

**Table 1:** Quantitative comparison of the proposed approach with state of the methods [34, 35]

Methods	TP	FP	FN
SMASH [34]	84.27%	11.89%	16.08%
CELLSEGM [35]	82.69%	2.10%	17.31%
Proposed	95.28%	1.92%	4.72%

**Fig. 11:**
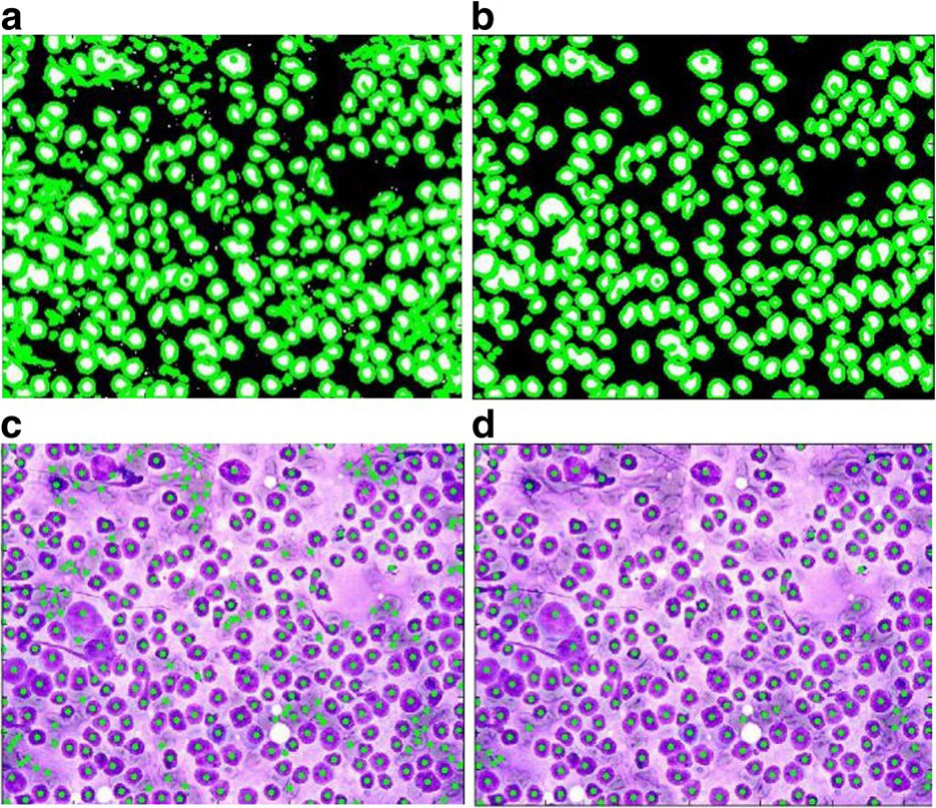
Demonstration of the effectiveness of the proposed noise blob filter. **a** The extracted boundary from the union segmentation without noise blob filtering. **b** The extracted boundary from the union segmentation after noise blob filtering. **c** The quantification result based on the extracted boundary in **a. d** The quantification result based on the extracted boundary in **b**

**Fig. 12:**
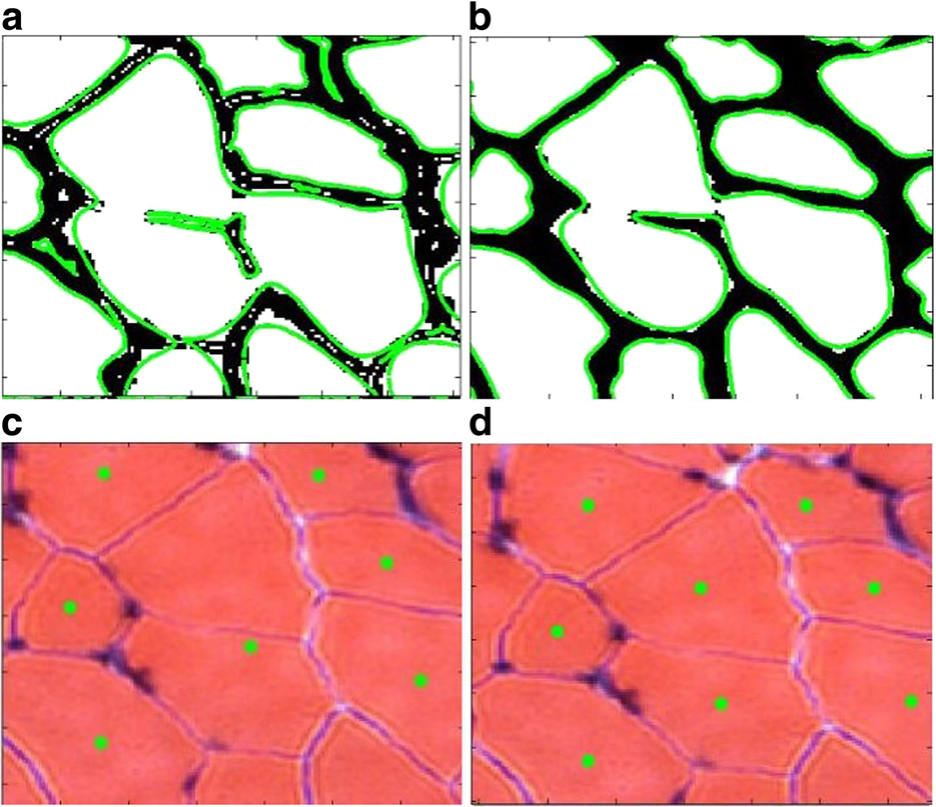
Demonstration of the effectiveness of the proposed noise blob filter with a muscle cell image. **a** The extracted boundary from the union segmentation without noise blob filtering. **b** The extracted boundary from the union segmentation after noise blob filtering. **c** The quantification result based on the extracted boundary in **a. d** The quantification result based on the extracted boundary in **b**

The effectiveness of the proposed boundary smoothing filter has been verified by the qualitative results shown in Figs. 5 and 6. Similarly, we show the effectiveness of the proposed noise blob removing filter in Figs. 11 and 12. Figure 11a shows the boundary extracted directly from the union segmentation without noise blob filtering and Fig. 11b shows the extracted boundary from the union segmentation after noise blob filtering. Figure 11c shows the final quantification results based on the extracted boundary in Fig. 11a and d shows the final quantification results based on the extracted boundary in Fig. 11b. As can be seen, the quantification accuracy based on the filtered result by the noise blob filter is significantly higher that of the result without noise blob filtering. Figure 12 shows another example of muscle cell image. The extracted boundary without and with the noise blob filter affects the final accuracy of the quantification result obviously. There is one missing quantification in Fig. 12c, which is caused by the noise blobs.

For the quantitative result, the same cell image dataset used in [24, 25] is used for validation of the generalized framework proposed in this paper. The measure for accuracy evaluation is the same as [24, 25]. The true positive (TP) is defined as that there is one and only one identified cell inside each “ground-truth” boundary; The false positive (FP) is defined as that there is more than one identified cell inside each “ground-truth” boundary. The false negative (FN) is defined as that there is none identified cell inside each “ground-truth” boundary. The comparison is shown in Table 2. As can be seen, the robustness of the generalized framework is superior to the proposed method in [24, 25].

**Table 2:** Quantitative comparison of the quantification accuracy with [24, 25]

Methods	TP	FP	FN
[24, 25]	93.4%	0.18%	6.6%
Proposed	96.8%	0.12%	3.2%

We use the 20 synthetic fluorescent cell images from the open access Broad Bioimage Benchmark Collection (BBBC) [36] for the general comparison with state-of-the-art methods. Among the referenced literatures, only [18] reports quantitative results based on the BBBC dataset. Hence, we compare the proposed method with [18] using the quantitative results in Table 3. The correct quantification rate which is denoted as TP in Table 2 is 93.5%, which is better than that of [18], 91.8%. Overall, the robustness and generality of the proposed framework is validated. We share the codes for testing the quantitative results with these 20 synthetic images in the section of *Data availability*. Since the generalized framework evolves and enhances the previous approaches [24, 25] and it inherits all their merits, more performance evaluation of the generalized framework could also be referred from the past work [24, 25].

**Table 3:** Quantitative comparison of the quantification accuracy with State of the art methods

Methods	TP	FP	FN
[18]	91.8%	NA	8.2%
Proposed	93.5%	NA	6.5%

## Discussion

The microscopy imaging technology has been developed rapidly in recent years. Accordingly, image processing techniques for automatic cell segmentation and robust quantification are becoming more and more necessary. According to our investigation, we concluded that threshold selection is the most appropriate method in this application due to its good efficiency, good resistance to noise and easy implementation. State of the art threshold selection methods [29–33] failed to select the threshold robustly for the gradient image as stated and proved in [24, 25]. As a result, the threshold selection method was evolved and utilized in [25] to segment the muscle cell images and its advantage over state of the art thresholding methods was also verified in [25]. Later, the threshold selection is improved further in [24] by adding the calibration procedure to the selection process. As a result, the threshold selection becomes flexible and could segment different types of cells robustly. In this paper, we propose a simpler and more practical calibration method to determine the parameters for the threshold selection method based on the third property of the slope difference distribution.

Only with the thresholding method to guarantee the accurate enough and complete enough segmentation, we could proceed to high level applications, e.g. boundary extraction or quantification. There are two challenging aspects for automatic and reliable quantification of cells by the proposed iterative erosion method in [24, 25]: (1), there are some noise blobs that might be identified as the cell seed by the iterative erosion method. (2), the extracted boundaries of the cell blobs are usually irregular with noise. (3), there might be holes inside the cell blobs. All these three aspects will increase the number of the false seeds produced by the iterative erosion method proposed in [24, 25]. To solve these problems, we propose a noise blob removing filter to get rid of the threadlike or small noise blobs. We propose a boundary smoothing filter to smooth the extracted boundary of the cell blobs and also eliminate small holes inside the cell blobs.

To verified the proposed methods in this paper, both qualitative and quantitative experiments are conducted. As it turned out, the proposed framework is more versatile than other state of the art methods due to the fact that it utilizes the characteristics of the adjacent cells and the general property of the cell images: the global intensity distribution and the local gradient. The frequently occurring overlapping characteristics of the adjacent cells could be dealt effectively by the iterative erosion method. The intensity image and the gradient image could be segmented effectively by the proposed segmentation method. The segmentation method is able to segment different kinds of images and their formed gradient images more accurately because of the introduced calibration process.

## Conclusion

In this paper, we propose a generalized framework for automatically segmenting and quantifying different types of cells. To simplify the calibration process for the threshold selection, we proposed a practical calibration method. To improve the quantification accuracy over the past research, we proposed a noise blob filtering method and a boundary smoothing filtering method in this paper. Experimental results verified their effectiveness. As a generalized tool for automatic segmentation and quantification of different kinds of cells, it possible for the proposed framework to benefit a lot of automated microscopy applications in the future.

## Additional file

Additional file 1: Source codes of the proposed framework with test images. (ZIP 31244 kb)

## Abbreviations

DFT: Discrete Fourier transformation
FIR: Finite impulse response
IIR: Infinite impulse response
