# Supplementary material for: Generalizing cell segmentation and quantification
Source: BMC Bioinformatics. 2017 Mar 23;18:189. doi: 10.1186/s12859-017-1604-1 (PMC5364575; doi:10.1186/s12859-017-1604-1)
Supplement: Additional file 1: — Source codes of the proposed framework with test images. (ZIP 31244 kb) [file 12859_2017_1604_MOESM1_ESM.zip › Generalizing_Codes/readme.pdf]

The provided contents include: (1), the core functions for the generalized framework; (2), four demonstration cases; (3) two quantitative evaluations based on the images from BBBC dataset.

(1), The core functions of the generalized framework include:
zhenzhou_threshold_selection_updated.m
zhenzhou_threshold_selection.m
zhenzhou_iterative_erosion.m
zhenzhou_shape_filtering.m
zhenzhou_clutter_filter.m
gradient_image.m
moving_average_filter.m

(2), four demonstration cases include:
DemoCase1.m
DemoCase2.m
DemoCase3.m
DemoCase4.m
case1.jpg
case2.tif
case3.jpg
t4.jpg

(3) two quantitative evaluations based on the images from BBBC dataset.
QuantitativeEvaluationBBBC04.m
QuantitativeEvaluationBBBC30.m
Images from BBBC dataset
