# Supplementary figures and images for: Generalizing cell segmentation and quantification
Source: BMC Bioinformatics. 2017 Mar 23;18:189. doi: 10.1186/s12859-017-1604-1 (PMC5364575; doi:10.1186/s12859-017-1604-1)

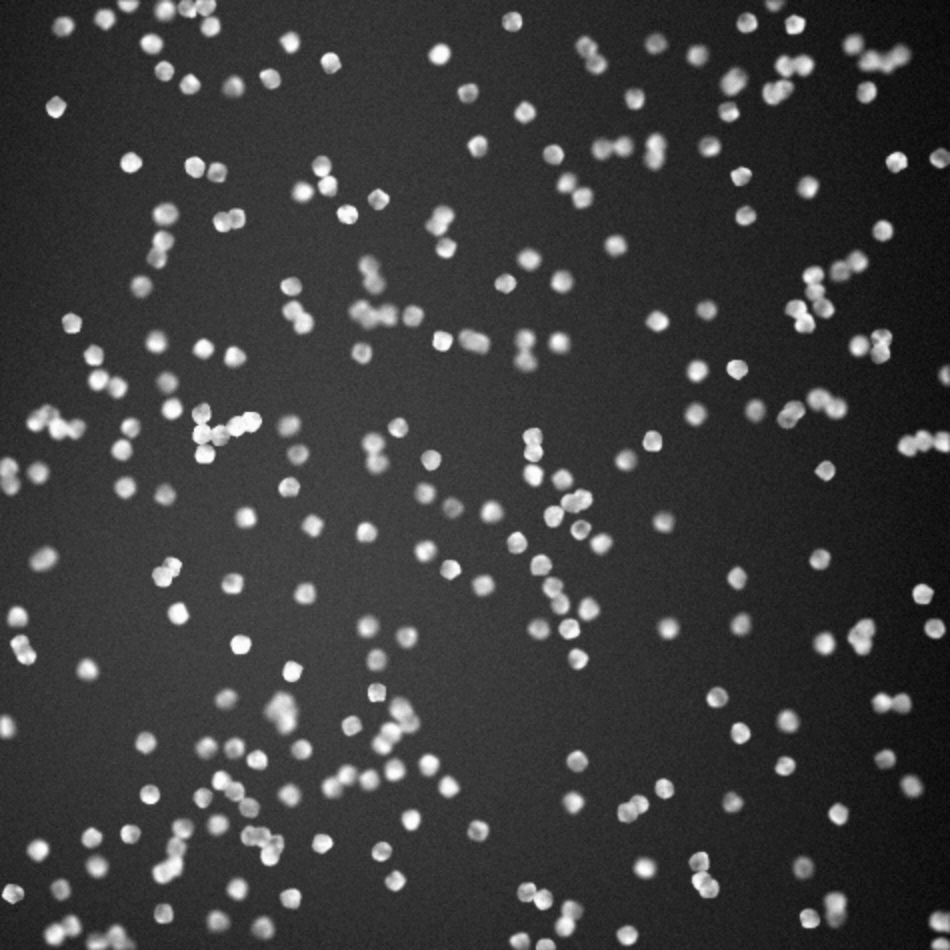

Supplement: Additional file 1: — Source codes of the proposed framework with test images. (ZIP 31244 kb) [file 12859_2017_1604_MOESM1_ESM.zip › Generalizing_Codes/10GRAY.tif]

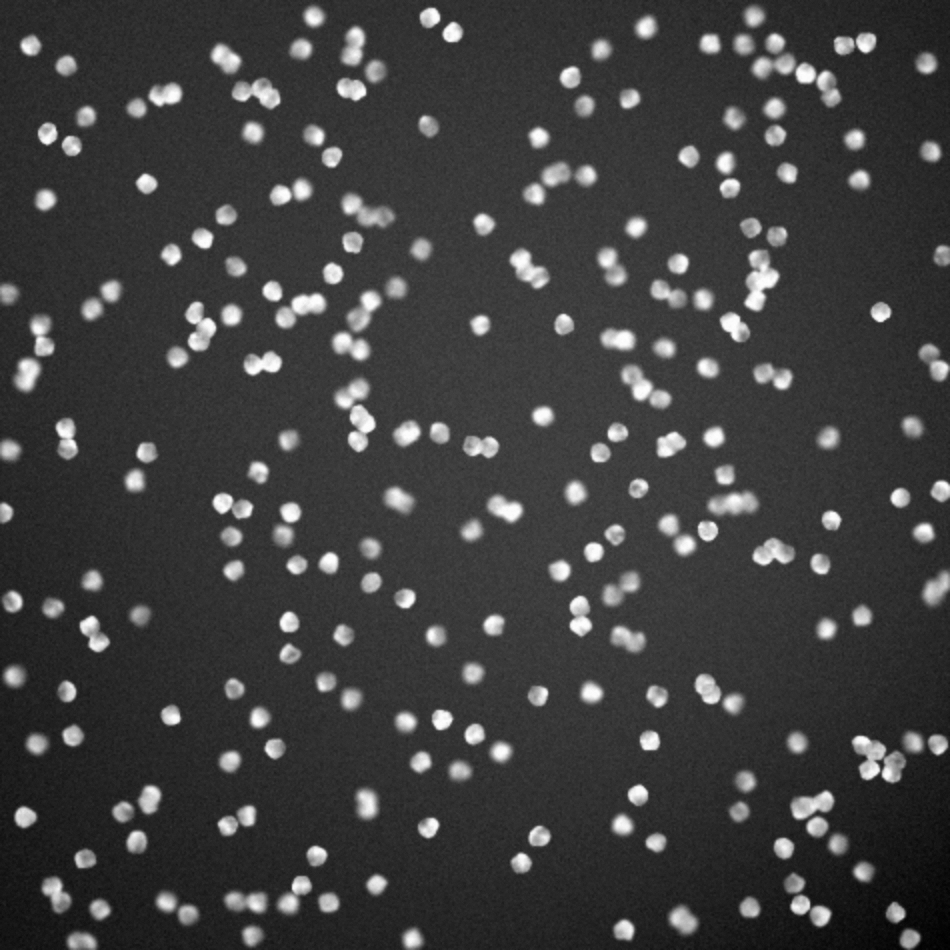

Supplement: Additional file 1: — Source codes of the proposed framework with test images. (ZIP 31244 kb) [file 12859_2017_1604_MOESM1_ESM.zip › Generalizing_Codes/11GRAY.tif]

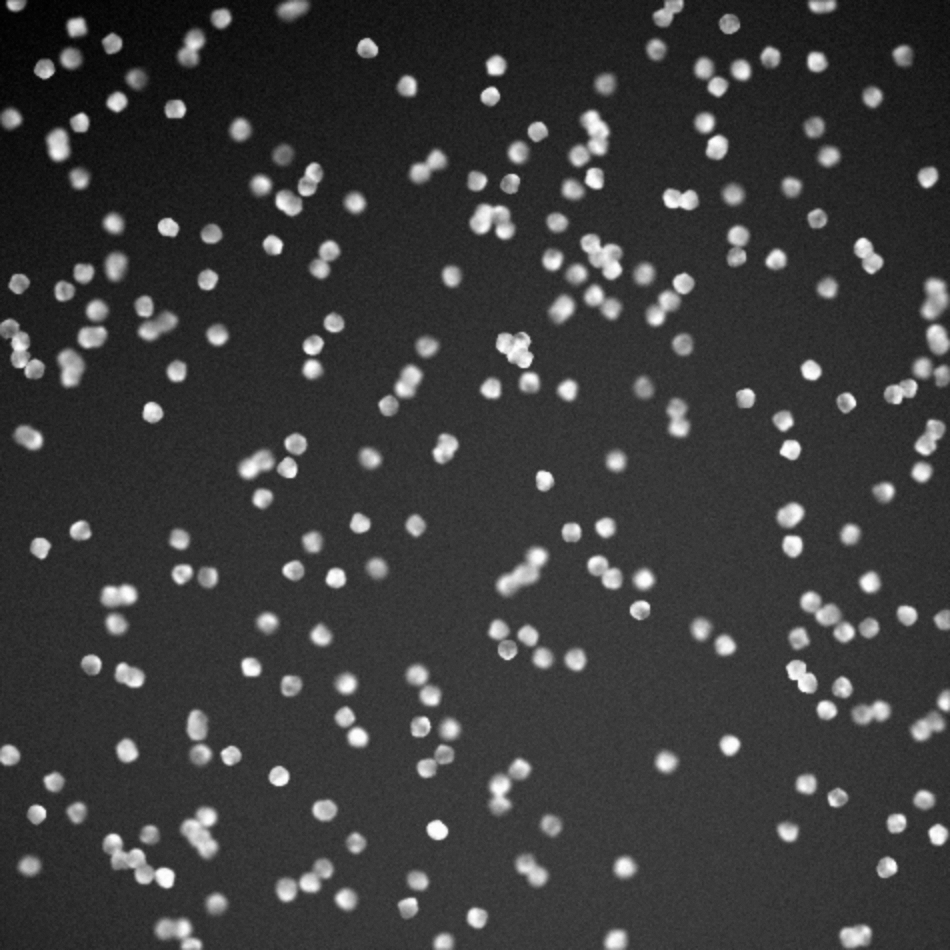

Supplement: Additional file 1: — Source codes of the proposed framework with test images. (ZIP 31244 kb) [file 12859_2017_1604_MOESM1_ESM.zip › Generalizing_Codes/12GRAY.tif]

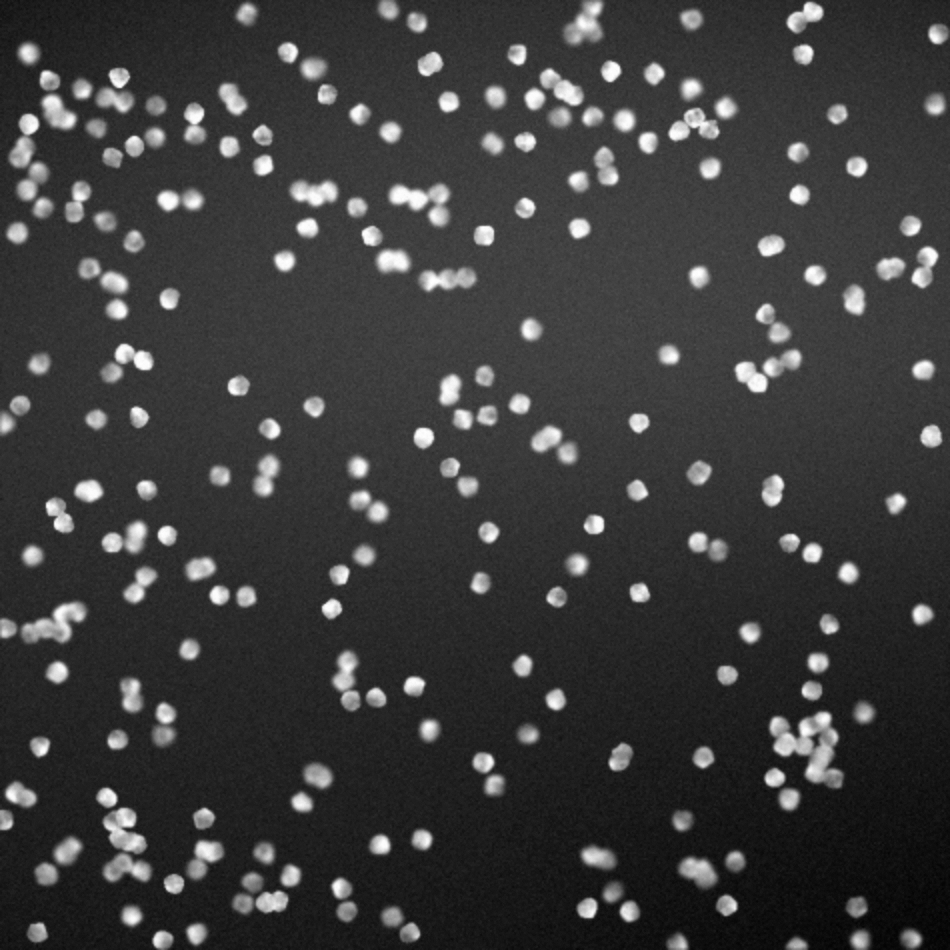

Supplement: Additional file 1: — Source codes of the proposed framework with test images. (ZIP 31244 kb) [file 12859_2017_1604_MOESM1_ESM.zip › Generalizing_Codes/13GRAY.tif]

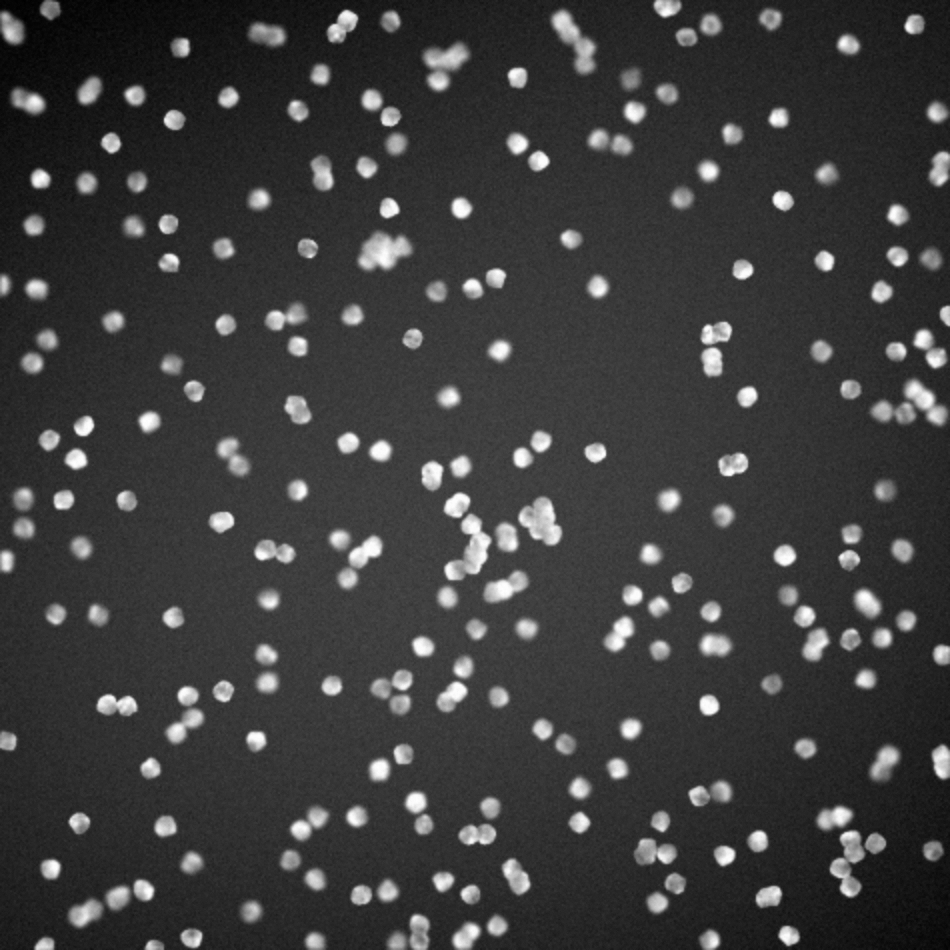

Supplement: Additional file 1: — Source codes of the proposed framework with test images. (ZIP 31244 kb) [file 12859_2017_1604_MOESM1_ESM.zip › Generalizing_Codes/14GRAY.tif]

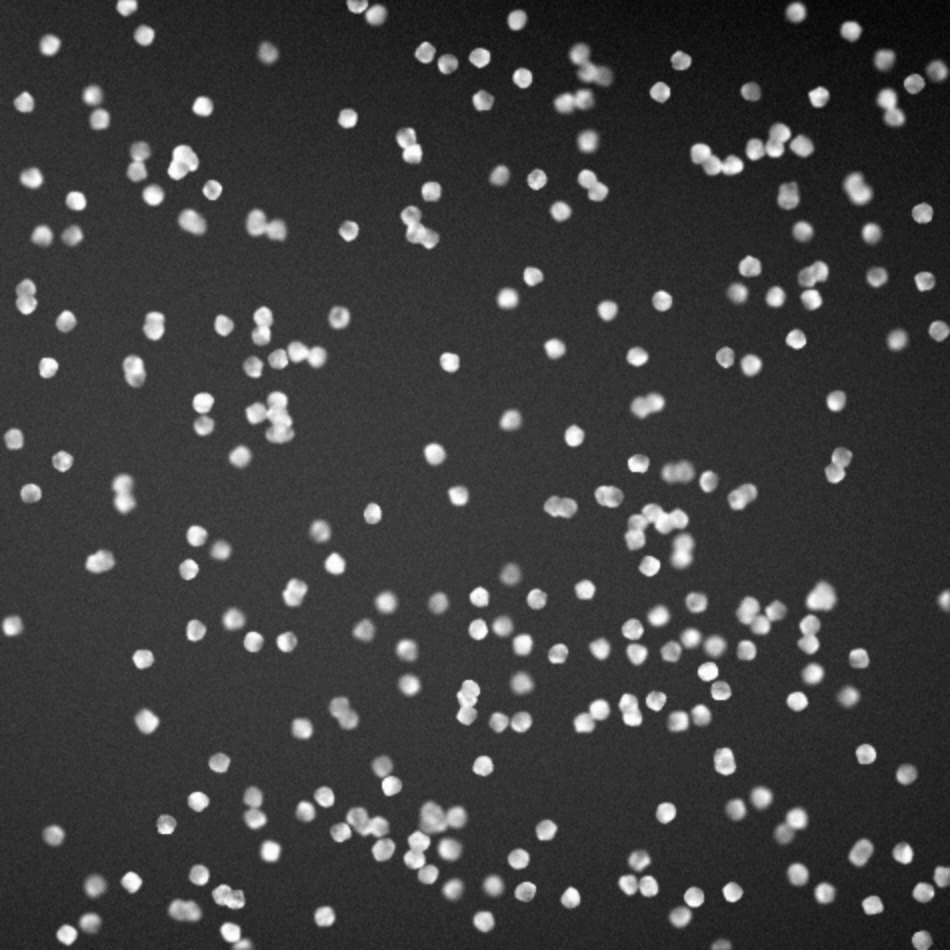

Supplement: Additional file 1: — Source codes of the proposed framework with test images. (ZIP 31244 kb) [file 12859_2017_1604_MOESM1_ESM.zip › Generalizing_Codes/15GRAY.tif]

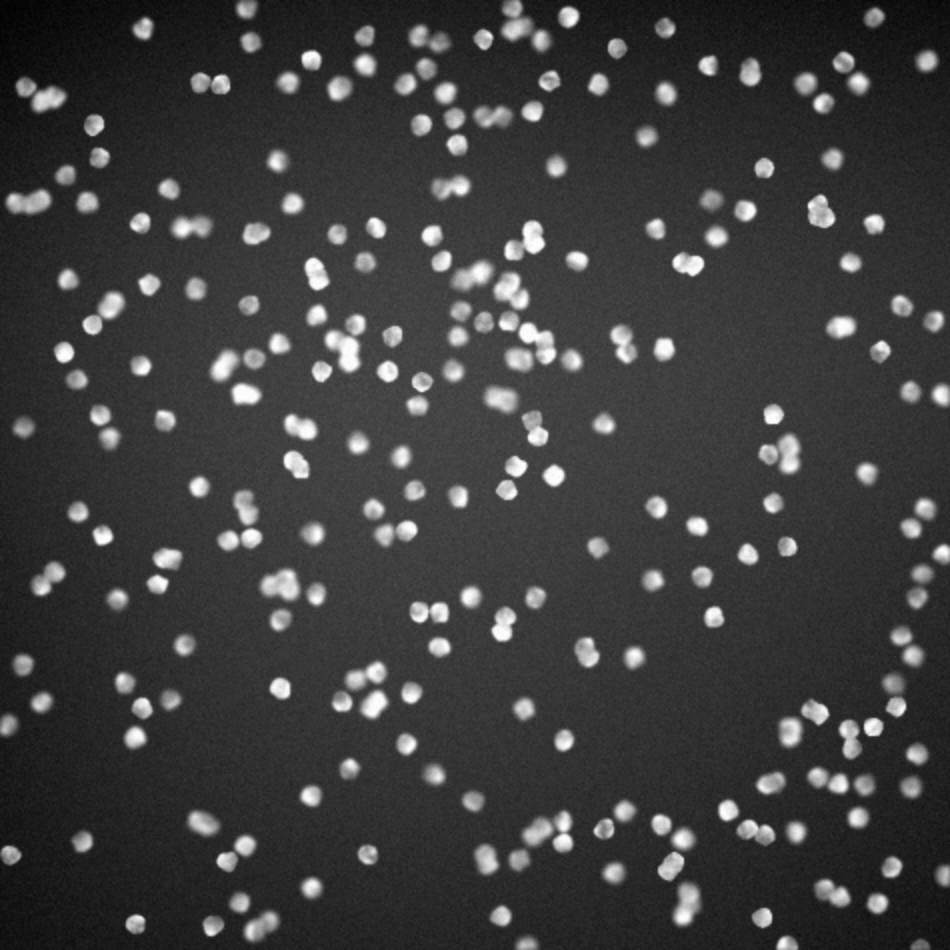

Supplement: Additional file 1: — Source codes of the proposed framework with test images. (ZIP 31244 kb) [file 12859_2017_1604_MOESM1_ESM.zip › Generalizing_Codes/16GRAY.tif]

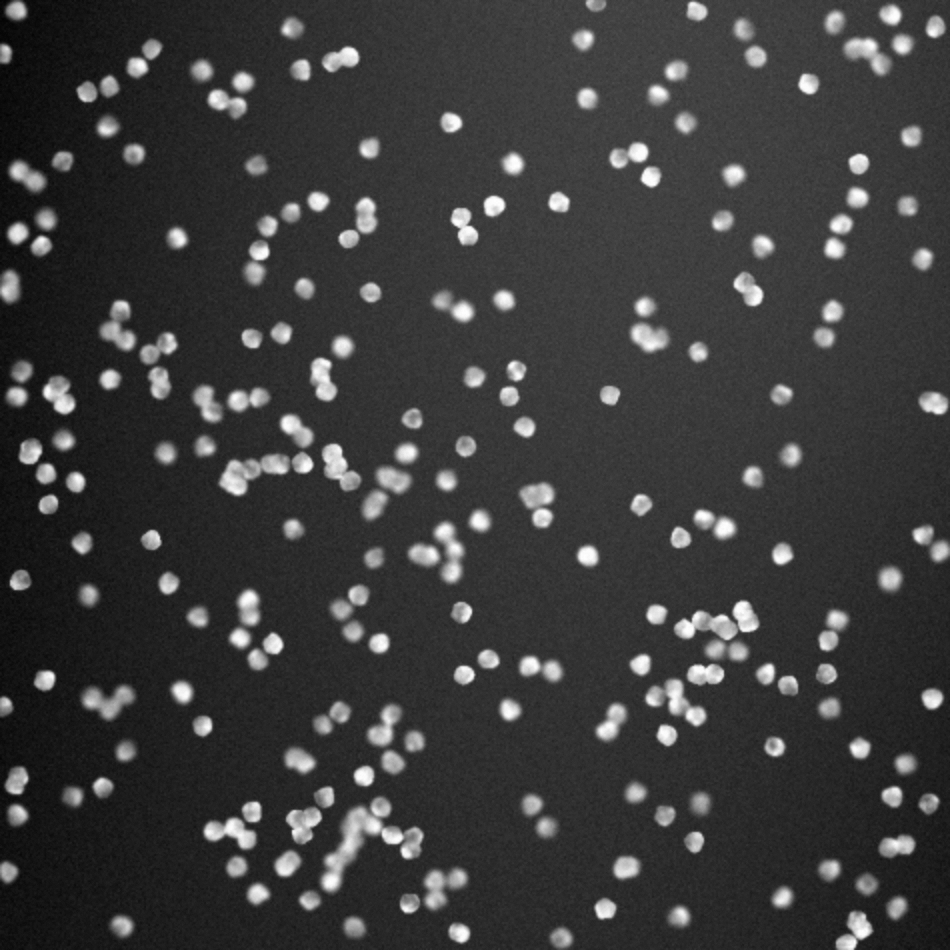

Supplement: Additional file 1: — Source codes of the proposed framework with test images. (ZIP 31244 kb) [file 12859_2017_1604_MOESM1_ESM.zip › Generalizing_Codes/17GRAY.tif]

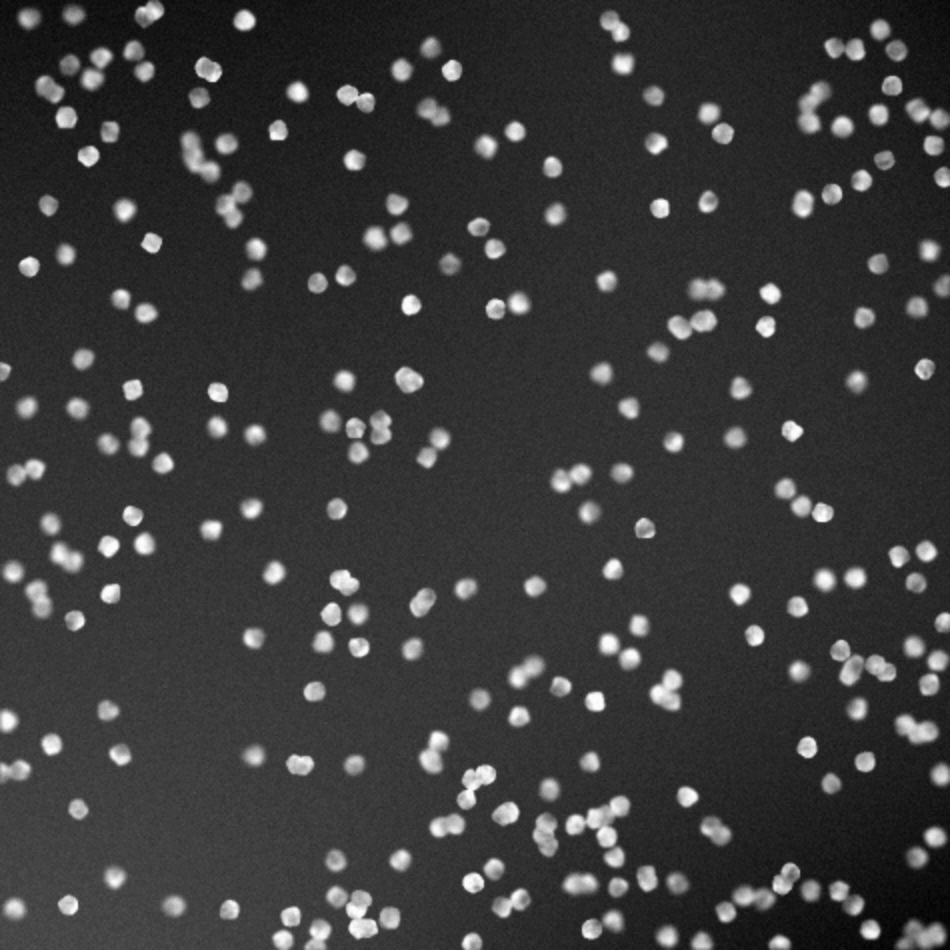

Supplement: Additional file 1: — Source codes of the proposed framework with test images. (ZIP 31244 kb) [file 12859_2017_1604_MOESM1_ESM.zip › Generalizing_Codes/18GRAY.tif]

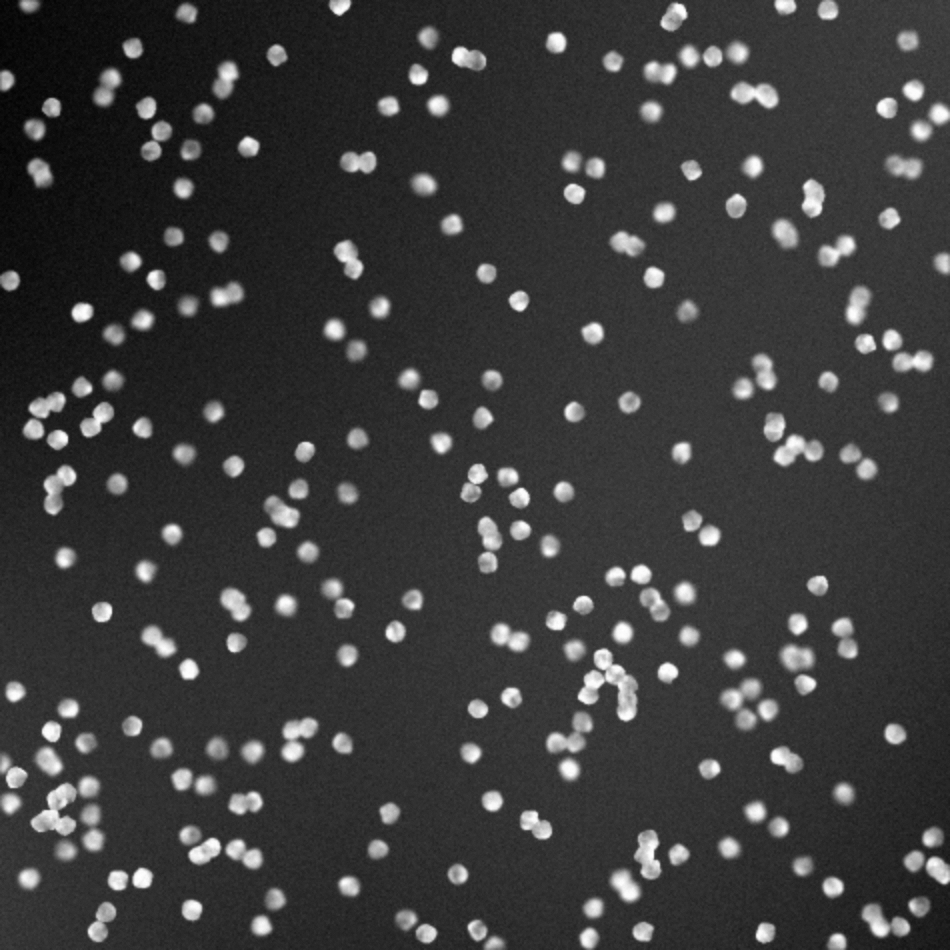

Supplement: Additional file 1: — Source codes of the proposed framework with test images. (ZIP 31244 kb) [file 12859_2017_1604_MOESM1_ESM.zip › Generalizing_Codes/19GRAY.tif]

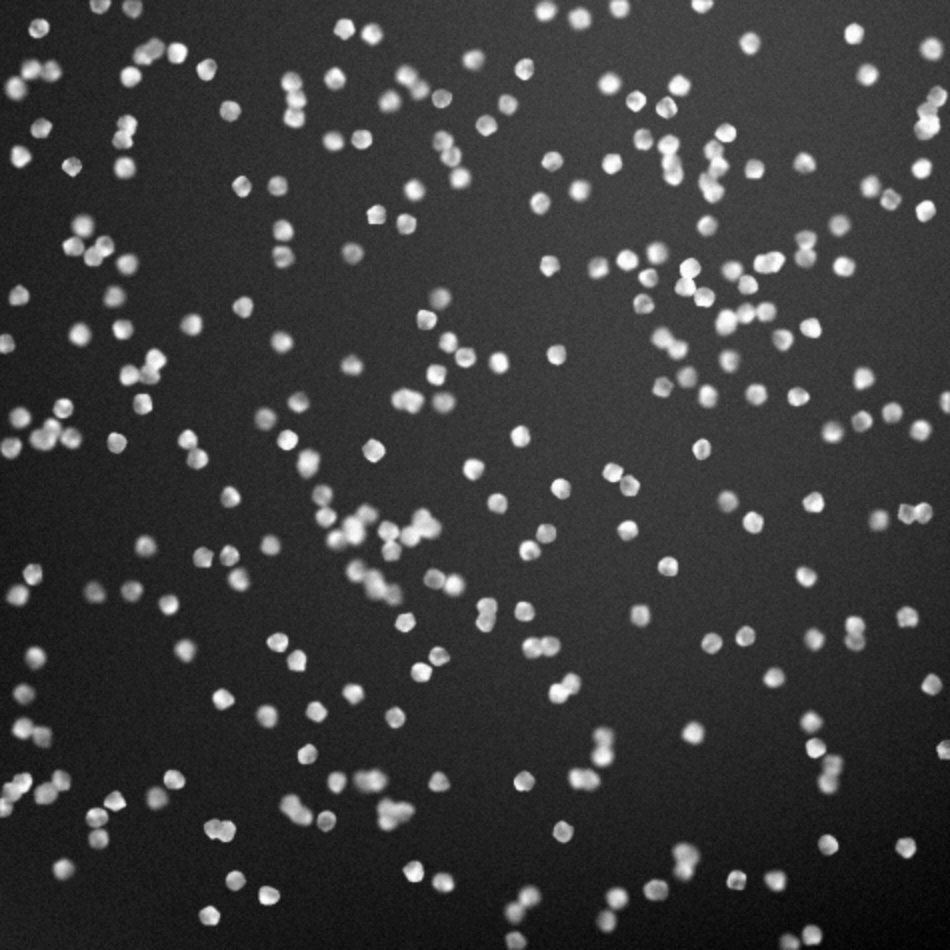

Supplement: Additional file 1: — Source codes of the proposed framework with test images. (ZIP 31244 kb) [file 12859_2017_1604_MOESM1_ESM.zip › Generalizing_Codes/1GRAY.tif]

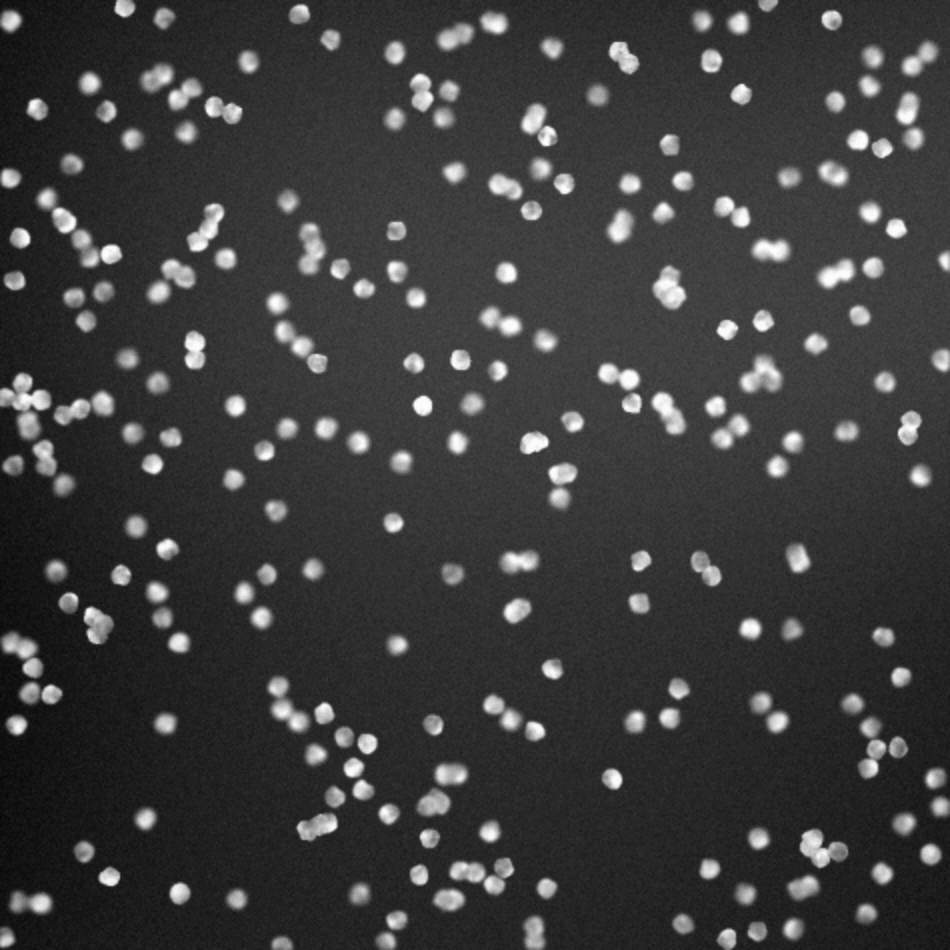

Supplement: Additional file 1: — Source codes of the proposed framework with test images. (ZIP 31244 kb) [file 12859_2017_1604_MOESM1_ESM.zip › Generalizing_Codes/20GRAY.tif]

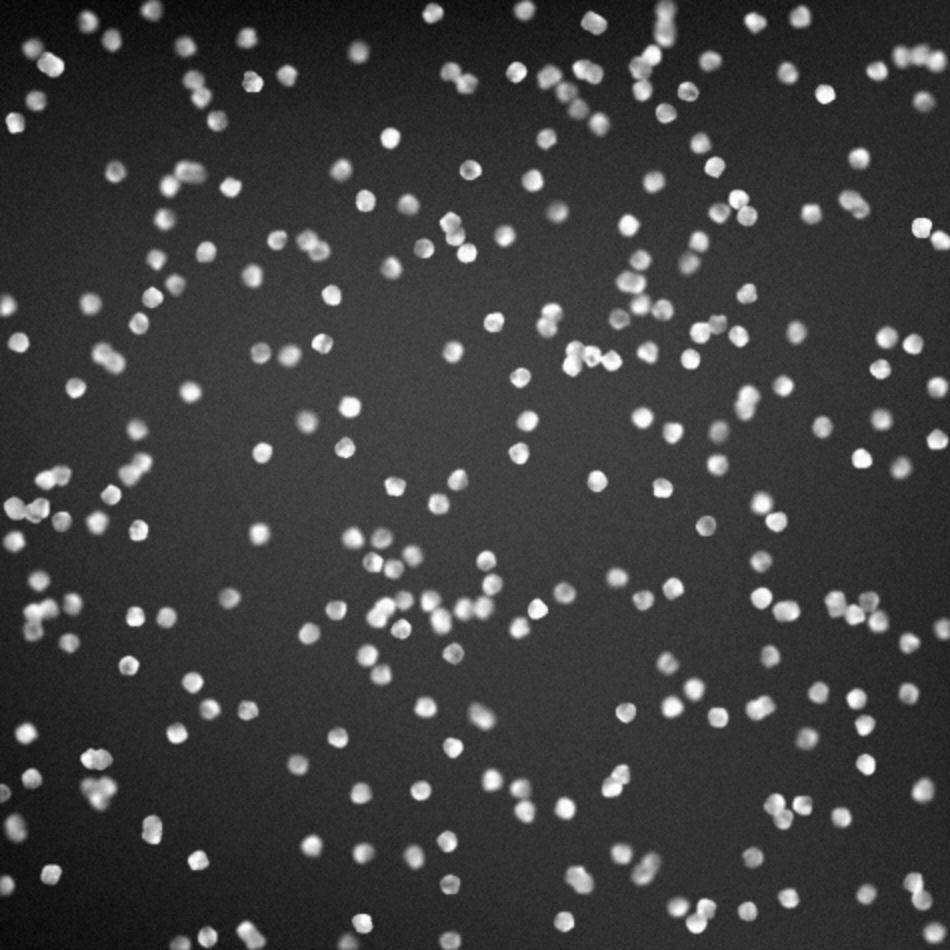

Supplement: Additional file 1: — Source codes of the proposed framework with test images. (ZIP 31244 kb) [file 12859_2017_1604_MOESM1_ESM.zip › Generalizing_Codes/2GRAY.tif]

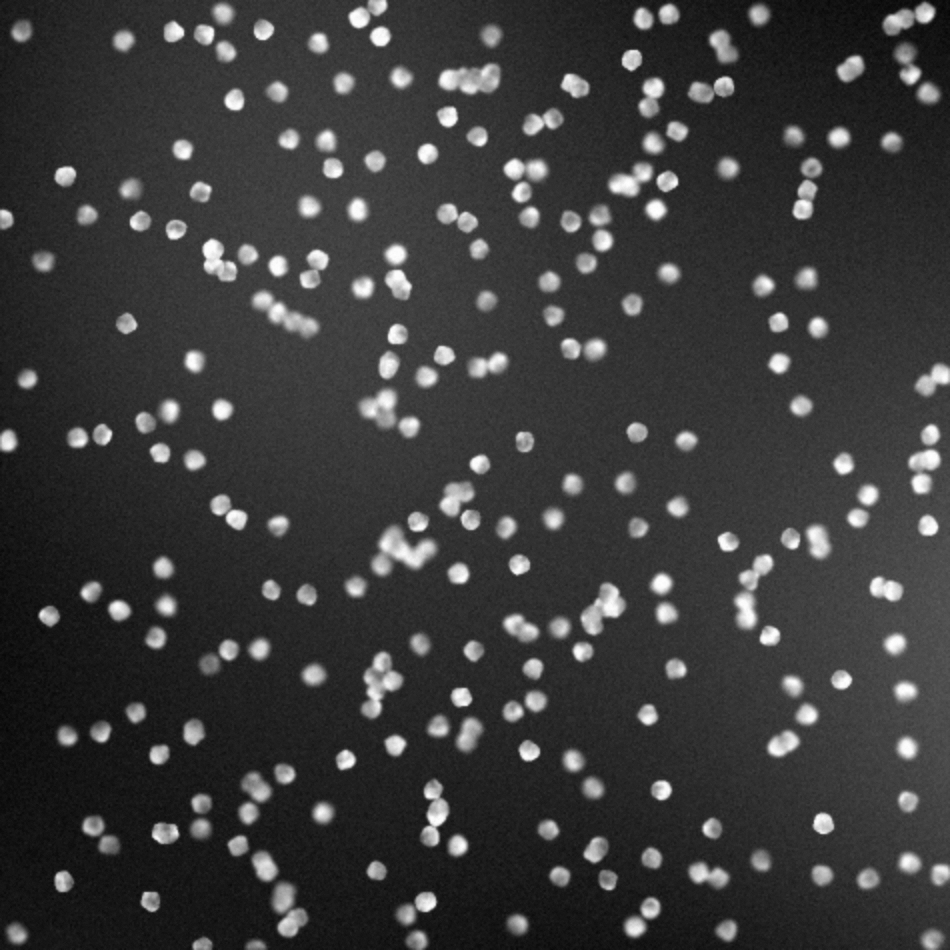

Supplement: Additional file 1: — Source codes of the proposed framework with test images. (ZIP 31244 kb) [file 12859_2017_1604_MOESM1_ESM.zip › Generalizing_Codes/3GRAY.tif]

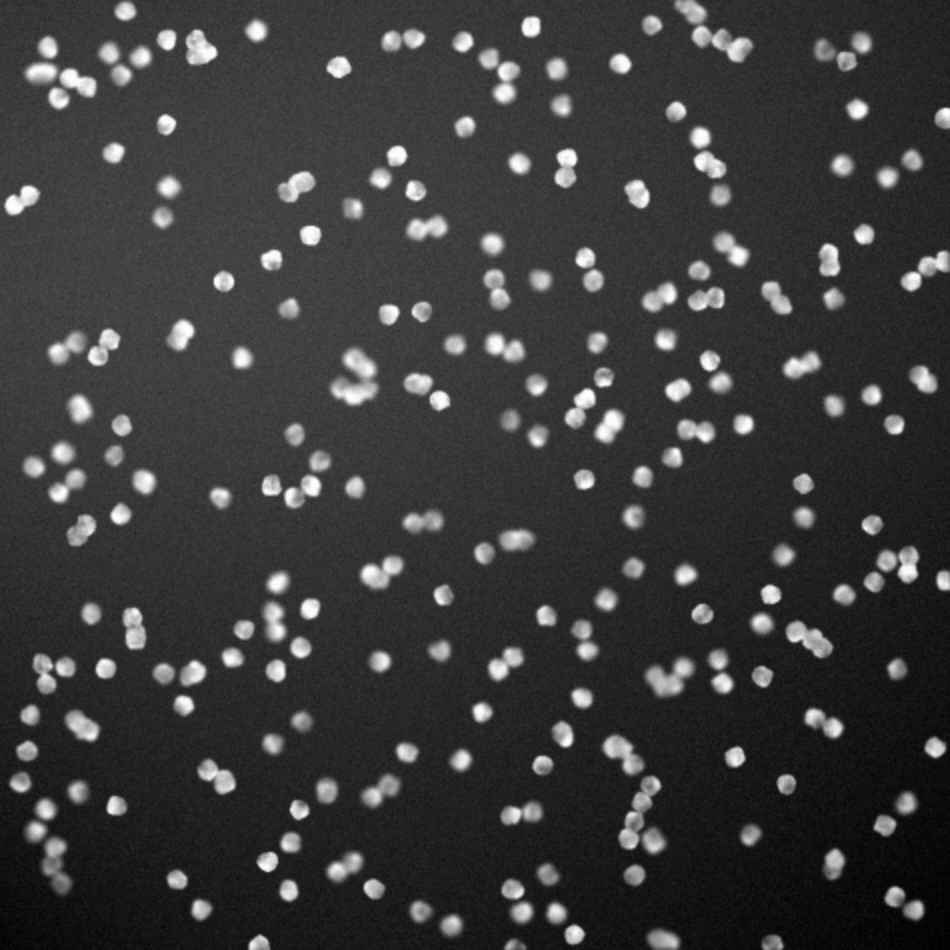

Supplement: Additional file 1: — Source codes of the proposed framework with test images. (ZIP 31244 kb) [file 12859_2017_1604_MOESM1_ESM.zip › Generalizing_Codes/4GRAY.tif]

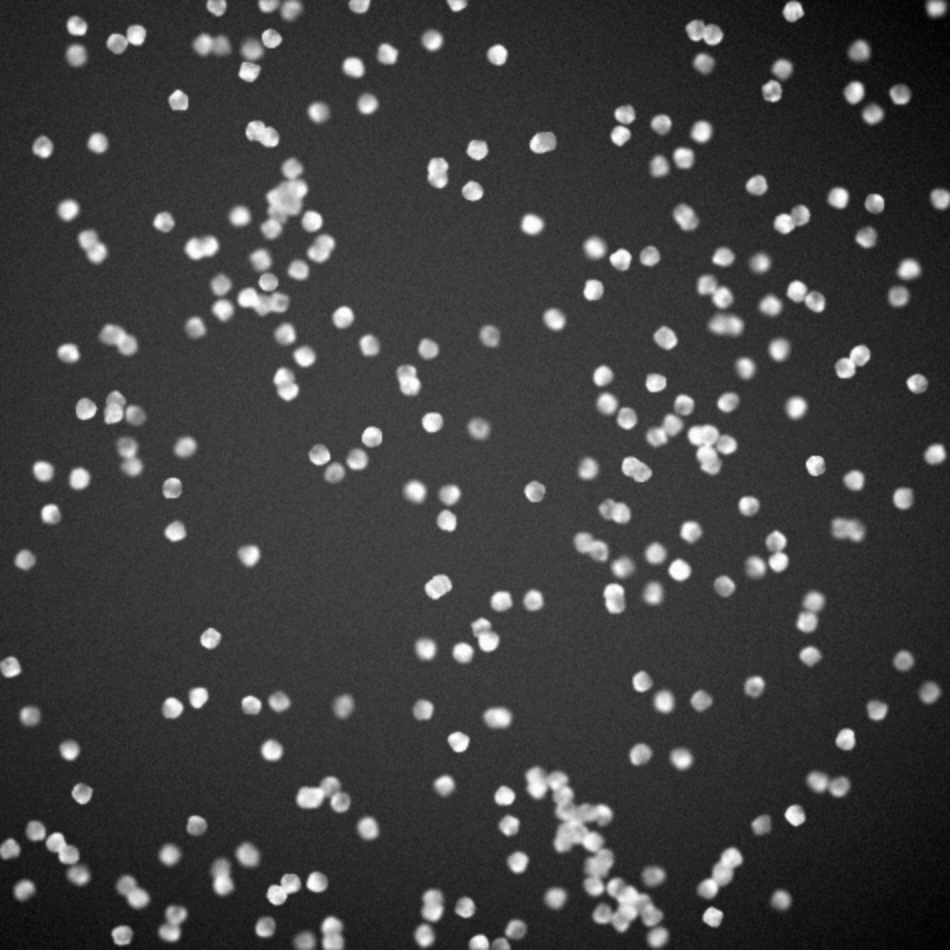

Supplement: Additional file 1: — Source codes of the proposed framework with test images. (ZIP 31244 kb) [file 12859_2017_1604_MOESM1_ESM.zip › Generalizing_Codes/5GRAY.tif]

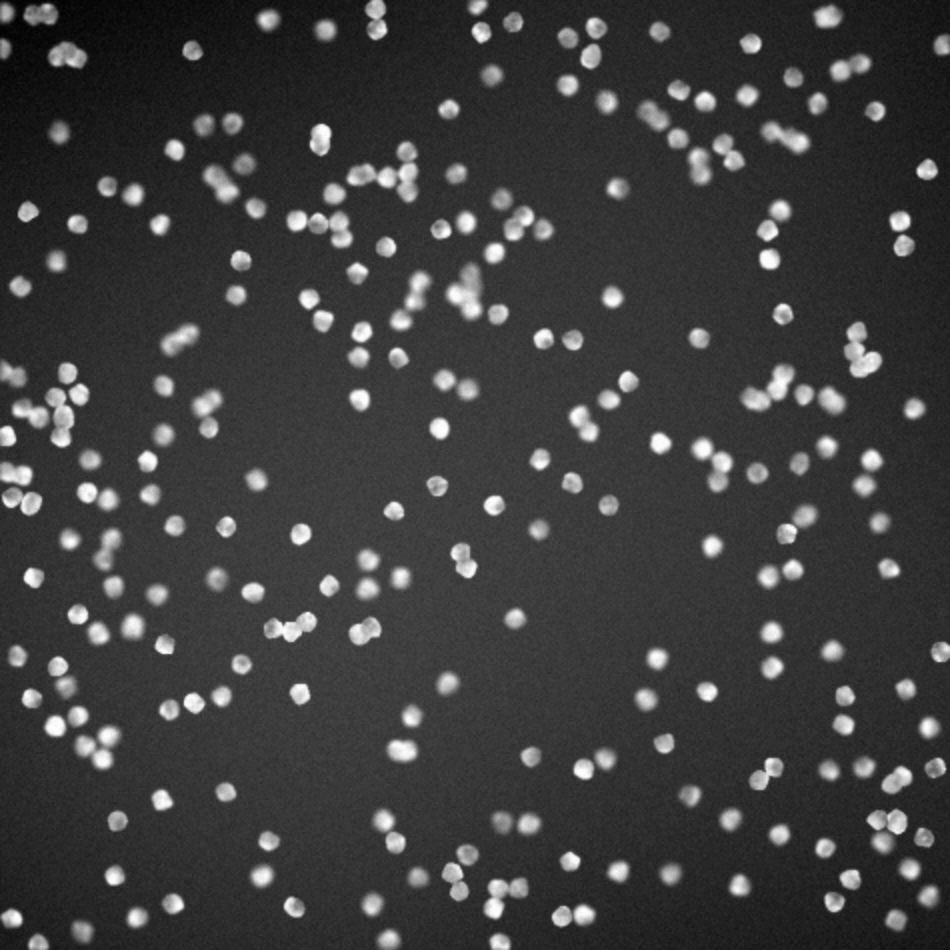

Supplement: Additional file 1: — Source codes of the proposed framework with test images. (ZIP 31244 kb) [file 12859_2017_1604_MOESM1_ESM.zip › Generalizing_Codes/6GRAY.tif]

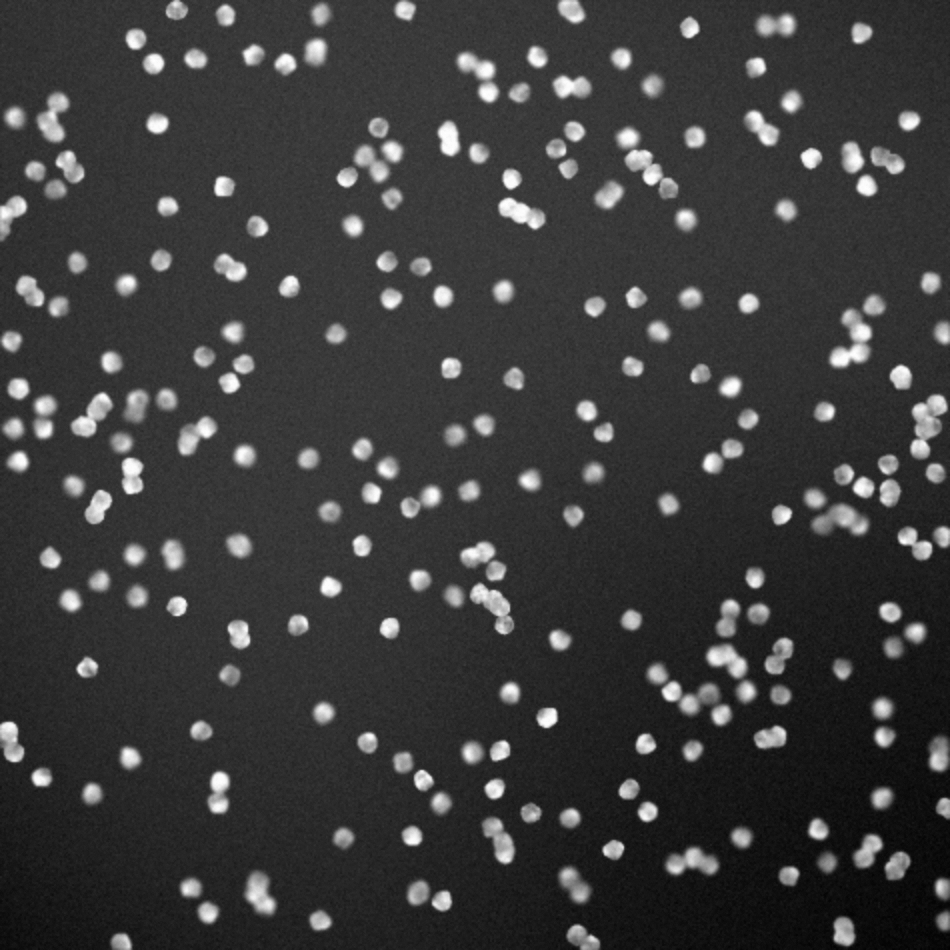

Supplement: Additional file 1: — Source codes of the proposed framework with test images. (ZIP 31244 kb) [file 12859_2017_1604_MOESM1_ESM.zip › Generalizing_Codes/7GRAY.tif]

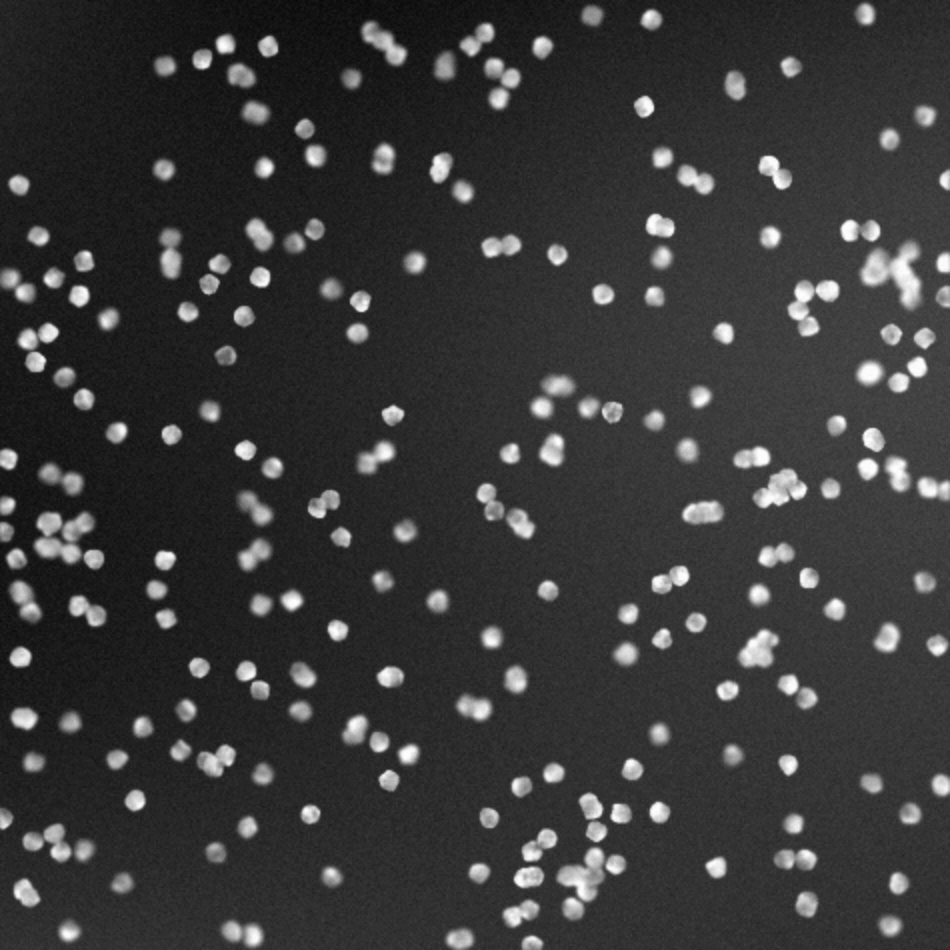

Supplement: Additional file 1: — Source codes of the proposed framework with test images. (ZIP 31244 kb) [file 12859_2017_1604_MOESM1_ESM.zip › Generalizing_Codes/8GRAY.tif]

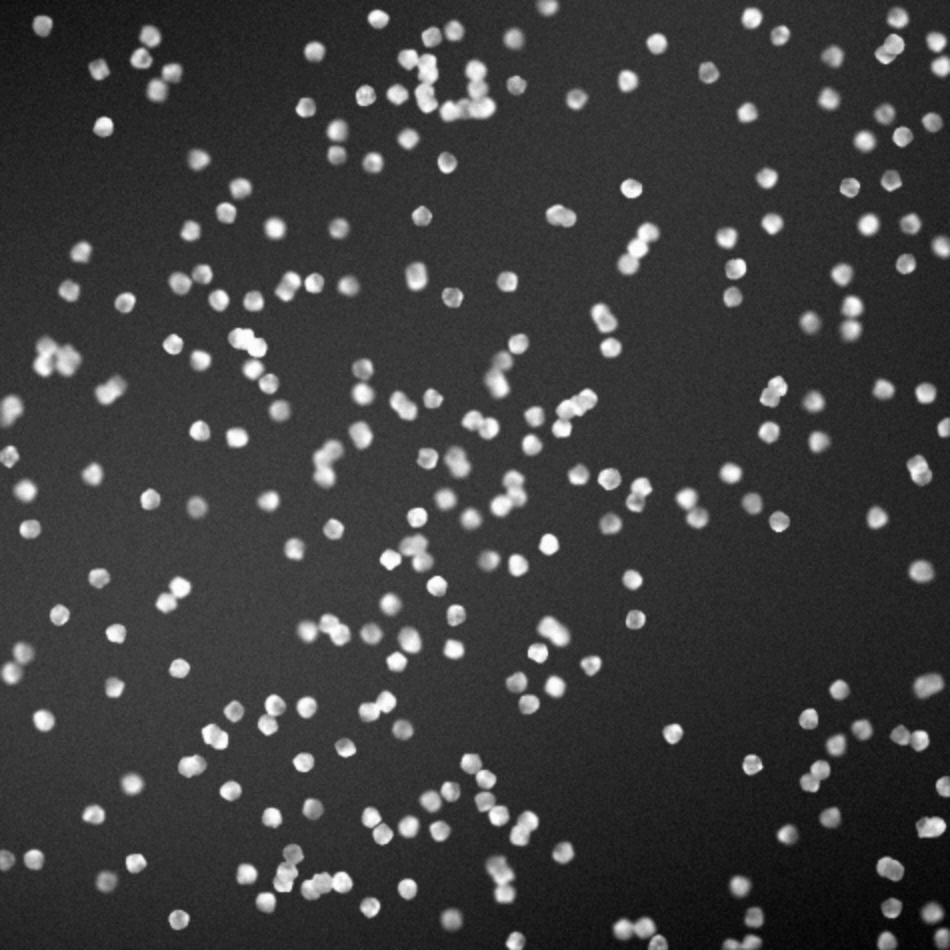

Supplement: Additional file 1: — Source codes of the proposed framework with test images. (ZIP 31244 kb) [file 12859_2017_1604_MOESM1_ESM.zip › Generalizing_Codes/9GRAY.tif]

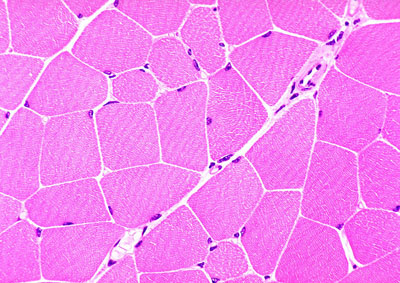

Supplement: Additional file 1: — Source codes of the proposed framework with test images. (ZIP 31244 kb) [file 12859_2017_1604_MOESM1_ESM.zip › Generalizing_Codes/case1.jpg]

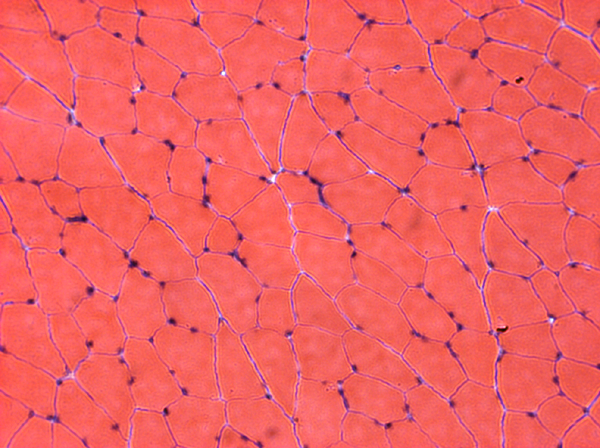

Supplement: Additional file 1: — Source codes of the proposed framework with test images. (ZIP 31244 kb) [file 12859_2017_1604_MOESM1_ESM.zip › Generalizing_Codes/case2.tif]

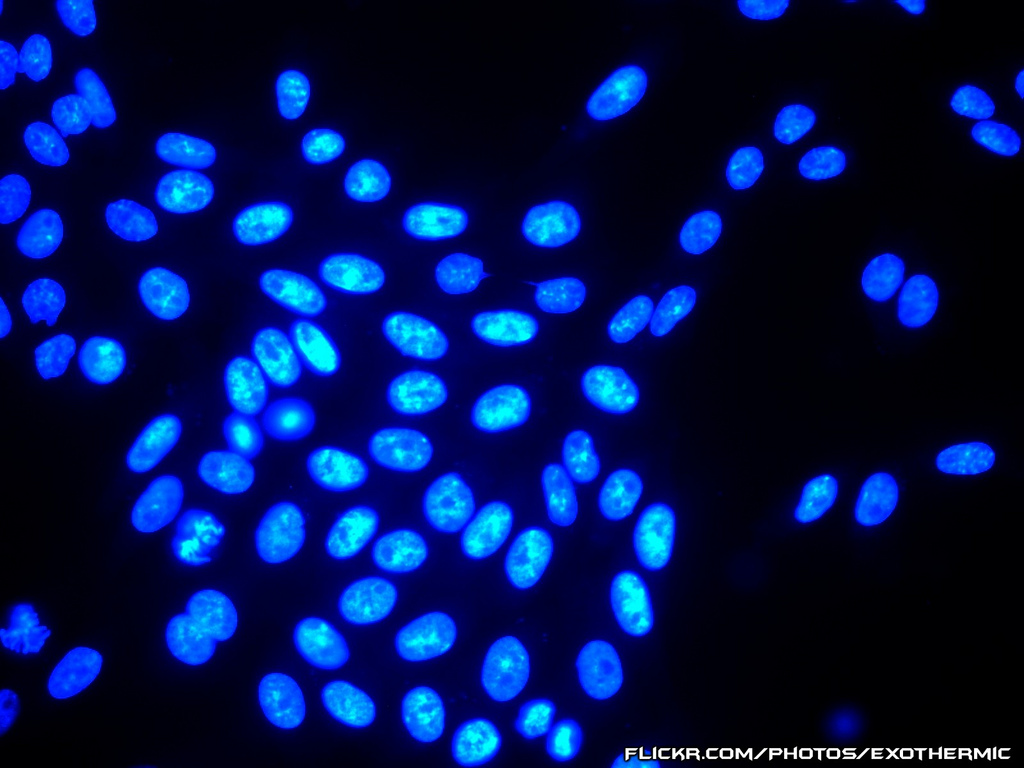

Supplement: Additional file 1: — Source codes of the proposed framework with test images. (ZIP 31244 kb) [file 12859_2017_1604_MOESM1_ESM.zip › Generalizing_Codes/case3.jpg]

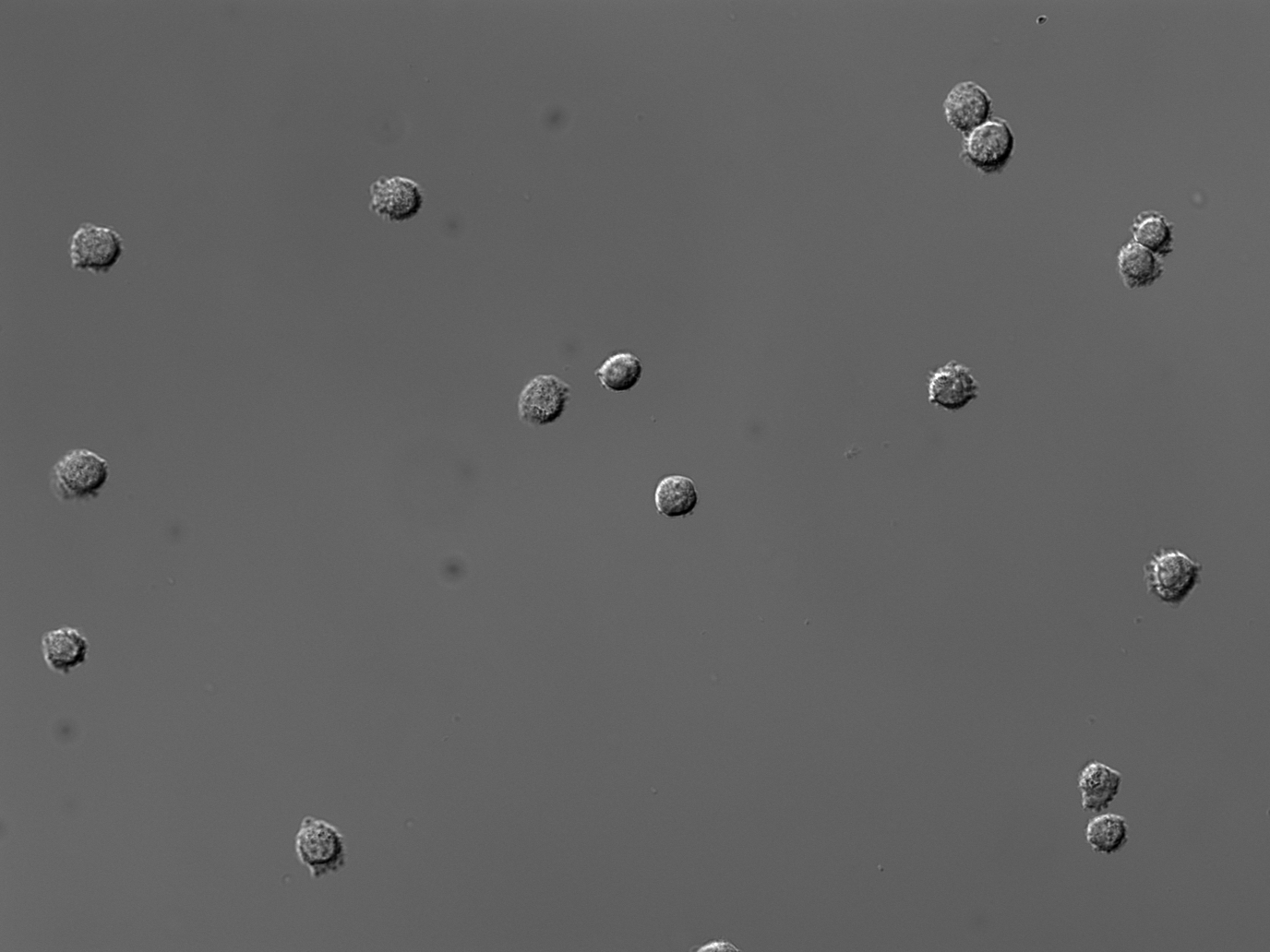

Supplement: Additional file 1: — Source codes of the proposed framework with test images. (ZIP 31244 kb) [file 12859_2017_1604_MOESM1_ESM.zip › Generalizing_Codes/cho01.png]

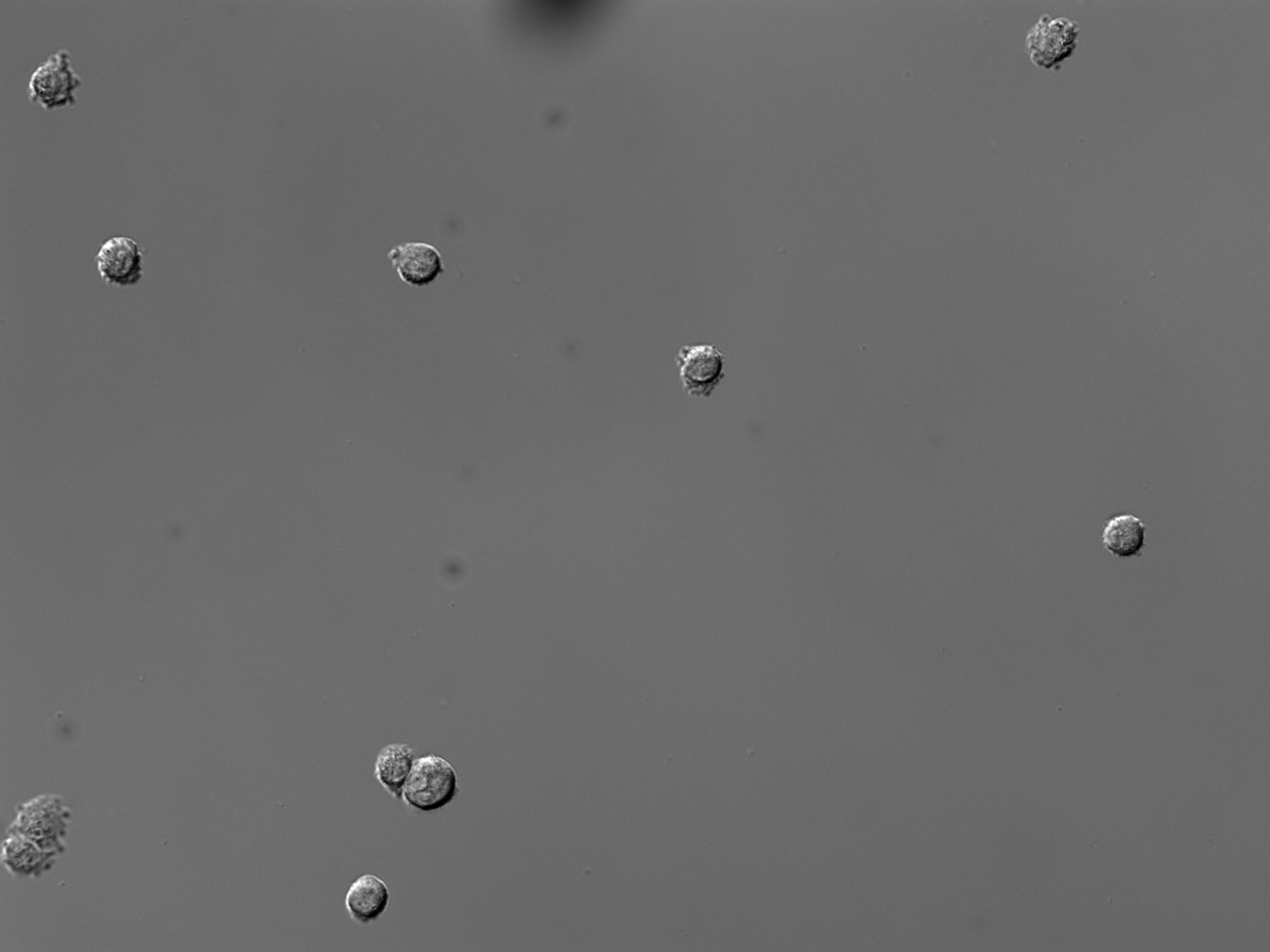

Supplement: Additional file 1: — Source codes of the proposed framework with test images. (ZIP 31244 kb) [file 12859_2017_1604_MOESM1_ESM.zip › Generalizing_Codes/cho02.png]

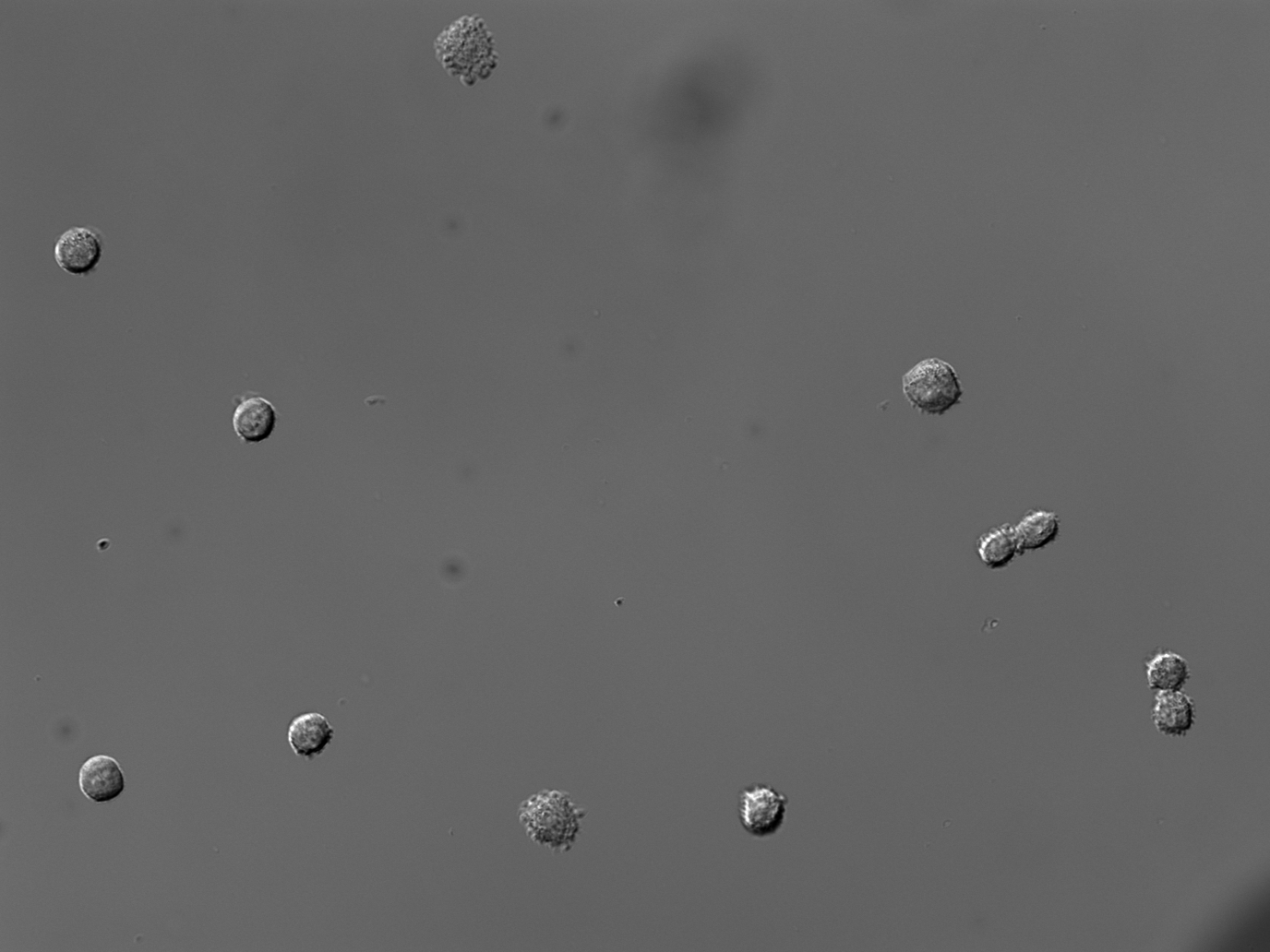

Supplement: Additional file 1: — Source codes of the proposed framework with test images. (ZIP 31244 kb) [file 12859_2017_1604_MOESM1_ESM.zip › Generalizing_Codes/cho03.png]

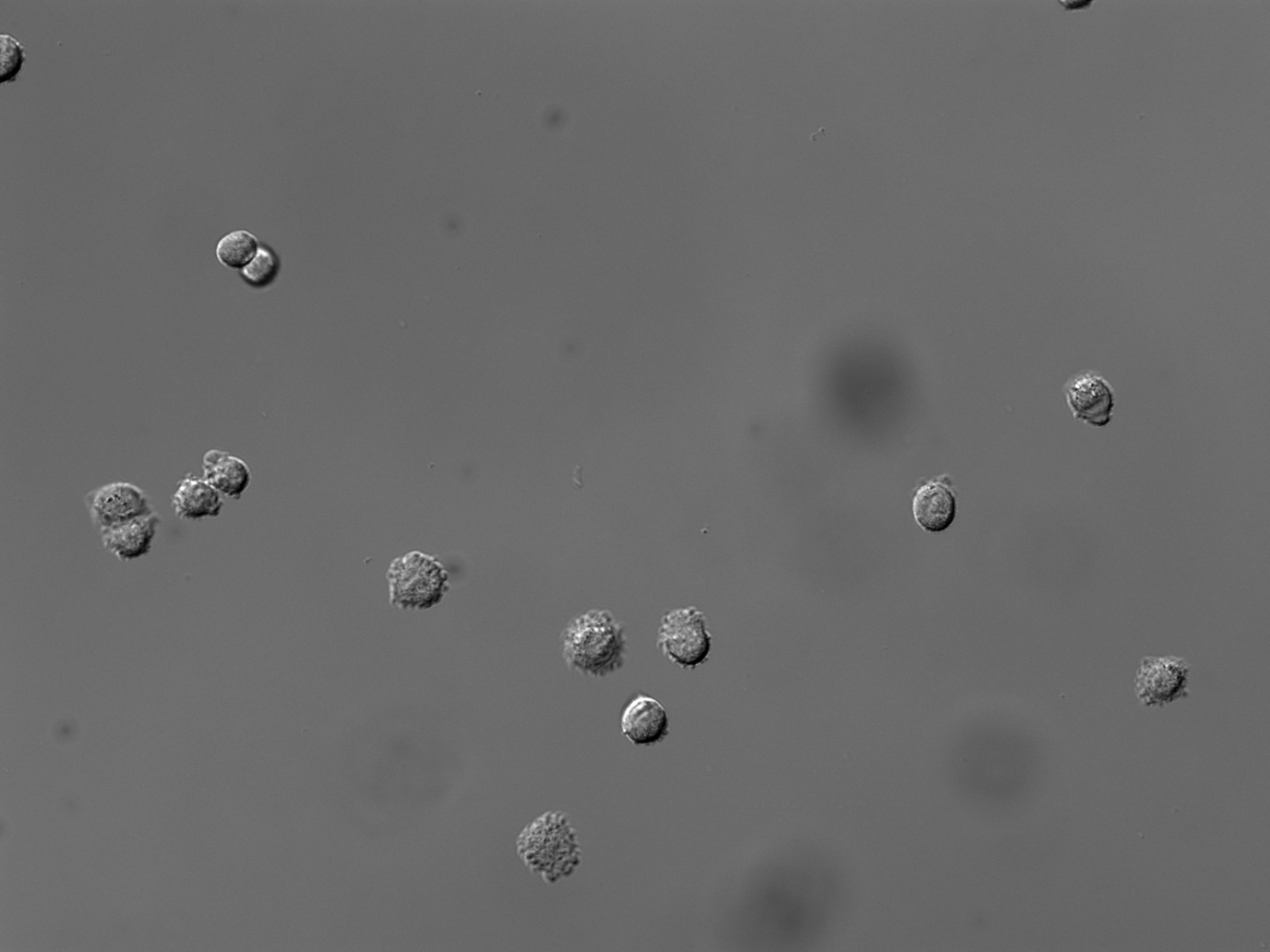

Supplement: Additional file 1: — Source codes of the proposed framework with test images. (ZIP 31244 kb) [file 12859_2017_1604_MOESM1_ESM.zip › Generalizing_Codes/cho04.png]

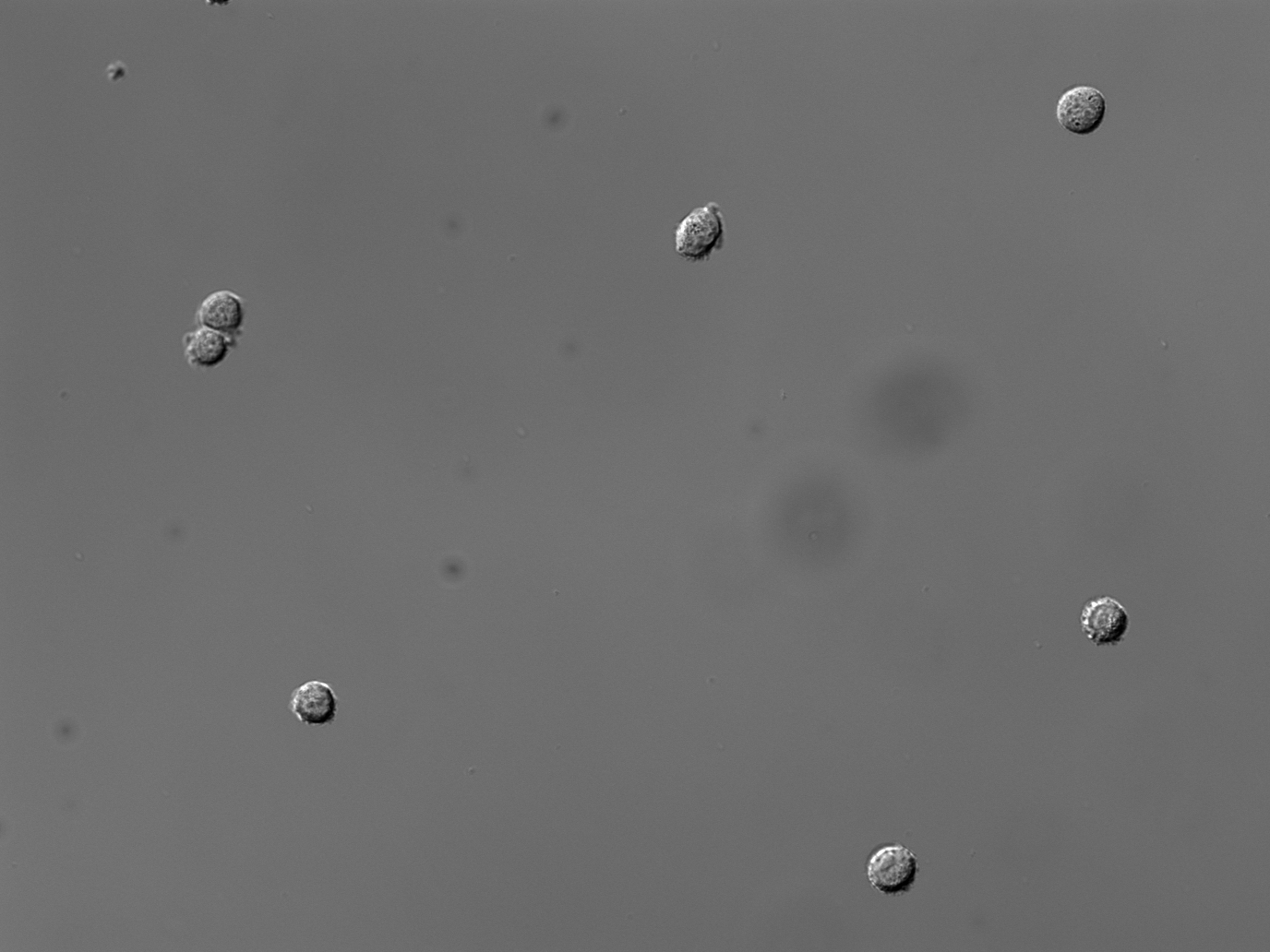

Supplement: Additional file 1: — Source codes of the proposed framework with test images. (ZIP 31244 kb) [file 12859_2017_1604_MOESM1_ESM.zip › Generalizing_Codes/cho05.png]

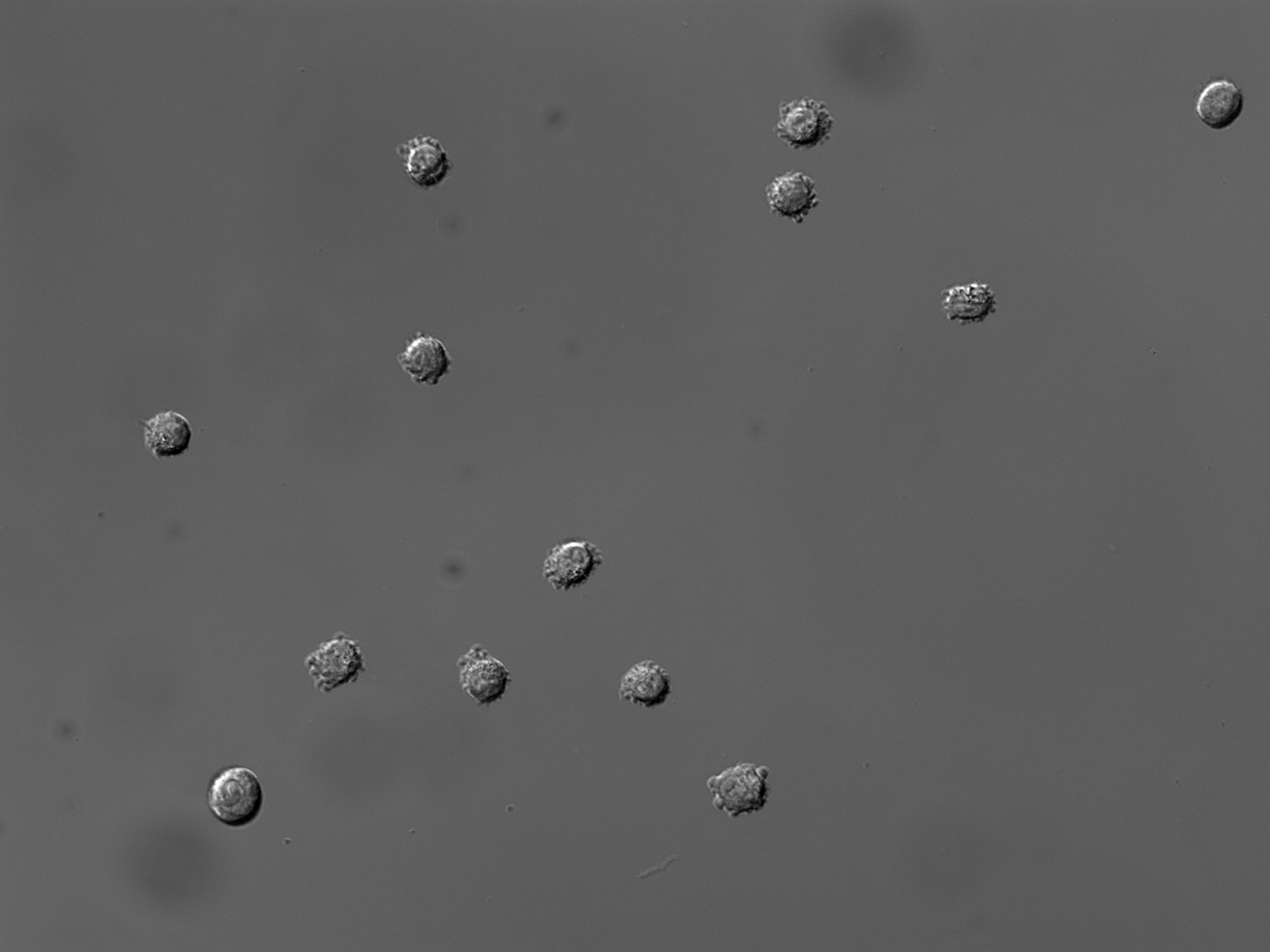

Supplement: Additional file 1: — Source codes of the proposed framework with test images. (ZIP 31244 kb) [file 12859_2017_1604_MOESM1_ESM.zip › Generalizing_Codes/cho06.png]

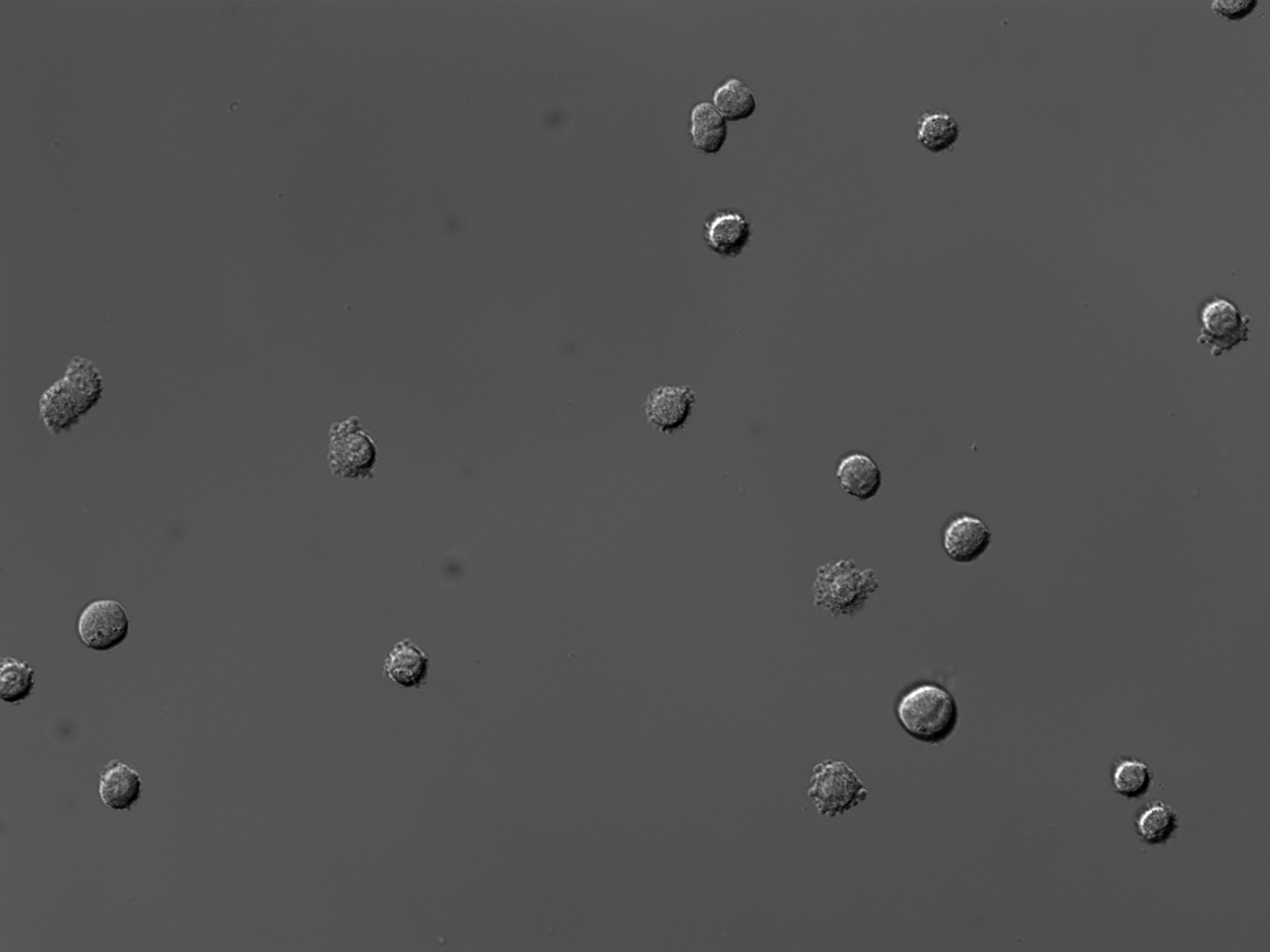

Supplement: Additional file 1: — Source codes of the proposed framework with test images. (ZIP 31244 kb) [file 12859_2017_1604_MOESM1_ESM.zip › Generalizing_Codes/cho07.png]

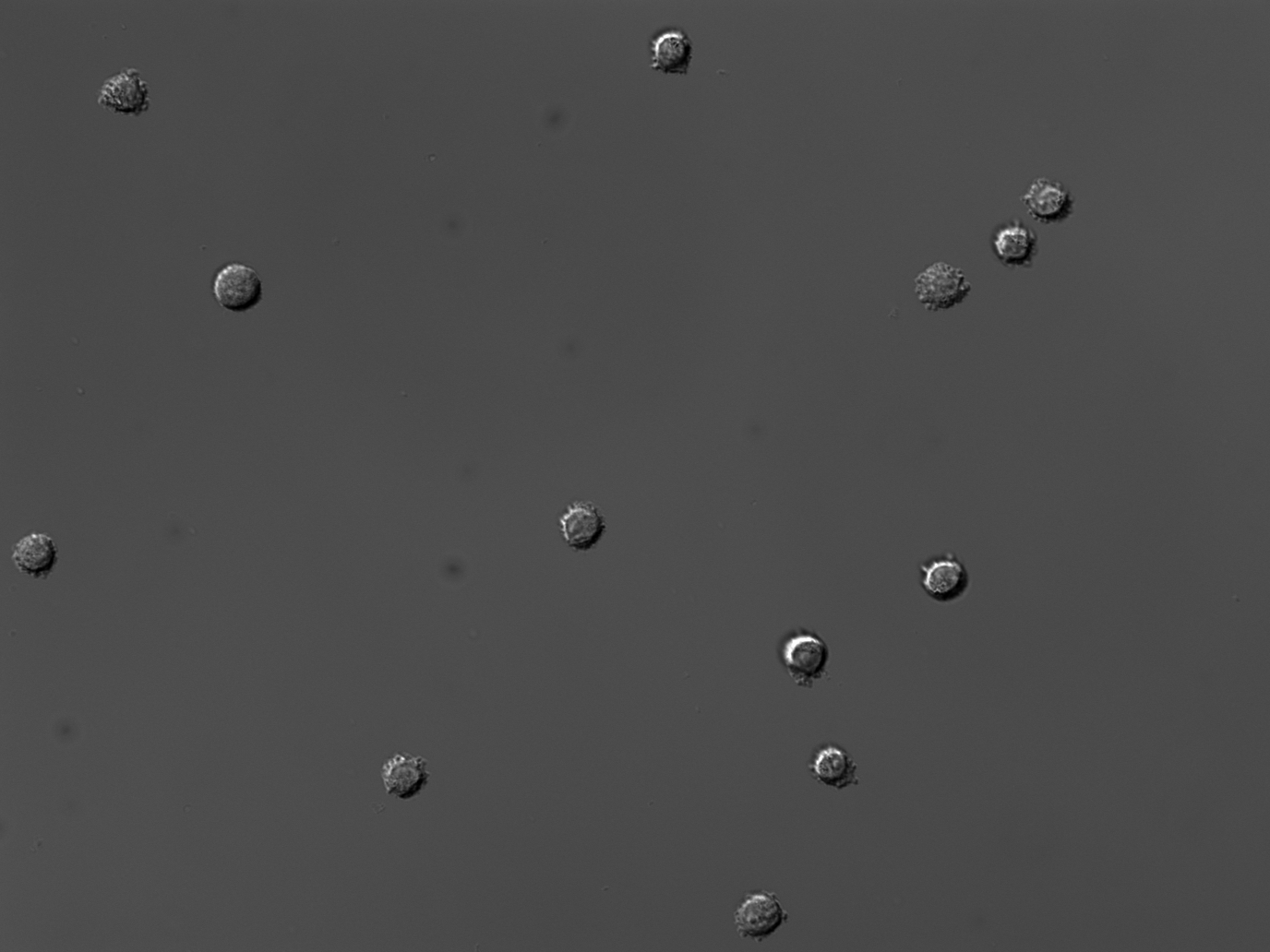

Supplement: Additional file 1: — Source codes of the proposed framework with test images. (ZIP 31244 kb) [file 12859_2017_1604_MOESM1_ESM.zip › Generalizing_Codes/cho08.png]

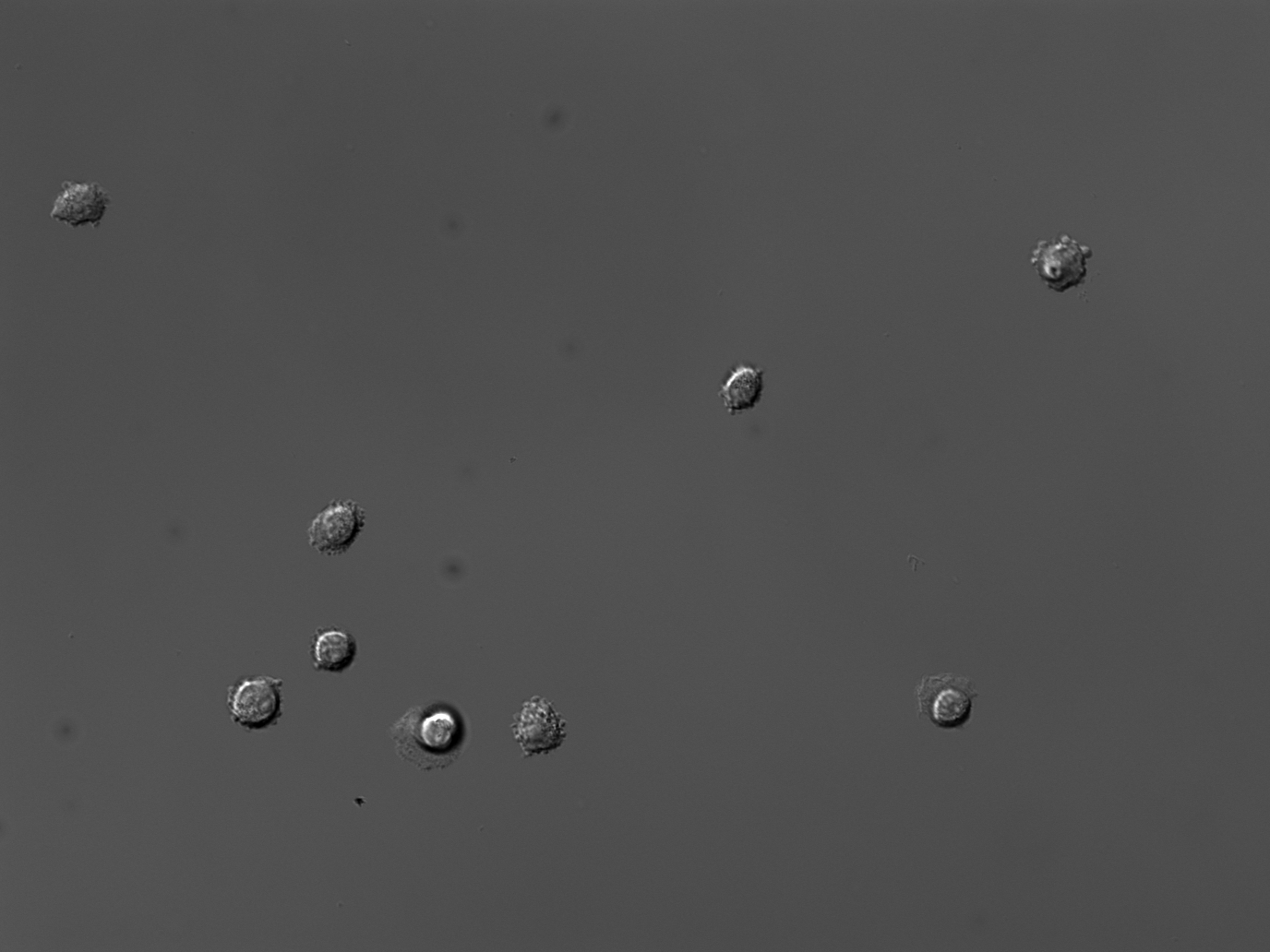

Supplement: Additional file 1: — Source codes of the proposed framework with test images. (ZIP 31244 kb) [file 12859_2017_1604_MOESM1_ESM.zip › Generalizing_Codes/cho09.png]

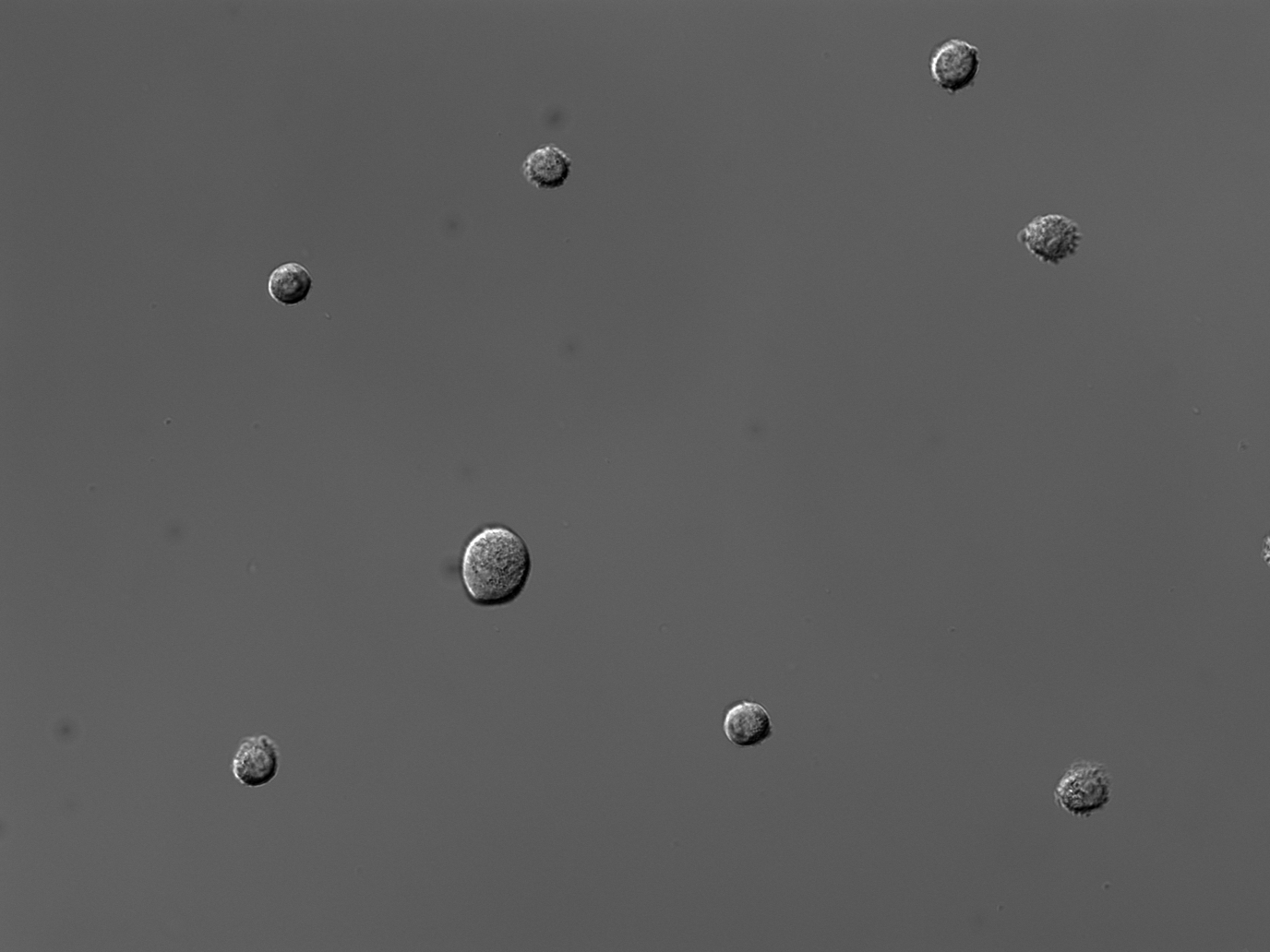

Supplement: Additional file 1: — Source codes of the proposed framework with test images. (ZIP 31244 kb) [file 12859_2017_1604_MOESM1_ESM.zip › Generalizing_Codes/cho10.png]

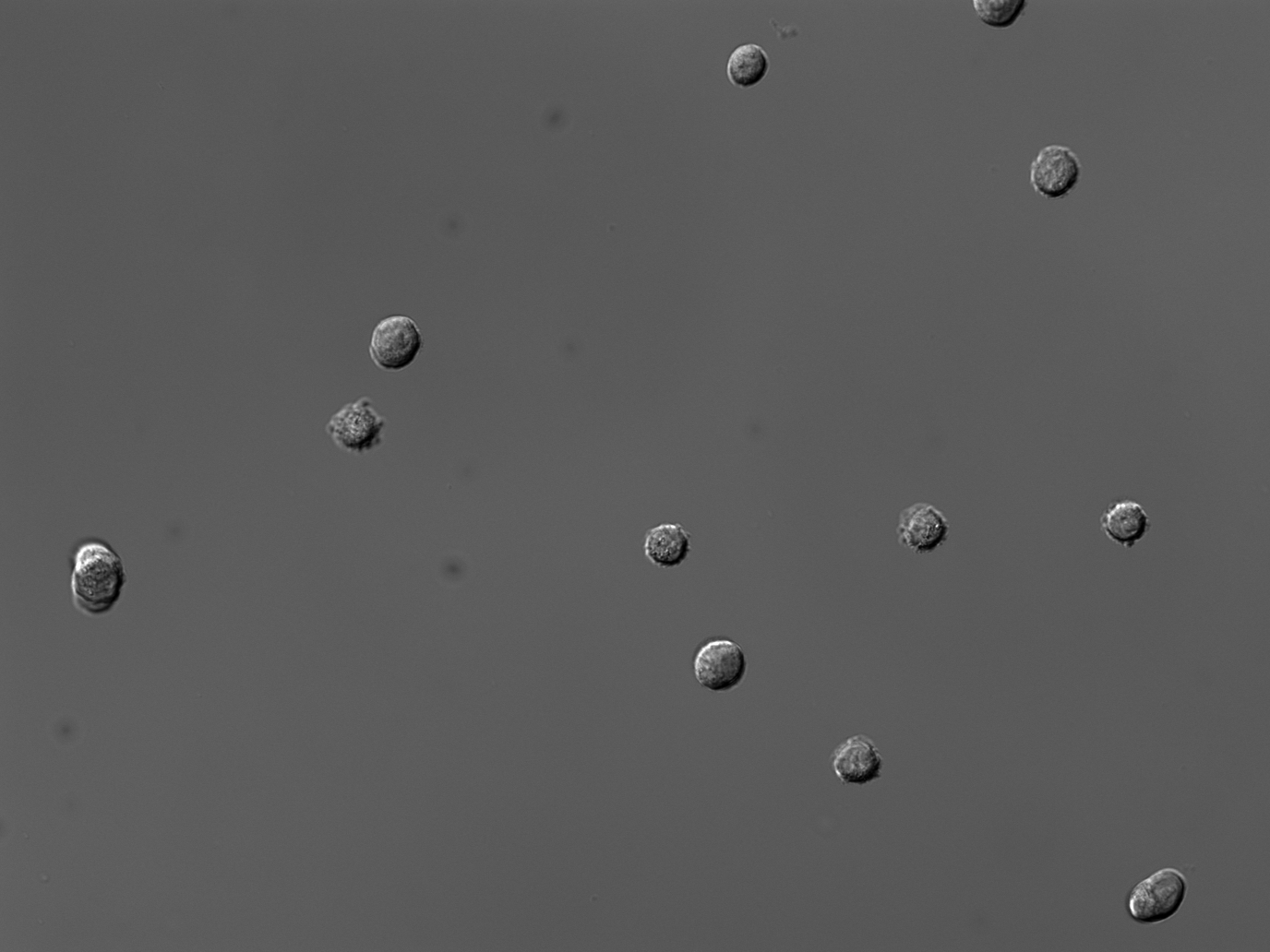

Supplement: Additional file 1: — Source codes of the proposed framework with test images. (ZIP 31244 kb) [file 12859_2017_1604_MOESM1_ESM.zip › Generalizing_Codes/cho11.png]

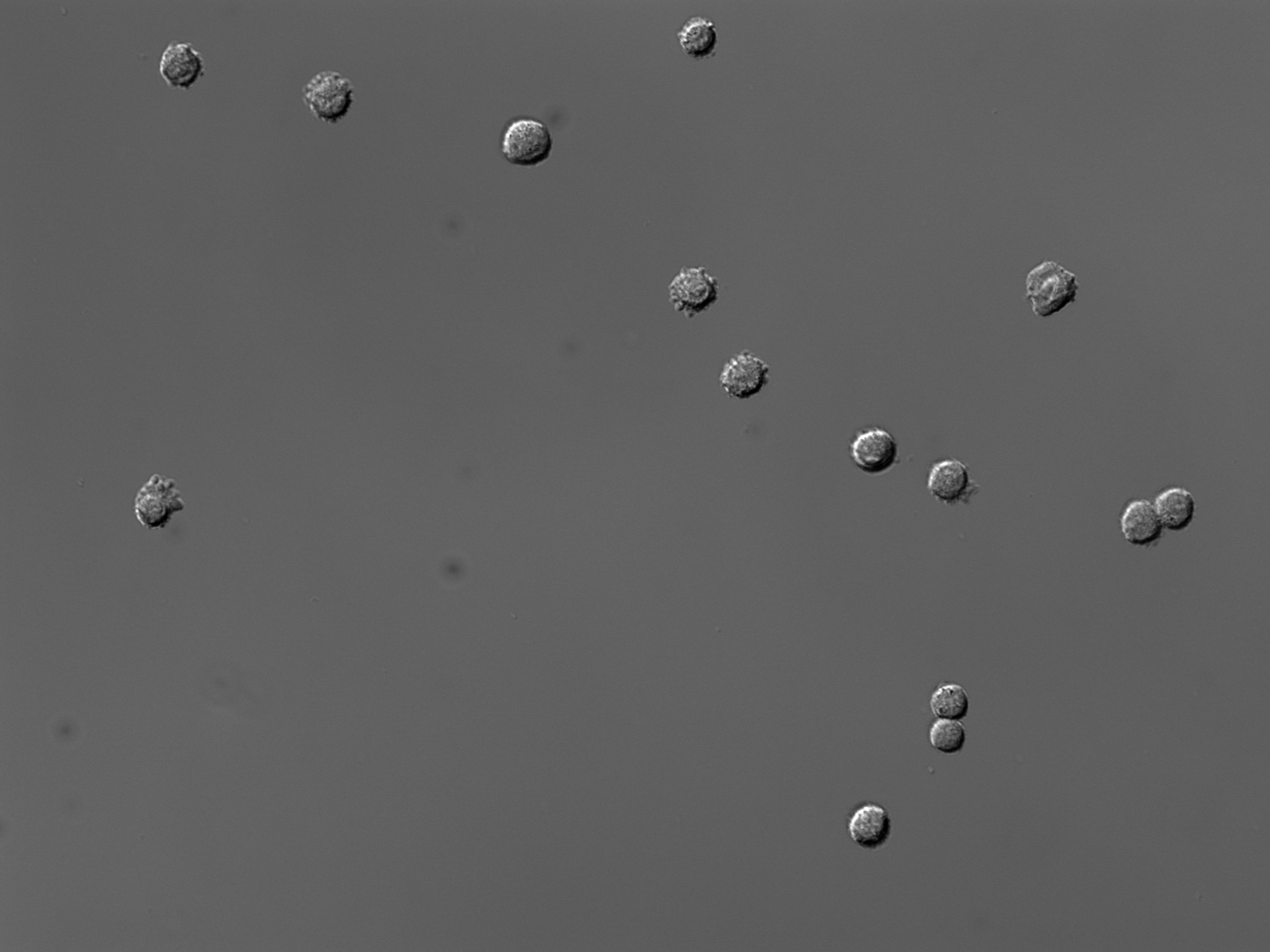

Supplement: Additional file 1: — Source codes of the proposed framework with test images. (ZIP 31244 kb) [file 12859_2017_1604_MOESM1_ESM.zip › Generalizing_Codes/cho12.png]

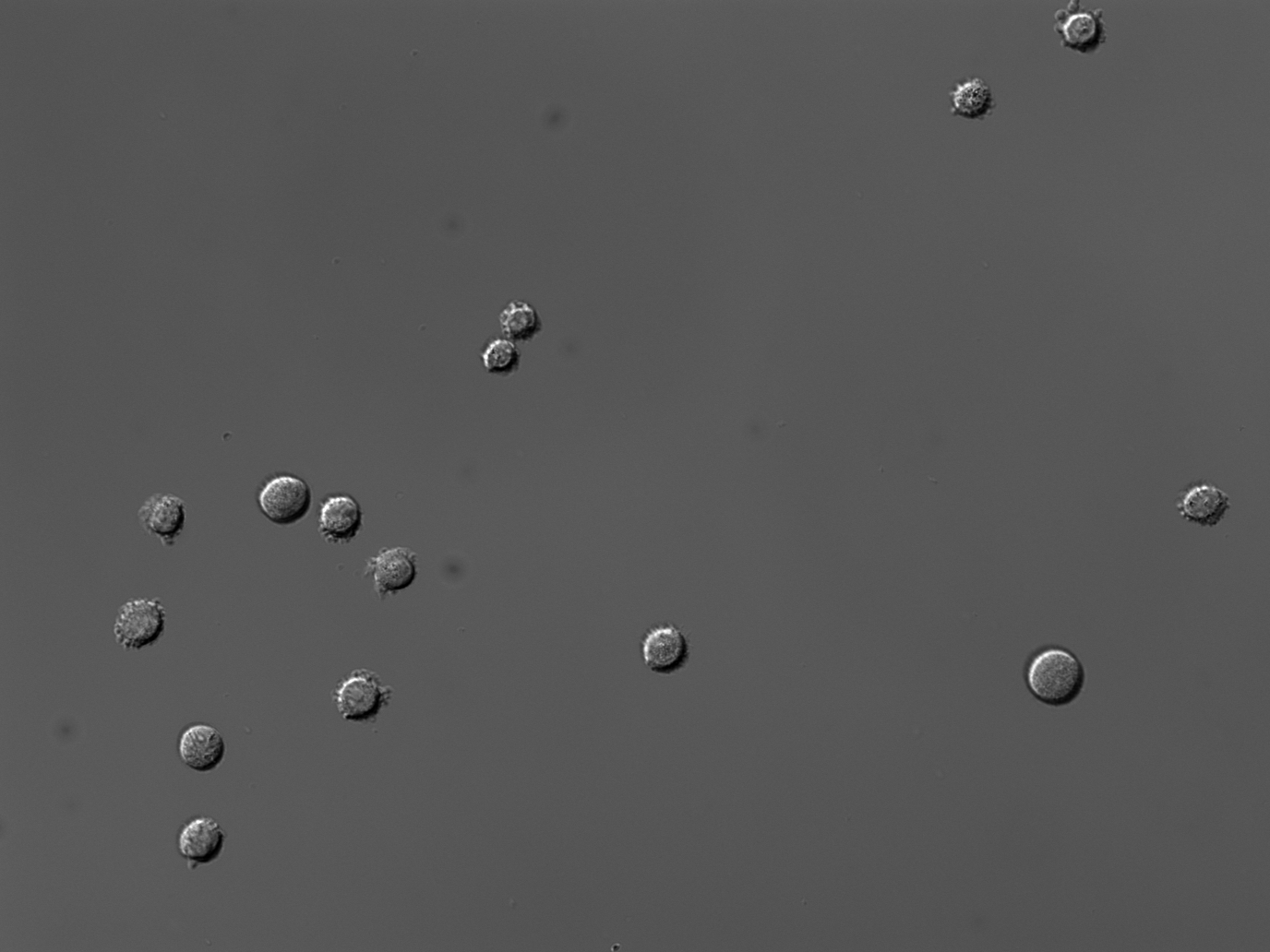

Supplement: Additional file 1: — Source codes of the proposed framework with test images. (ZIP 31244 kb) [file 12859_2017_1604_MOESM1_ESM.zip › Generalizing_Codes/cho13.png]

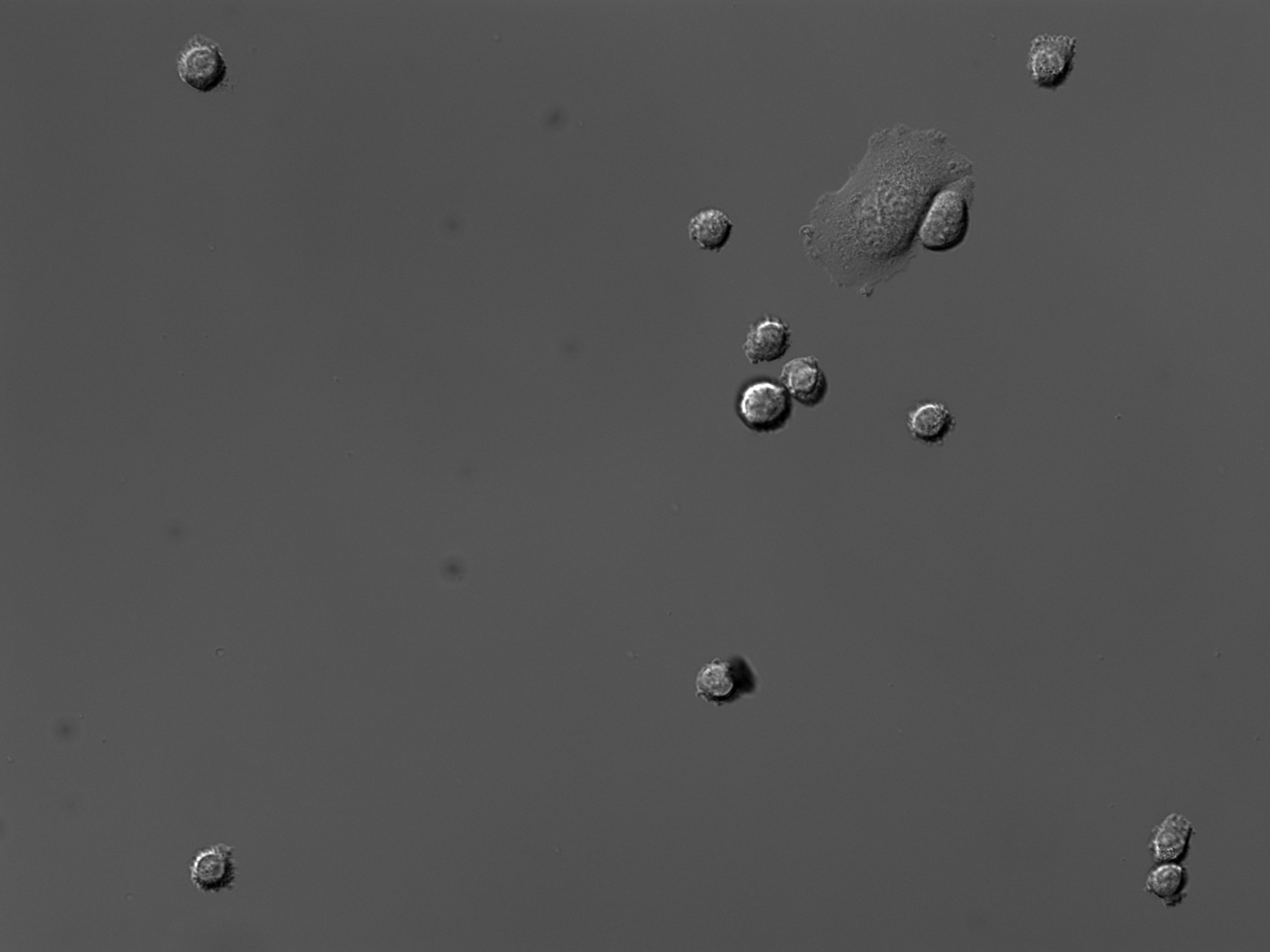

Supplement: Additional file 1: — Source codes of the proposed framework with test images. (ZIP 31244 kb) [file 12859_2017_1604_MOESM1_ESM.zip › Generalizing_Codes/cho14.png]

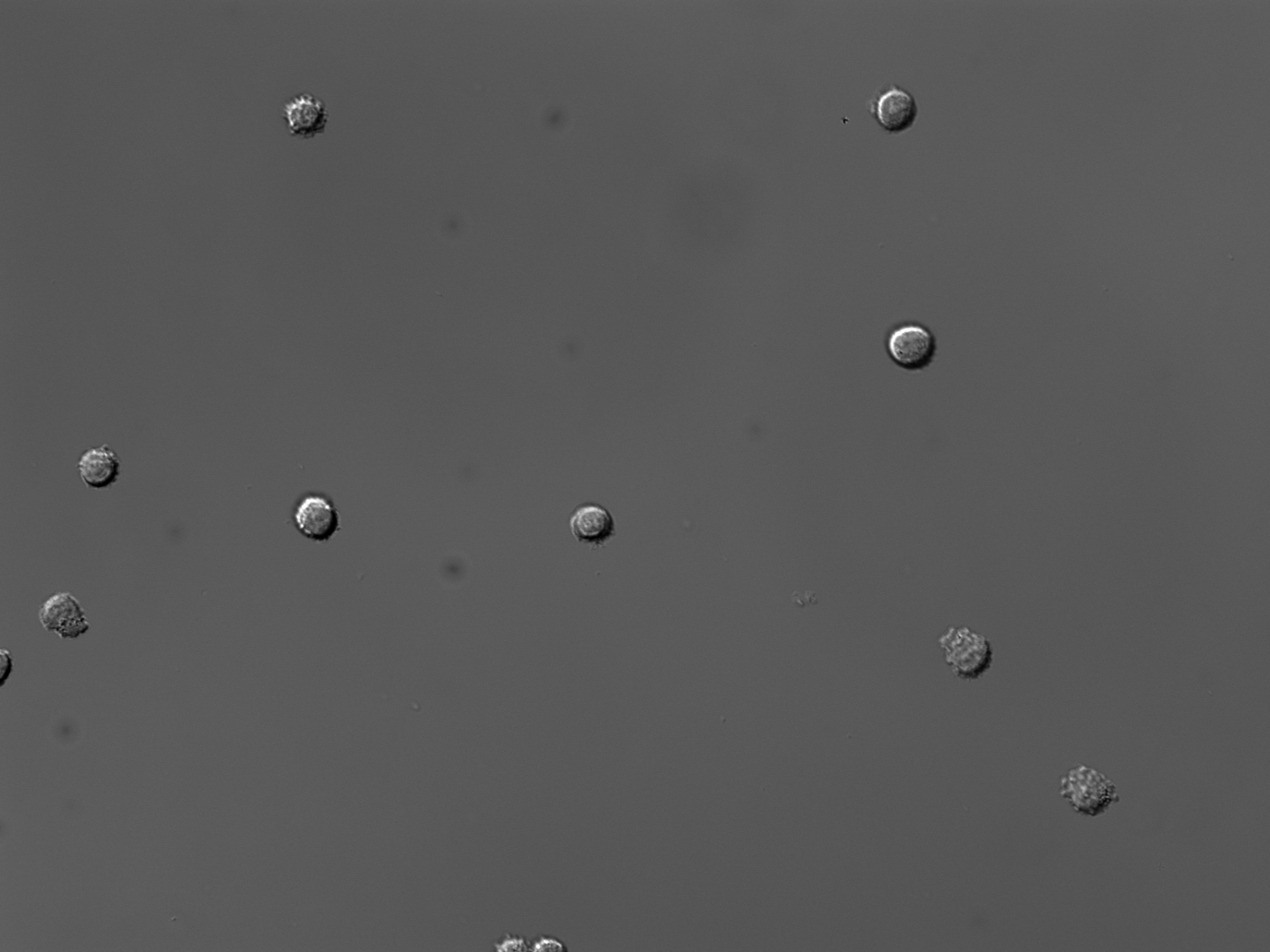

Supplement: Additional file 1: — Source codes of the proposed framework with test images. (ZIP 31244 kb) [file 12859_2017_1604_MOESM1_ESM.zip › Generalizing_Codes/cho15.png]

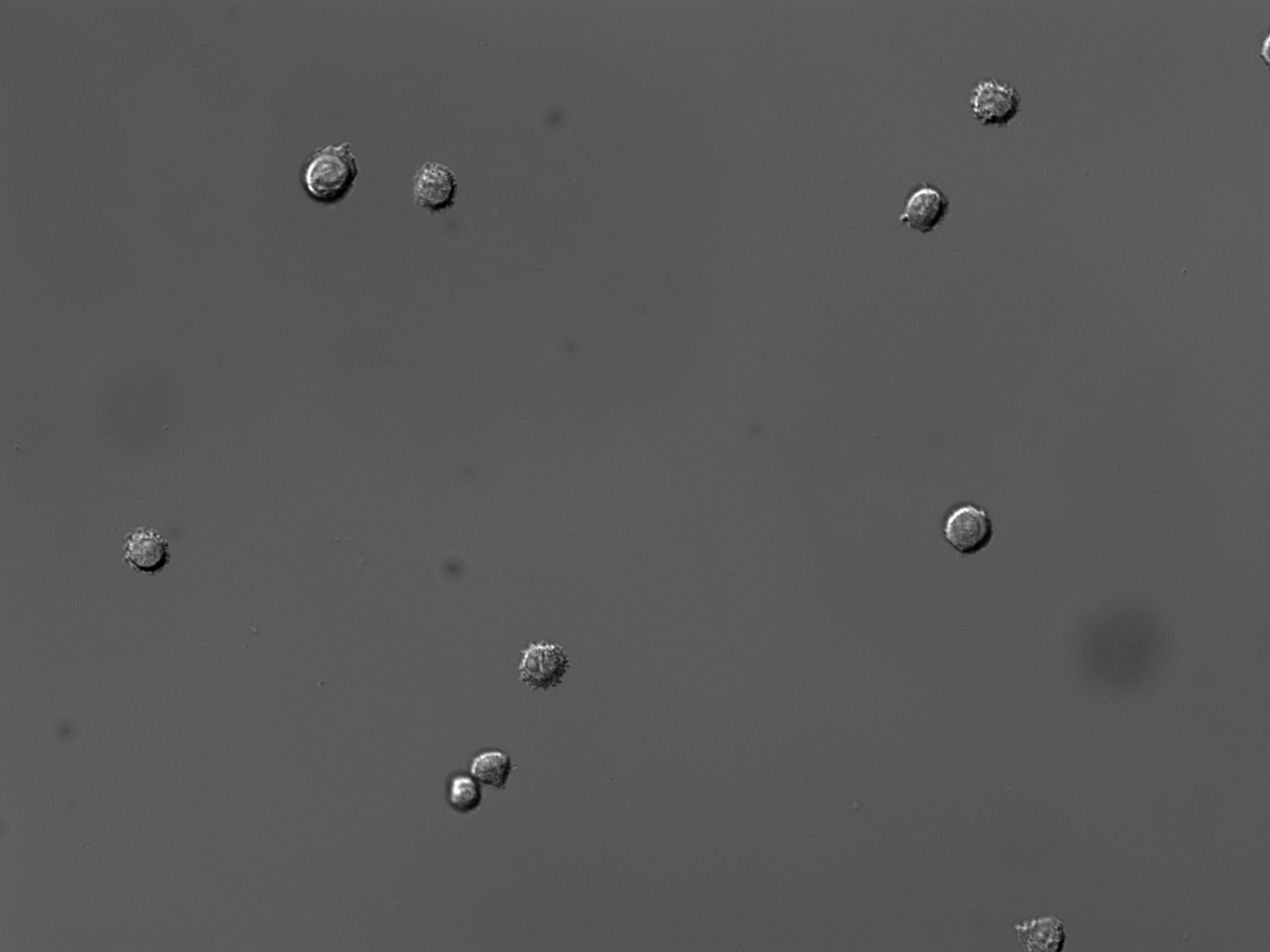

Supplement: Additional file 1: — Source codes of the proposed framework with test images. (ZIP 31244 kb) [file 12859_2017_1604_MOESM1_ESM.zip › Generalizing_Codes/cho16.png]

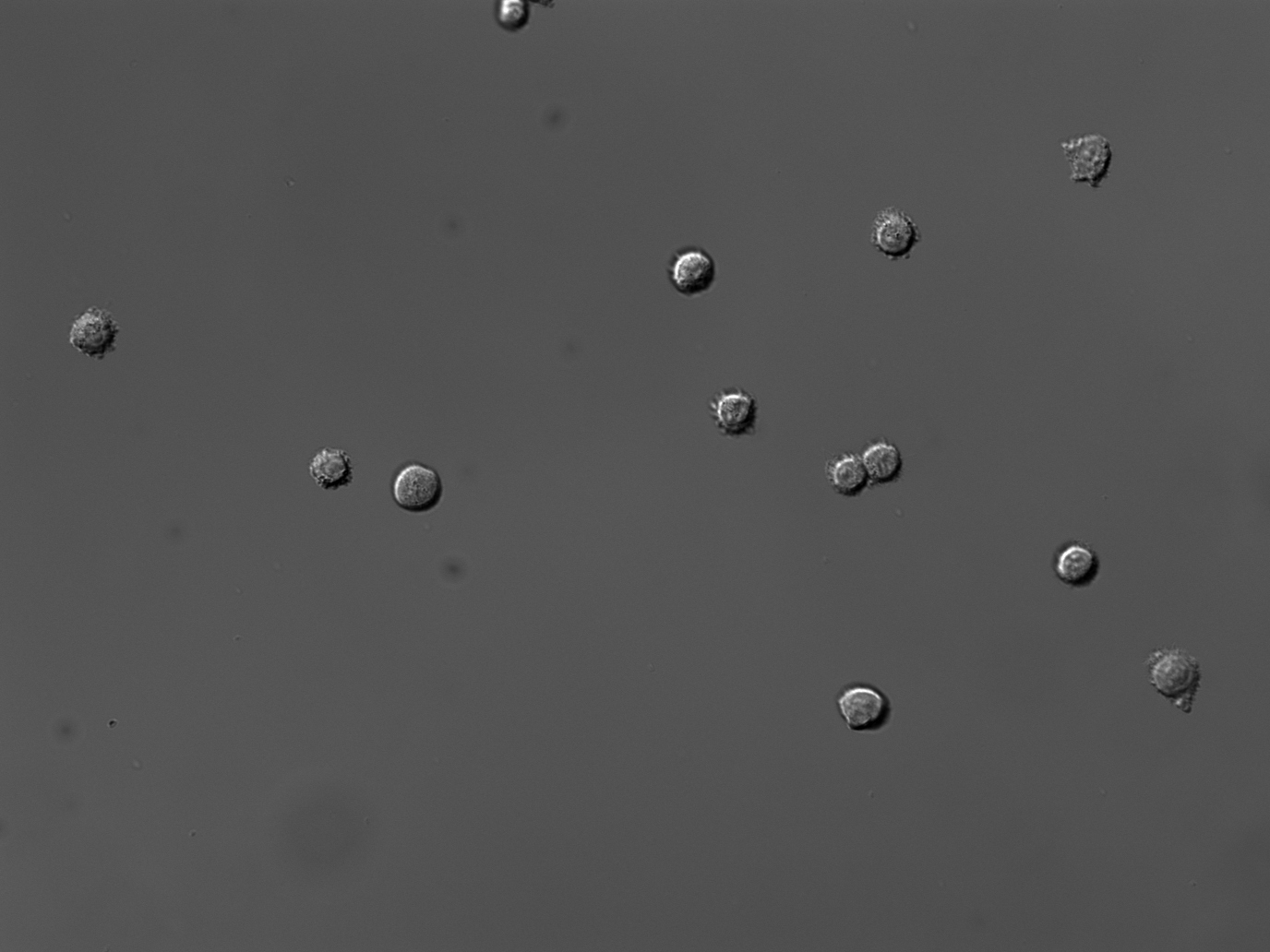

Supplement: Additional file 1: — Source codes of the proposed framework with test images. (ZIP 31244 kb) [file 12859_2017_1604_MOESM1_ESM.zip › Generalizing_Codes/cho17.png]

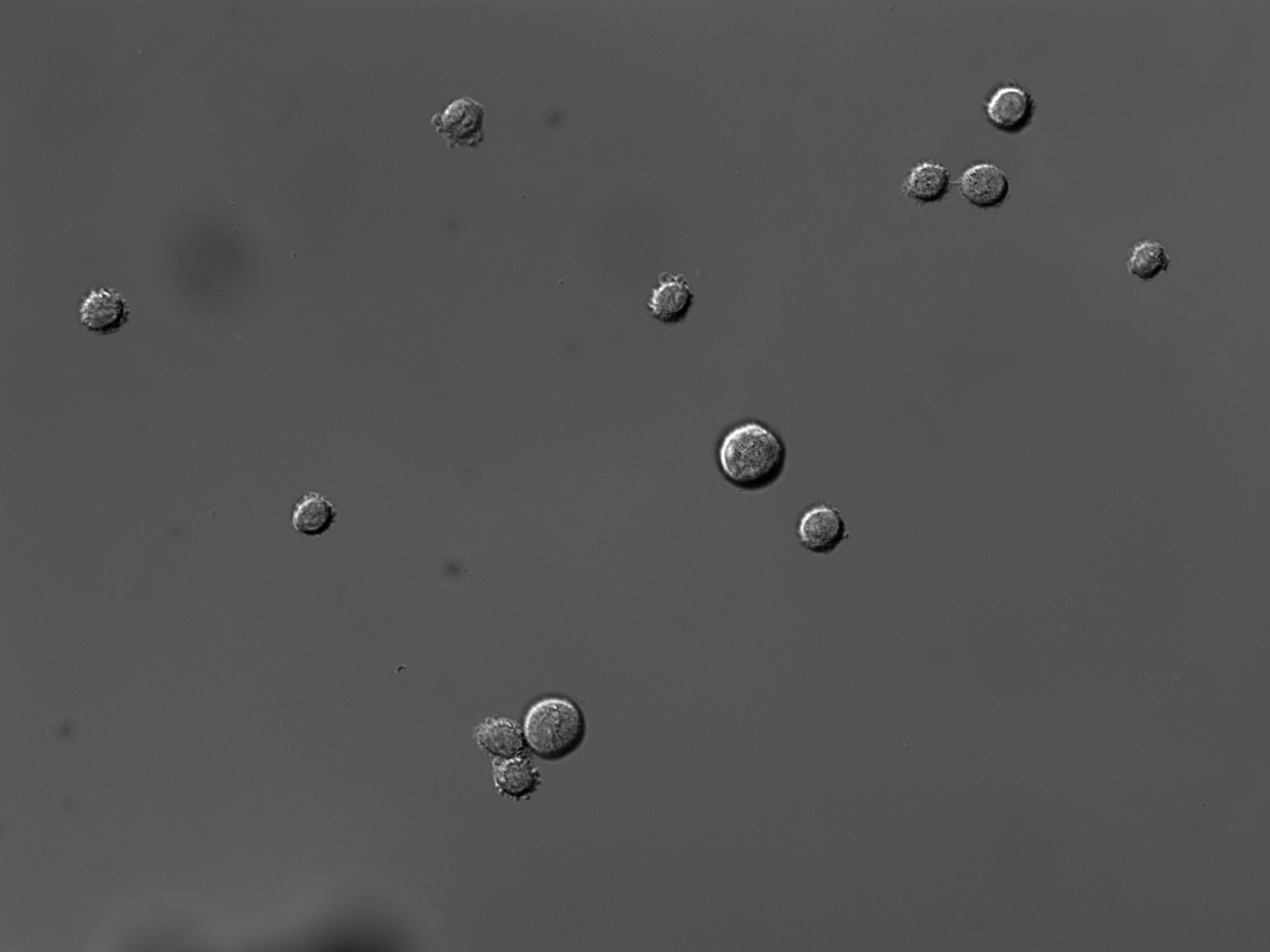

Supplement: Additional file 1: — Source codes of the proposed framework with test images. (ZIP 31244 kb) [file 12859_2017_1604_MOESM1_ESM.zip › Generalizing_Codes/cho18.png]

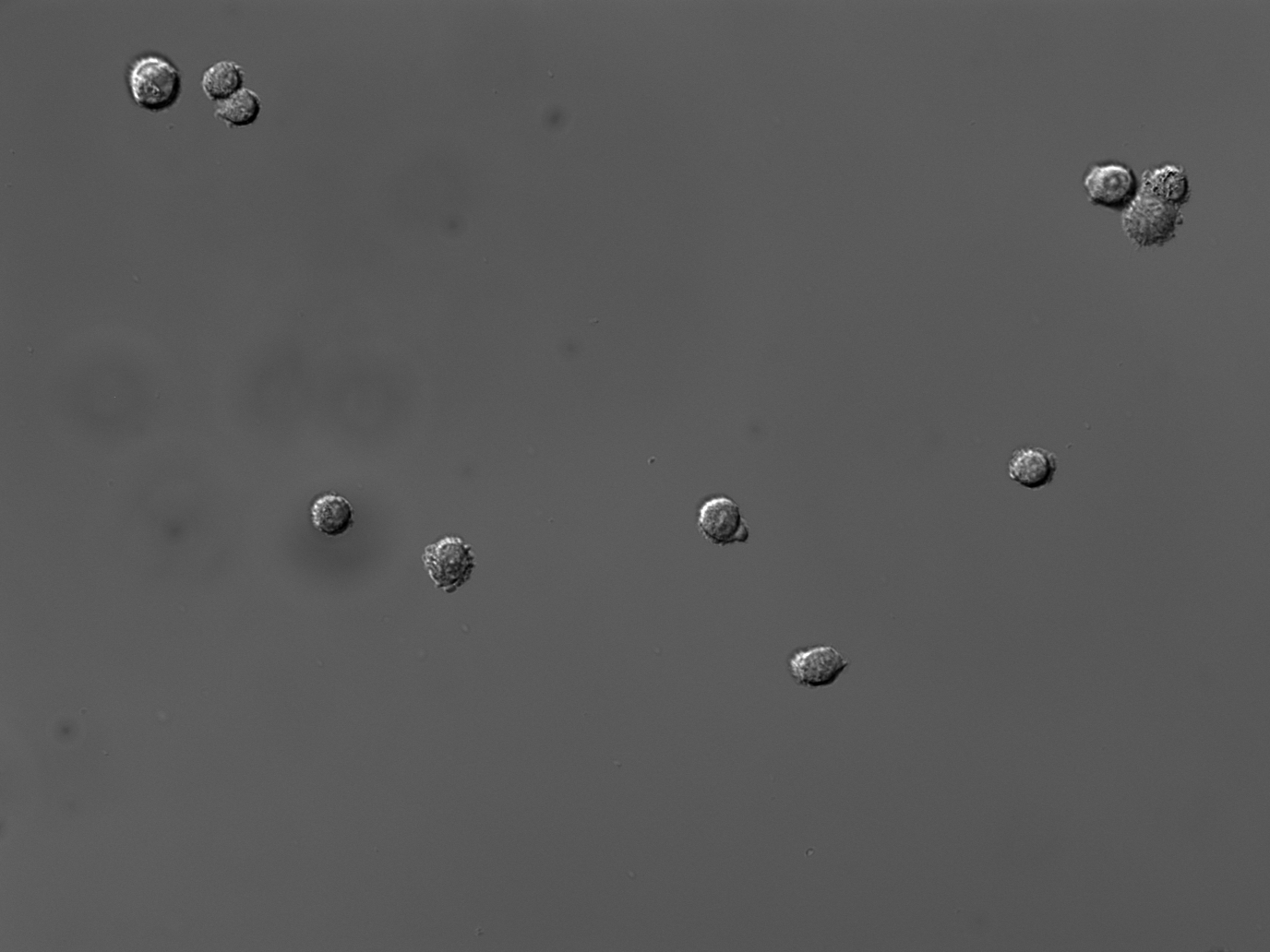

Supplement: Additional file 1: — Source codes of the proposed framework with test images. (ZIP 31244 kb) [file 12859_2017_1604_MOESM1_ESM.zip › Generalizing_Codes/cho19.png]

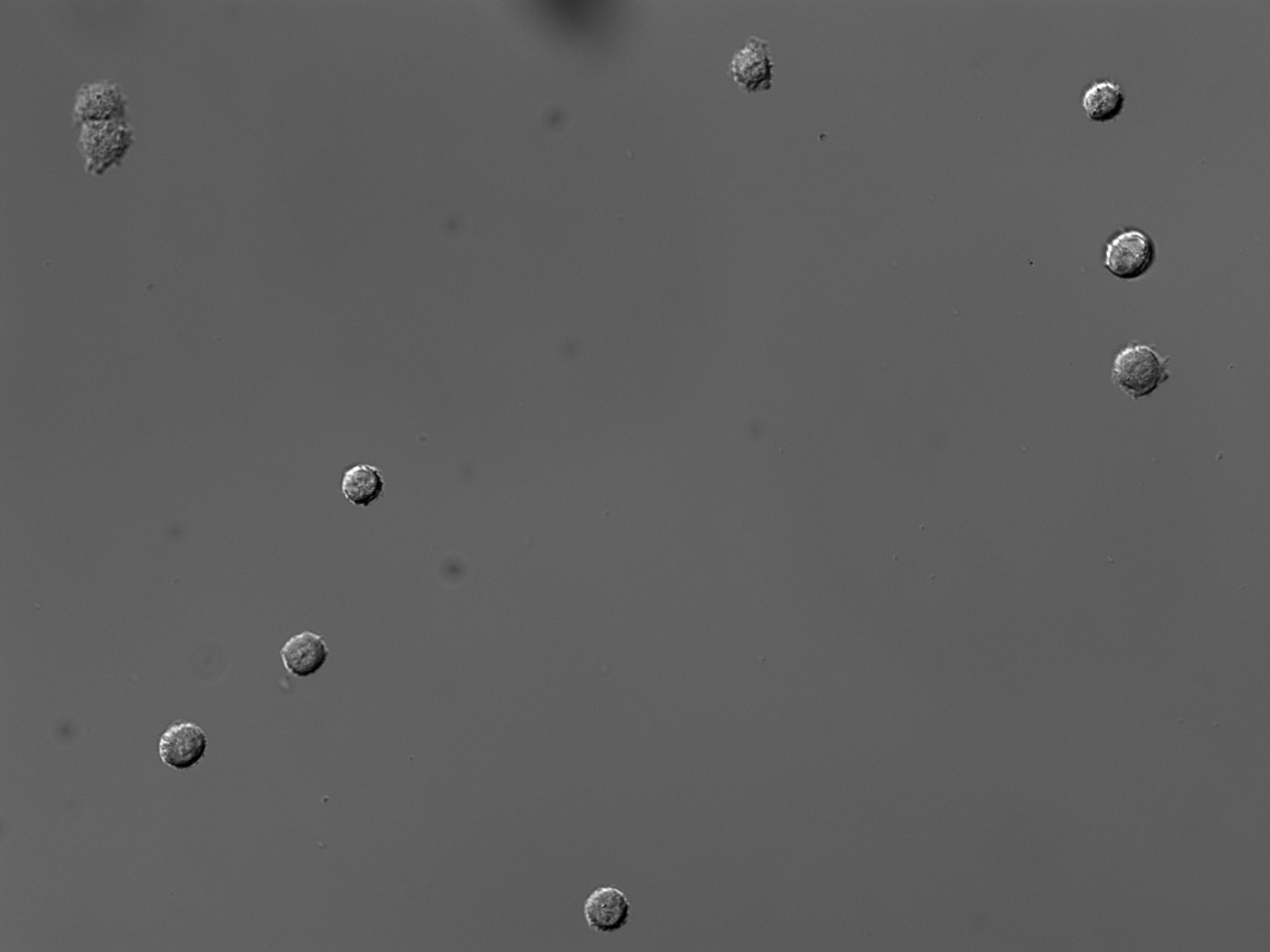

Supplement: Additional file 1: — Source codes of the proposed framework with test images. (ZIP 31244 kb) [file 12859_2017_1604_MOESM1_ESM.zip › Generalizing_Codes/cho20.png]

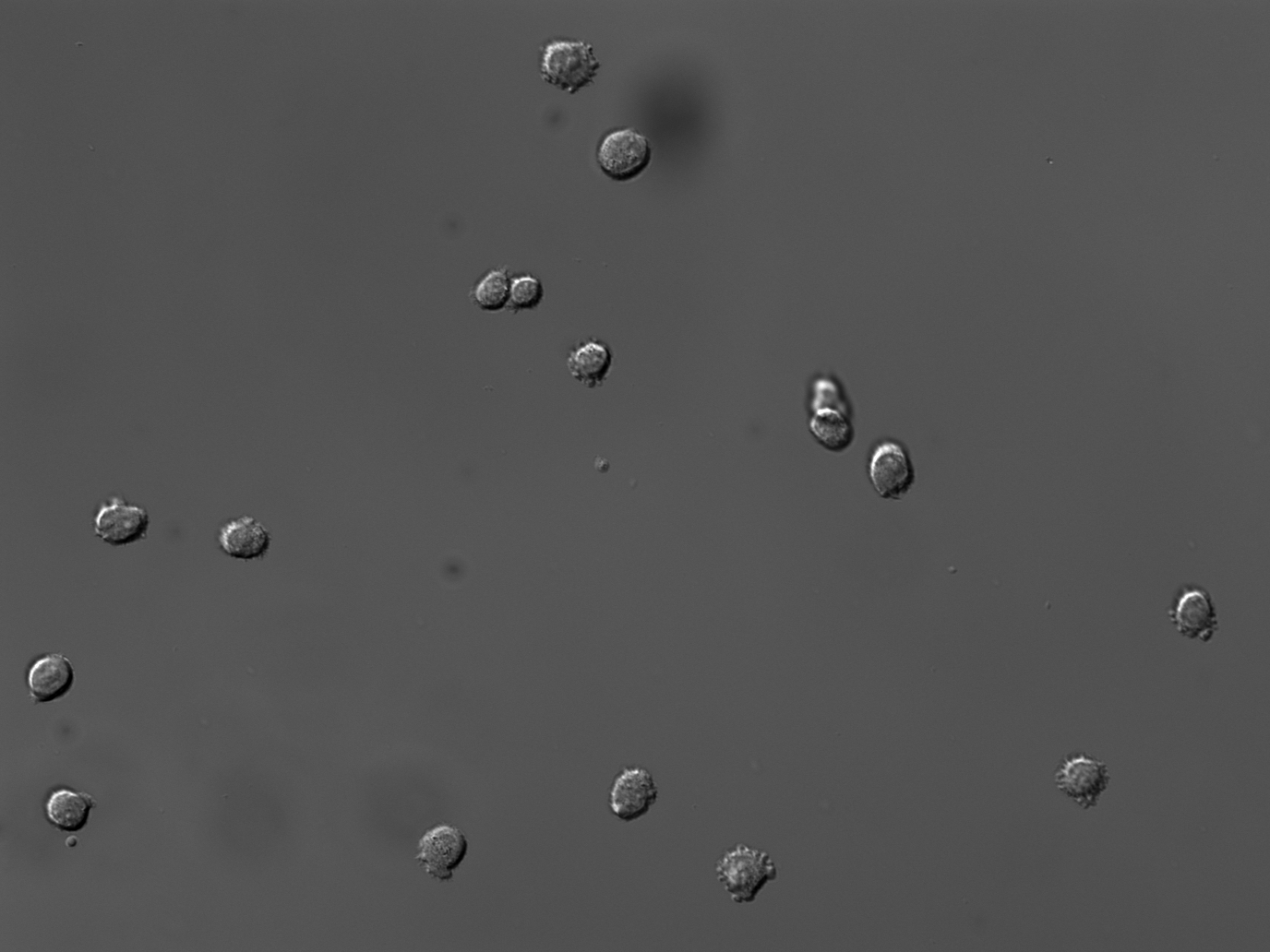

Supplement: Additional file 1: — Source codes of the proposed framework with test images. (ZIP 31244 kb) [file 12859_2017_1604_MOESM1_ESM.zip › Generalizing_Codes/cho60.png]

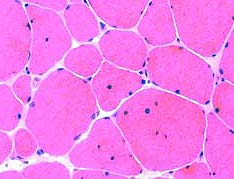

Supplement: Additional file 1: — Source codes of the proposed framework with test images. (ZIP 31244 kb) [file 12859_2017_1604_MOESM1_ESM.zip › Generalizing_Codes/muscle_images_for_comparison/m1.jpg]

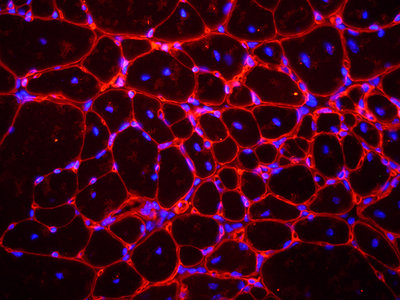

Supplement: Additional file 1: — Source codes of the proposed framework with test images. (ZIP 31244 kb) [file 12859_2017_1604_MOESM1_ESM.zip › Generalizing_Codes/muscle_images_for_comparison/m10.jpg]

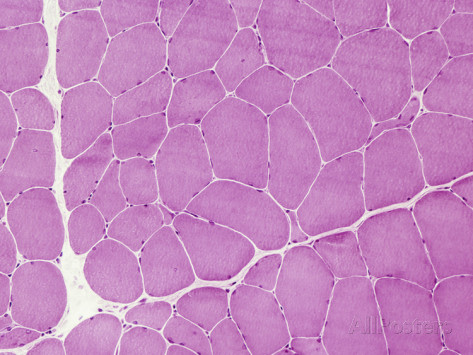

Supplement: Additional file 1: — Source codes of the proposed framework with test images. (ZIP 31244 kb) [file 12859_2017_1604_MOESM1_ESM.zip › Generalizing_Codes/muscle_images_for_comparison/m2.jpg]

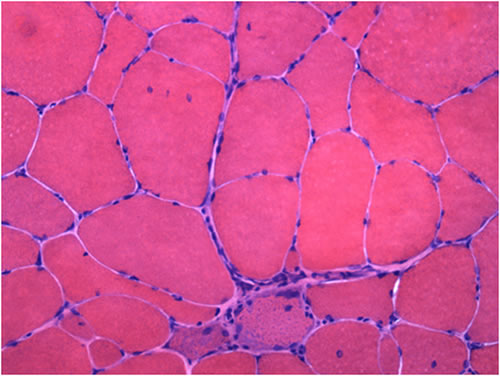

Supplement: Additional file 1: — Source codes of the proposed framework with test images. (ZIP 31244 kb) [file 12859_2017_1604_MOESM1_ESM.zip › Generalizing_Codes/muscle_images_for_comparison/m3.jpg]

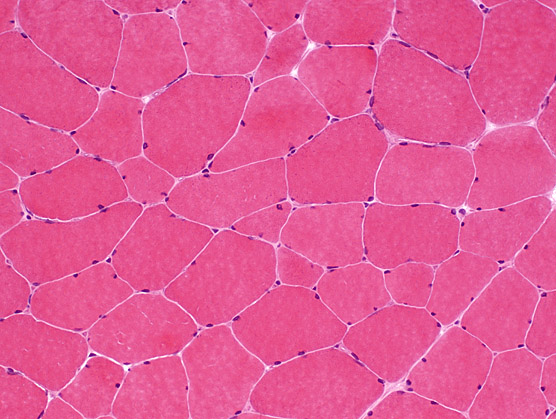

Supplement: Additional file 1: — Source codes of the proposed framework with test images. (ZIP 31244 kb) [file 12859_2017_1604_MOESM1_ESM.zip › Generalizing_Codes/muscle_images_for_comparison/m4.jpg]

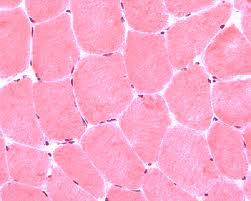

Supplement: Additional file 1: — Source codes of the proposed framework with test images. (ZIP 31244 kb) [file 12859_2017_1604_MOESM1_ESM.zip › Generalizing_Codes/muscle_images_for_comparison/m5.jpg]

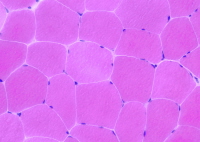

Supplement: Additional file 1: — Source codes of the proposed framework with test images. (ZIP 31244 kb) [file 12859_2017_1604_MOESM1_ESM.zip › Generalizing_Codes/muscle_images_for_comparison/m6.jpg]

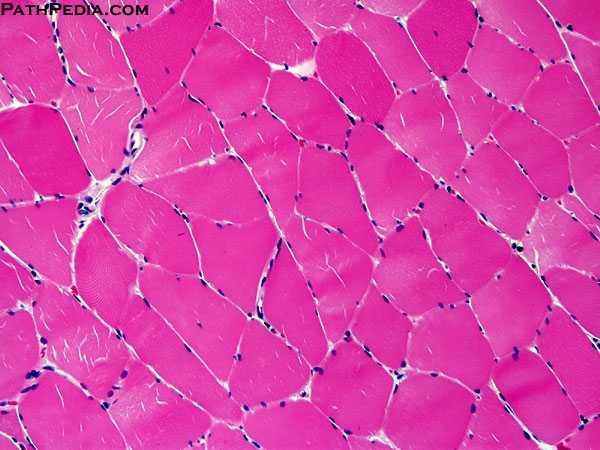

Supplement: Additional file 1: — Source codes of the proposed framework with test images. (ZIP 31244 kb) [file 12859_2017_1604_MOESM1_ESM.zip › Generalizing_Codes/muscle_images_for_comparison/m8.jpg]

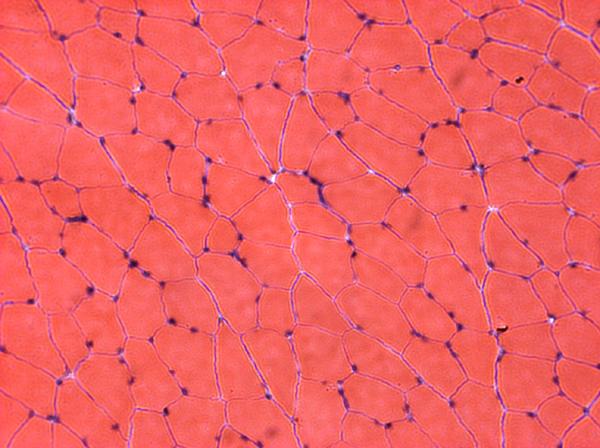

Supplement: Additional file 1: — Source codes of the proposed framework with test images. (ZIP 31244 kb) [file 12859_2017_1604_MOESM1_ESM.zip › Generalizing_Codes/muscle_images_for_comparison/m9.jpg]

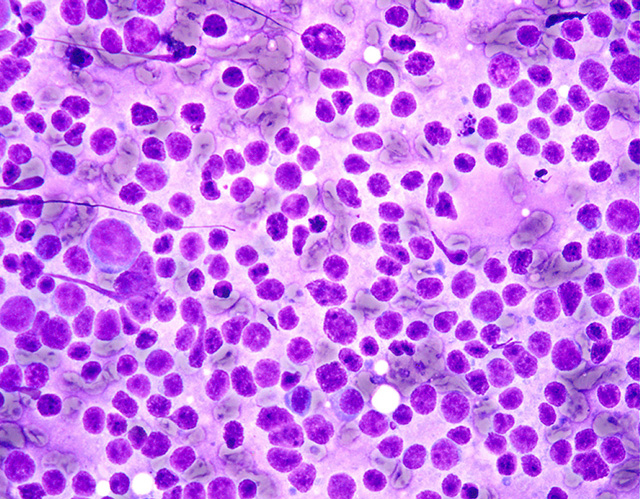

Supplement: Additional file 1: — Source codes of the proposed framework with test images. (ZIP 31244 kb) [file 12859_2017_1604_MOESM1_ESM.zip › Generalizing_Codes/t4.jpg]
